# The potential of generative AI for personalized persuasion at scale

**DOI:** 10.1038/s41598-024-53755-0

**Published:** 2024-02-26

**Authors:** S. C. Matz, J. D. Teeny, S. S. Vaid, H. Peters, G. M. Harari, M. Cerf

**Affiliations:** 1 Columbia Business School, New York, USA; 2 Center for Advanced Technology and Human Performance, Columbia Business School, New York, USA; 3 Kellogg School of Management, Evanston, USA; 4 Negotiation, Organizations and Marketing Unit, Department of Communication, Harvard Business School, Stanford University, Stanford, USA; 5 Present Address: Department of Communication, Stanford University, Stanford, USA

**Keywords:** Human behaviour, Psychology

## Abstract

Matching the language or content of a message to the psychological profile of its recipient (known as “personalized persuasion”) is widely considered to be one of the most effective messaging strategies. We demonstrate that the rapid advances in large language models (LLMs), like ChatGPT, could accelerate this influence by making personalized persuasion scalable. Across four studies (consisting of seven sub-studies; total *N* = 1788), we show that personalized messages crafted by ChatGPT exhibit significantly more influence than non-personalized messages. This was true across different domains of persuasion (e.g., marketing of consumer products, political appeals for climate action), psychological profiles (e.g., personality traits, political ideology, moral foundations), and when only providing the LLM with a single, short prompt naming or describing the targeted psychological dimension. Thus, our findings are among the first to demonstrate the potential for LLMs to automate, and thereby scale, the use of personalized persuasion in ways that enhance its effectiveness and efficiency. We discuss the implications for researchers, practitioners, and the general public.

## Introduction

Financial analysts have described people’s digital behavioral data as “more valuable than oil”^1,2^. This is, in part, because such records afford one of the most effective forms of influence: *personalized persuasion*^3,4^. Compared to non-personalized communication, matching the content of a persuasive message (e.g., its language or visuals) to the psychological profile of its recipient enhances its effectiveness (e.g.,^4,5^). On the one hand, such personalization offers tremendous opportunities to promote desired behaviors, including a healthy lifestyle^6–8^, financial saving^9^, or support for environmentalism^10^. On the other hand, it can have a pernicious effect on societies^11^, for example, increasing the spread of disinformation^12^, manipulating political preferences^13,14^, or promoting maladaptive consumer decision-making^15,16^. We provide the first empirical evidence demonstrating how content generated by artificial intelligence (AI) can scale personalized persuasion by automating the creation of such messages with only limited information about the message recipient. As legislators increasingly consider whether (and how) to regulate generative AI^17^, our work suggests that AI-automated, personalized persuasion is poised to create an inflection point for the implementation and effectiveness of this influence tactic.

Up to this moment in time, the design and delivery of personalized persuasion in real-world conditions have been constrained by two procedural steps: (1) the identification of a target’s psychological profile, and (2) the crafting of a message that resonates with that profile. In recent years, the growing availability of people’s digital footprints in combination with novel machine learning tools has enabled researchers and practitioners to automate the first step. For example, instead of relying on self-report measures to assess people’s psychological traits (e.g., personality), predictive algorithms can estimate these traits directly from their digital behaviors^17–19^, including their Facebook Likes^19,20^, the language used in their social media posts^21–25^, their profile pictures^26^, their credit card spending^27,28^, and their smartphone sensing data^29^.

Research suggests that such automated predictions can indeed accelerate the implementation of personalized persuasion^5,30^. However, the second step of this influence tactic—crafting a message that matches the identified psychological profile—has continued to require the labor- and time-intensive process of human authorship (i.e., human creators must develop and design the persuasive messages that match the targeted psychology). In this paper, we empirically test the effectiveness of using large language models (LLMs^31–35^)—specifically, OpenAI’s widely used ChatGPT^35^—to author text-based, psychologically-tailored persuasion.

LLMs are advanced generative AI systems that use transformer neural network architectures^36^ to learn language representations from vast corpora of text data. LLMs can use these representations to generate text based on probabilistic estimates for which words or groups of words would be most expected in response to a particular text-based prompt. Since their inception, LLMs have shown rapid performance improvements in a variety of natural language processing tasks^37^. In addition, applications that are optimized for human interaction with LLMs (e.g., chat.opaenai.com) have made them accessible to the general public, with ChatGPT becoming the fastest platform to reach 100 million monthly active users^38^.

Scientists and practitioners have been quick to acknowledge the potential power of LLMs in the context of persuasion^39–41^. For example, ad agencies have started to employ LLMs to create generic “ad copy” that can be published quickly^42^. Similarly, recent research suggests that automatically generated product descriptions in combination with human screening can result in improved click-through and conversion rates in e-commerce sites^43^. While these developments speak to the ability of LLMs to generate *generic* persuasive content, they do not offer any insights into (1) whether LLMs can create persuasive messages that are *personalized* to the needs and motivations of an individual and (2) whether doing so indeed makes these persuasive attempts more influential.

We expect LLMs to be able to do so for several reasons. First, LLMs have been shown to bear an uncanny resemblance to humans in how they process information and respond to external stimuli (e.g.,^44^). For example, recent work suggests that a central psychological process in personalized persuasion, theory of mind (e.g., representing other people’s mental states), may have spontaneously emerged in LLMs (e.g.,^45,46^). Moreover, whereas humans are known to be prone to egocentrism biases when crafting persuasive messages—i.e., producing arguments that are persuasive to themselves, rather than the other person^47^—algorithms do not suffer from the same limitations, making LLMs prime candidates for the creation of personalized persuasive content. Second, because LLMs have been trained on expansive corpora of human-generated language, they have access to a far greater and more diverse range of human expressions than any single human author could ever process. In combination, these two features make it likely that LLMs are not only able to discern the meaning of psychological constructs, but that they will also be able to integrate their vast “knowledge” of them into the generation of persuasive personalized messages. If this prediction is true, outsourcing personalized persuasion to machines could not only increase its efficiency and scalability, but also its effectiveness.

Across four studies consisting of seven individual sub-studies, we provide some of the first empirical evidence that LLMs can “close the loop” in automating the design and implementation of personalized persuasion. Specifically, we show that Open AI’s ChatGPT is capable of generating personalized persuasion that is effective in shaping people’s attitudes and intended behaviors. To highlight the breadth and generalizability of our findings, we replicate the effect across multiple prominent persuasion domains (i.e., consumer marketing, political appeals, and health messaging), as well as a variety of psychological traits that reflect different but common aspects of a person’s psychological profile (i.e., Big Five personality traits, regulatory focus, political orientation, and moral foundations).

The studies received ethical approval from Columbia University’s IRB (Protocol #: AAAU4108) and were performed in accordance with relevant guidelines and regulations. All participants provided informed consent at the beginning of the study. Materials, data, and analysis scripts are available on OSF (link: https://osf.io/79wcm/).

## Studies 1a and 1b

Studies 1a and 1b investigated whether personality-tailored messages generated by the pre-trained Transformer model ChatGPT-3 can increase the messages’ perceived persuasiveness. In addition, Study 1b tested whether the effect was impacted by people’s awareness that the messages were generated using AI and designed to speak to specific personality traits. We focused on the Big Five personality traits as an established marker of personality^48^ that has been: (1) validated across different contexts^49^, (2) shown to predict a wide range of preferences and life outcomes^41,42^, and (3) used in past research on personalized persuasion (e.g.,^50–52^). The Big Five model posits that individual differences in cognition, affect, and behavior can be pragmatically described using the following five dimensions: Openness, Conscientiousness, Extraversion, Agreeableness and Neuroticism^48,53^.

### Methods Study 1a

In Study 1a, we recruited 127 participants through *Prolific Academic*. Participants who failed at least one of two attention checks were excluded from the analyses (*n* = 7). The 120 participants in the final sample were 37.2 ± 13.2 (mean ± std) years old and 50% of them identified as female.

**Figure 1:**
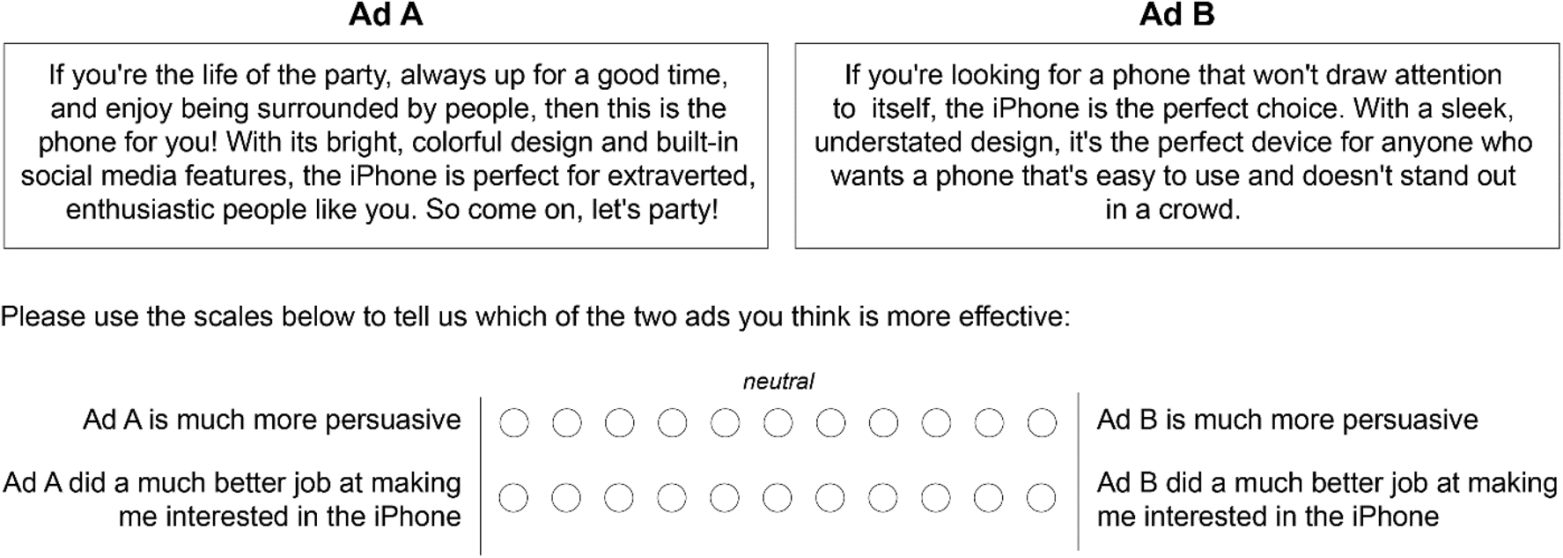
Extraverted and introverted ads for an iPhone generated by GPT-3 alongside the response scale used to record effectiveness ratings.

Participants first indicated their preferences for different iPhone ads. The ad messages were tailored to the high and low ends of the Big Five personality traits using the open-source playground version of GPT-3 (version “text-davinci-003”). For example, we prompted GPT-3 to generate an iPhone ad tailored to Extraversion with the prompt: “Write an iPhone ad for someone who is extraverted and enthusiastic”. In contrast, we prompted it to customize a message for Introversion with: “Write an iPhone ad for someone who is reserved and quiet”. The adjectives used in these prompts (e.g., reserved and quiet) were taken from the language used to identify personality traits (e.g., Introversion) in the Ten-Item-Personality-Inventory (TIPI^54^), but were adjusted in a few instances to reflect more positive versions of the same characteristics (e.g., competitive rather than quarrelsome for low Agreeableness). We did not generate messages for the personality trait of Neuroticism as this trait is unique in that messages designed to “match” the low end of the continuum (i.e., emotionally stable messages) would be appealing to people low and high in Neuroticism^55^. Figure 1 shows examples of the messages generated by GPT-3 for the two prompts above (see Table S1 in the SI for all prompts and messages, and Table S2 for the results of a pre-validation study supporting the intended personality affinity of all stimuli).

We measured people’s preferences for various ads using two 11-point bi-polar scales that contrasted the messages tailored towards the high and low ends of the personality trait (Fig. 1). The bi-polar measure minimizes biased evaluations via “response substitution”^56^. That is, while uni-polar measures (e.g., “How much did this change your opinion?”) might simply capture participants’ a priori evaluation or unrelated individual differences, the current measure focuses on participants’ evaluations of the ads’ relative effectiveness. Effectiveness was calculated as the average score across the two bi-polar items assessed for each ad (see Fig. S1 for distribution of outcome variables). In later studies (Studies 3–4), we demonstrate the generalizability of our findings by taking alternative approaches to assess the messages’ effectiveness.

Finally, participants completed an established measure of the Big Five personality traits (BFI-2S^57^), which asks participants to rate their agreement with a set of 30 statements. Responses were recorded on a 7-point scale ranging from 1 = Strongly Disagree to 7 = Strongly Agree. With Cronbach’s alphas ranging from 0.78 to 0.87, the scale reliabilities were found to be good (Openness = 0.82, Conscientiousness = 0.82, Extraversion = 0.83, Agreeableness = 0.78 and Neuroticism = 0.87). Participants also responded to a series of socio-demographic questions, including age, gender, ethnicity, employment status and education.

### Results Study 1a

**Figure 2:**
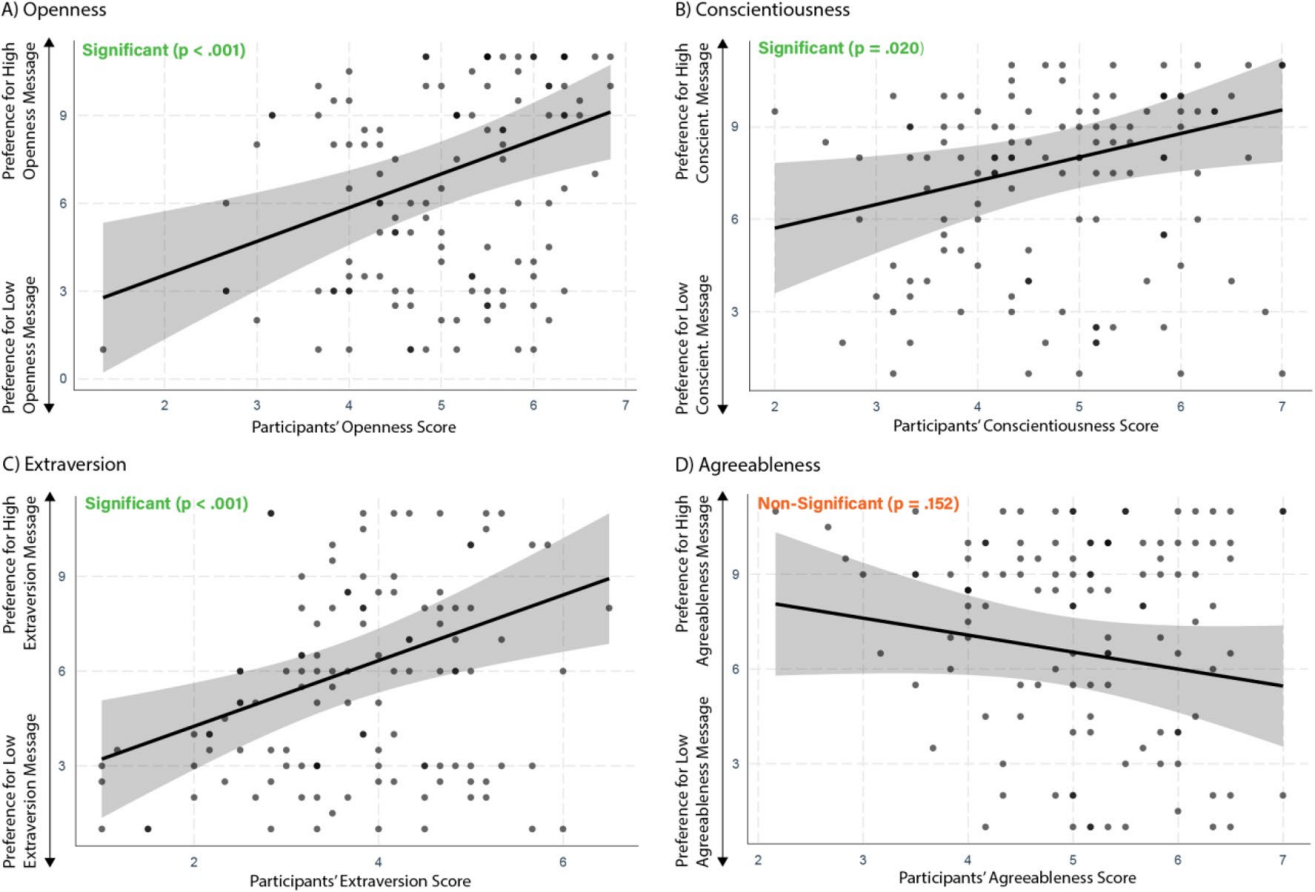
Effects (with 95% confidence intervals) of Big Five personality traits on effectiveness ratings for the respective ads.

To test whether people prefer personalized messages automatically generated by GPT-3, we first ran a series of linear regression analyses, regressing the continuous message effectiveness ratings for each trait on all the Big Five traits and controls (i.e., age, gender, employment status, education and ethnicity; see Table S3 in the Supplementary Information for zero-order correlations). Figure 2 shows the standardized effects with 95% confidence intervals for each of the ad pairs associated with the four personality traits (see Table S4 in the Supplementary Information for full model outputs). Supporting our hypothesis, we found that participants’ Openness (*β* = 0.36, CI_95_ 0.16–0.56, *p* < 0.001), Conscientiousness (*β* = 0.29, CI_95_ 0.05–0.53, *p* = 0.020) and Extraversion (*β* = 0.40, CI_95_ 0.16–0.63, *p* < 0.001) predicted their preferences for the ads tailored to these traits. We did not observe a significant effect for Agreeableness (*β* = -0.17, CI_95_ − 0.40 to 0.06, *p* = 0.152).

### Methods Study 1b

In Study 1b, we recruited a total of 500 participants through *Prolific Academic*. Participants who failed at least one of two attention checks were excluded from the analyses (*n* = 29). The 471 participants in the final sample were 36.2 ± 12.5 (mean ± std) years old and 48% of them identified as female.

All materials and outcome measures were the same as in Study 1a (see Fig. S2 for distribution of outcome variables). Unlike Study 1a, however, participants were randomly assigned to one of three experimental conditions. The first condition (“baseline”) was similar to that of Study 1a. In the second condition (“Disclosure 1”), participants were informed that the ads were generated by GPT-3, a generative AI (*“The ads have been generated by GPT-3, an artificial intelligence program that can use different prompts (e.g. "Please write me an iPhone ad") to create content”)*. In the third condition (“Disclosure 2”), participants were told that GPT-3 had been instructed to create ads tailored to different personality traits (“*The ads have been generated by GPT-3, an artificial intelligence program that can use different prompts (e.g., "Please write me an iPhone ad"*)* to create content. We asked GPT-3 to generate ads tailored to different personalities (e.g., people who are outgoing and social or people who are reserved and quiet”).*

As in Study 1a, participants completed the BFI-2-S to report on their Big Five personality. The scale reliabilities for the BFI-2-S Big Five personality measure^57^ were found to be good to excellent (Openness = 0.85, Conscientiousness = 0.84, Extraversion = 0.83, Agreeableness = 0.79 and Neuroticism = 0.90).

### Results Study 1b

We replicated our earlier findings using the full sample in Study 1b, finding that participants’ Openness (*β* = 0.16, CI_95_ 0.06–0.25, *p* < 0.001), Conscientiousness (*β* = 0.20, CI_95_ 0.09–0.31, *p* < 0.001) and Extraversion (*β* = 0.29, CI_95_ 0.18–0.39, *p* < 0.001), but not Agreeableness (*β* = 0.00, CI_95_ -0.11–0.14, *p* = 0.847) predicted people’s preferences for generative AI ads tailored to these traits (see Table S5 for zero-order correlations and Table S6 for full regression outputs).

To test the impact of our experimental manipulation in Study 1b (i.e., the different disclosures), we ran the same four linear regression analyses while adding an interaction term between the relevant personality trait and the condition. None of the interaction terms were significant, suggesting that the personality matching effects did not vary across experimental conditions. That is, the effects largely persisted despite informing people about the fact that the messages were generated by an AI rather than a human, and that the ads were designed to appeal to different personality traits (see Fig. S3 for a visual depiction of the findings, Table S7 for the full regression outputs and Tables S8–S10 for regression analyses conducted separately for each condition).

## Study 2

Study 2 tested the generalizability of the effects observed in Study 1 by replicating them using a broader set of stimuli and psychological characteristics. Specifically, we used ChatGPT to generate: (1) ads for Nike sneakers, tailored to the Big Five traits, (2) persuasive messages promoting participants to exercise more, tailored to regulatory focus^58^ and (3) political appeals for climate action, tailored to moral foundations. The two new psychological characteristics included in Study 2 were chosen based on their relevance to their respective message domains and prior matching research. *Regulatory focus* captures individual differences in people’s dispositional motivation to pursue their goals by focusing on the attainment of desired outcomes (i.e., promotion focus) or the prevention of undesirable outcomes (i.e., prevention focus, e.g.,^59^). Matching messages to people’s dominant regulatory focus has previously been shown to enhance the effectiveness of personalized persuasion, especially in the health domain^6,60^. *Moral foundations* describe individual differences in people’s moral reasoning (i.e., the way they decide what is right and wrong) along five dimensions: Loyalty, Care, Fairness, Purity and Authority^61^. Research on moral reframing has shown that persuasive political appeals are more effective when they are tailored to people’s moral foundations, or when they are matched with the foundations that closely align with their political ideologies^10,62,63^.

### Methods

We recruited a total of 200 participants through *Prolific Academic*. Participants who failed at least one of three attention checks were excluded from the analyses (*n* = 8). The 192 participants in the final sample were 36.7 ± 13.0 (mean ± std) years old and 49% of them identified as female.

Participants were shown a series of AI-generated persuasive messages about the various topics, which were tailored to the respective psychological profile: Nike sneakers (Big Five traits), exercise (regulatory focus), and climate action (moral foundations). As in Study 1, the prompts for these messages were minimal (e.g., Prevention focus: “Write a short persuasive ad to convince a prevention-focused person to exercise more.”; Fairness: “Write a short political ad in favor of taking climate action that is tailored to people scoring high on the moral foundation of fairness”). All persuasive messages were generated using the most recent openly available version of ChatGPT 3.5 Turbo (compiled on February 16, 2023). Table 1 shows two political speeches tailored to the moral foundations of Fairness and Loyalty (see Tables S11–13 in the Supplementary Information for all prompts and messages, and Tables S14–16 for the results of a pre-validation study supporting the intended psychological affinity of all stimuli).

**Table 1:** Political speeches advocating for climate action generated by ChatGPT to appeal to the moral foundations of fairness and loyalty.

Loyalty	Fairness
Do you believe in standing up for the people and places that you love? Do you feel a strong sense of loyalty to your community, your country, and the natural world around you? If so, then I urge you to consider the benefits of taking action on climate change Climate change is not just an environmental issue—it's a threat to the places and people that we hold dear. From the rising sea levels that threaten our coastal communities to the extreme weather events that disrupt our lives and livelihoods, the impacts of climate change are being felt all around us By taking action on climate change, we can help to protect the people and places that we love, showing our loyalty to our communities and our country. We can invest in resilient infrastructure, prepare for the impacts of climate change, and work to build a stronger and more secure future for all Moreover, taking action on climate change is not just a matter of protecting our own interests—it's also an opportunity to lead by example and inspire others to do the same. By showing our loyalty to the natural world and taking steps to protect it, we can help to create a brighter and more hopeful future for all So if you believe in standing up for the people and places that you love, we urge you to support action on climate change. Together, we can create a future that is both loyal and resilient, reflecting the best of our shared values and commitments to the people and places that we call home	Do you believe in creating a fair and just world for all? Do you feel a strong sense of responsibility to ensure that everyone has access to clean air, clean water, and a healthy environment? If so, then I urge you to consider the benefits of taking action on climate change Climate change is not just an environmental issue—it's a matter of fairness and justice. The impacts of climate change are being felt most acutely by the most vulnerable members of society, including low-income communities and people of color. These communities often lack the resources and political power to protect themselves from the effects of climate change By taking action on climate change, we can help to create a fairer and more just world for all. We can invest in renewable energy sources, promote sustainable transportation, and work to create a more equitable and inclusive society that prioritizes the needs of all people Moreover, taking action on climate change is not just a matter of fairness—it's also an opportunity to lead by example and inspire others to do the same. By showing our commitment to fairness and justice, we can help to create a brighter and more hopeful future for all So if you believe in creating a fair and just world for all, we urge you to support action on climate change. Together, we can create a future that is both sustainable and equitable, reflecting the best of our shared values and commitments to fairness and justice for all

To assess message effectiveness, for each of the Big Five traits and regulatory focus messages, participants used the same bi-polar response scale as in Study 1. For moral foundations, we used an alternative measure that required participants to make trade-offs by allocating a total of 100 points across all messages (prompt: *“Imagine you hear five politicians advocating for climate action. They all have different arguments for why they believe we should act. Please read through all of the arguments carefully and decide how persuasive you find them. You have a total of 100 points to allocate across the five arguments. You can do so by typing the number of points in the box next to each argument. The more persuasive you find an argument, the more points you should allocate to it”*). The trade-offs were used since there are no high and low ends to contrast the foundation dimensions. As with the bi-polar scales, this approach allowed us to assess message effectiveness in a way that removed individual differences and a priori evaluations in people’s general support for the topic, testing liking for the messages themselves. Fig. S4 in the Supplementary Information shows the response distributions for all persuasive messages.

After rating each of the ads, participants completed a series of self-report surveys. We again measured participants’ Big Five personality traits using the 30-item BFI-2-S^57^. With Cronbach’s alphas ranging from 0.78 to 0.87, the scale reliabilities were found to be good (Openness = 0.82, Conscientiousness = 0.82, Extraversion = 0.83, Agreeableness = 0.78 and Neuroticism = 0.87).

We measured regulatory focus (promotion versus prevention) using an adapted measure of the original 18-item scale^64^. Specifically, we removed four items that referred to academic performance, leaving us with 14 items in total, seven each for promotion and prevention-focus (e.g., “*In general, I am focused on preventing negative events in my life*” for prevention and “*I frequently imagine how I will achieve my hopes and aspirations*” for promotion). Responses were recorded on a 7-point scale ranging from 1 = Strongly Disagree to 7 = Strongly Agree. With a Cronbach’s alpha of 0.81 for prevention-focus and 0.90 for promotion-focus, the scale reliabilities of the adapted measure were found to be good to excellent*,* with both measures being uncorrelated (*r* = 0.086, *p* = 0.234). Given that the outcome measure required participants to rate the relative effectiveness of between the promotion and prevention focused message, we used the difference between participants’ dispositional promotion and preventions scores as our predictor.

We measured the moral foundations using the Moral Foundations Questionnaire (MFQ-30^65^), which uses 30 items to measure the five moral foundations: Purity, Care, Loyalty, Fairness and Authority. One set of questions asked participants to indicate the extent to which a certain criterion is relevant to them when deciding whether something is right or wrong (e.g., “*Whether or not someone showed a lack of respect for authority*” for Authority). Responses were recorded on a 6-point scale ranging from 1 = Not at all relevant to 6 = Extremely relevant. The second set of questions asked people to rate their agreement with a series of statements (e.g., “*Justice is the most important requirement for a society*” for Fairness). Responses were recorded on a 6-point scale ranging from 1 = Strongly disagree to 6 = Strongly agree. Scores were averaged across both sets of questions. With Cronbach’s alphas ranging from 0.60 to 0.86, the scale reliabilities were found to be acceptable to good (Purity = 0.77, Care = 0.73, Loyalty = 0.70, Fairness = 0.60 and Authority = 0.86). We also asked participants to report their political ideology on a scale ranging from 1 = Very conservative to 7 = Very liberal.

### Results

We ran a series of linear regression analyses, regressing the continuous message effectiveness ratings for each outcome on the respective set of psychological characteristics and controls. While we added all the Big Five traits into the model simultaneously (similar to Study 1), the moral foundations were added individually due to their relatively high inter-correlations (average *r* = 0.34, max *r* = 0.75). Given that the moral foundations have previously been related to political ideology, and political ideology can be more easily imputed in the real world from online behavioral data or accessed through data brokers, we also tested the impact of political ideology on people’s effectiveness ratings for the moral foundation messages. For this purpose, we totaled the points allocated to the two messages tailored to the dimensions typically associated with a more liberal ideology (Care and Fairness) and regressed that measure on participants’ political ideology and controls.

For the sneaker ads tailored to the Big Five traits, we replicated the findings of Study 1 in that participants’ Openness (*β* = 0.19, CI_95_ 0.04–0.34, *p* = 0.012) and Extraversion (*β* = 0.19, CI_95_ 0.01–0.37, *p* = 0.040) predicted their preferences for the ads tailored to these traits. We did not find any significant effects for Conscientiousness (*β* = 0.08, CI_95_ − 0.12 to 0.28, *p* = 0.424) or Agreeableness (*β* = -0.00 CI_95_ − 0.18 to 0.17, *p* = 0.984; see Fig. S5 for a visualization of marginal effects, Table S17 for zero-order correlations and Table S18 for full regression outputs).

For the health behavior messages tailored to regulatory focus, we found small, but non-significant, matching effects (*β* = 0.12, CI_95_ − 0.03 to 0.27, *p* = 0.125; see Fig. S6 for a visualization of marginal effects, Table S19 for zero-order correlations and Table S20 for full regression outputs).

**Figure 3:**
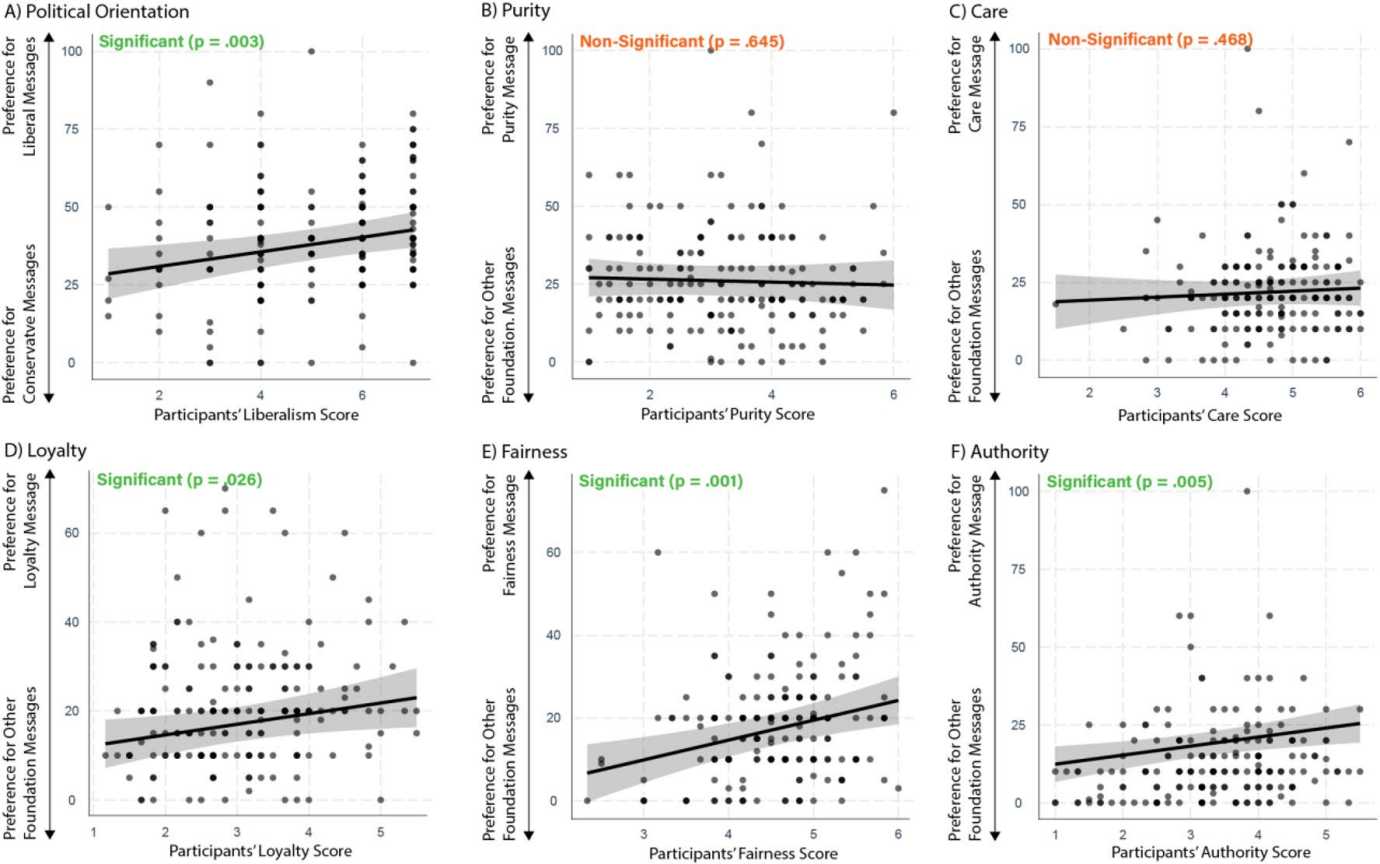
Effects (with 95% confidence intervals) of political ideology and moral foundations on effectiveness ratings for the respective ads.

For the climate change appeals tailored to moral foundations, we found that three of the moral foundations as well as political ideology showed the expected matching effects (Fig. 3; see Table S21 for zero-order correlations). Specifically, we found that participants’ Loyalty (*β* = 0.17, CI_95_ 0.02–0.32, *p* = 0.026), Fairness (*β* = 0.25, CI_95_ 0.11–0.40, *p* = 0.001), Authority (*β* = 0.22, CI_95_ 0.07–0.37, *p* = 0.005) and political orientation (*β* = 0.22, CI_95_ 0.08–0.37, *p* = 0.003) predicted their preferences for the matching ads (Table S22). We did not find any significant effects for Purity (*β* = − 0.04, CI_95_ − 0.20 to 0.12, *p* = 0.645) or Care (*β* = − 0.06, CI_95_ − 0.10 to 0.21, *p* = 0.468).

## Studies 3a–c

Studies 3a and 3b tested the potential of AI-generated matching effects under more conservative conditions to better support our findings’ real-world applicability. This included: (1) replacing the bi-polar response scales with single message evaluations (akin to users scrolling down their newsfeed and evaluating one ad at a time), and (2) gauging message effectiveness on people’s willingness-to-pay (WTP; i.e., “How much would you be willing to spend on X?”) as a proxy for behavior. Previous research has found that self-reported WTP is both an interpretable outcome^66^ and a reflection of people’s actual, in-market demand and pricing decisions^67^.

Study 3c complements Studies 3a-b with an experimental between-subjects design in which participants only evaluated one message at a time (instead of a within-subjects design in which participants evaluated all messages). By using this approach, we offer an even more conservative test of the effects and rule out any remaining artifactual explanations for the findings (e.g., within-stimuli influence from contrasting opposing ads). This study was pre-registered on *AsPredicted.com* (link: aspredicted.org/8ZH_T9L).

### Methods Study 3a

In Study 3a, we focused once again on smartphone advertisements tailored to Big Five personality traits. We recruited 200 participants through *Prolific Academic*. Participants who failed at least one of two attention checks were excluded from the analyses (*n* = 8). The 192 participants in the final sample were 35.7 ± 13.4 (mean ± std) years old and 50% of them identified as female.

We used the four iPhone ads that represented the high ends of the personality traits from Study 1 (i.e., high Openness, high Conscientiousness, high Extraversion and high Agreeableness). Although our analyses were focused on Openness and Extraversion (given that these were the only two traits that showed robust effects in Studies 1 and 2), we retained the other two messages for Agreeableness and Conscientiousness. This was done to statistically account for variance in our outcome measures attributable to individual differences unrelated to people’s ad preferences (e.g., extraverts potentially giving higher scores on rating scales, or individual variation in the amount of money they can afford to spend when purchasing a smartphone). Specifically, we calculated the residuals for each outcome measure by regressing the targeted outcome (e.g., WTP for the phone advertised with the Openness ad) on the equivalent outcomes for the other traits (e.g., WTP for the phone advertised with the Conscientiousness, Extraversion and Agreeableness ads; see^43^ for a similar approach). This allows us to isolate the unique variance in a participant’s preference that is unique to each specific ad (as opposed to the variance that is shared among all of them).

Participants were presented with one ad at a time and indicated their agreement with the following two statements: “This is a persuasive ad” and “This ad has made me more interested in the iPhone” (1 = Strongly Disagree to 7 = Strongly Agree). Effectiveness was calculated as the average of the two items. Participants were also asked to indicate the amount of money in $USD they would be willing to spend on the iPhone with values ranging from $1 to $1000 (WTP; see Fig. S7 for distribution of outcome variables). This range was selected to realistically represent the price of the most advanced iPhone model at the time of data collection ($1000; iPhone 14 Pro) as well as various other prices for older, used or discounted iPhones.

In addition to the control variables used across Studies 1–2, we calculated the average effectiveness/WTP for each participant to further control for differences in averages on these ratings. We also included the position in which an ad was displayed to control for order effects.

Similar to Studies 1 and 2, we measured participants’ Big Five personality traits using the 30-item BFI-2-S^57^. With Cronbach’s alphas ranging from 0.81 to 0.89, the scale reliabilities were found to be good (Openness = 0.81, Conscientiousness = 0.89, Extraversion = 0.82, Agreeableness = 0.82 and Neuroticism = 0.86).

### Results Study 3a

**Figure 4:**
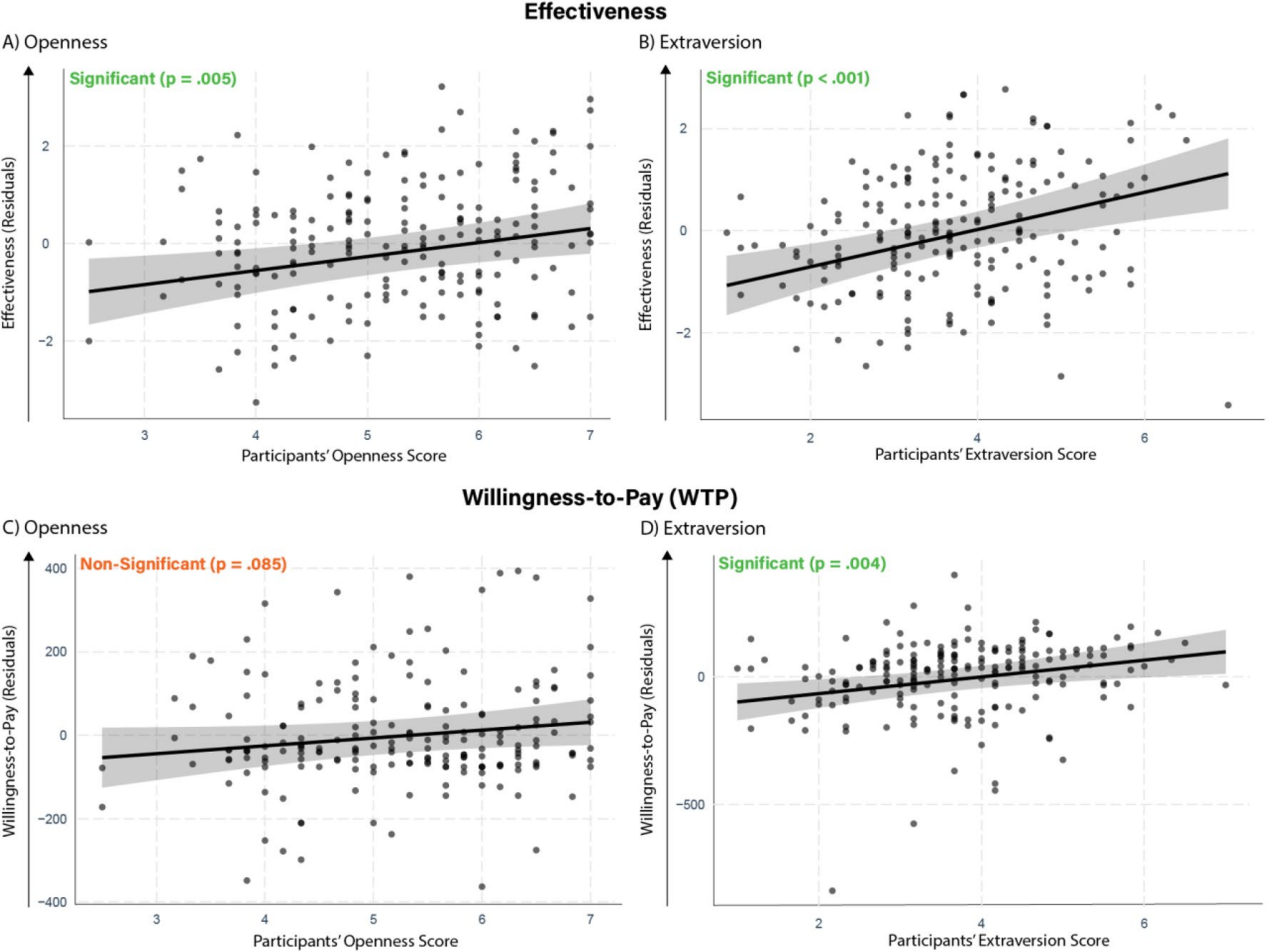
Effects (with 95% confidence intervals) of Big Five personality traits on effectiveness ratings and WTP for the respective ads.

We ran a series of linear regression analyses, regressing the residual effectiveness and WTP ratings for the AI-generated ads on the respective set of psychological characteristics and controls (including the order in which the ad appeared and the average ratings across all ads; Fig. 4).

Replicating the findings from Studies 1 and 2, we found that participants assigned higher effectiveness scores to messages that aligned with their Openness (*β* = 0.24, CI_95_ 0.07–0.41, *p* = 0.005) and Extraversion (*β* = 0.35, CI_95_ 0.18–0.52, *p* < 0.001). Similarly, we found that participants were willing to pay more for the iPhone when the message aligned with their Extraversion scores (*β* = 0.26, CI_95_ 0.09–0.44, *p* = 0.004). An increase of one standard deviation in participant’s extraversion was akin to an increase of $33 in the willingness to pay for the iPhone advertised with the extraverted message. The effect of Openness was found to be marginally significant (*β* = 0.15, CI_95_ − 0.02 to 0.33, *p* = 0.085, equivalent to an increase of $19 in willingness to pay; see Table S23 for zero-order correlations and Tables S24–25 for full regression outputs).

### Methods Study 3b

In Study 3b, we aimed to replicate the effects of AI generated matching on WTP using a different persuasion domain and different set of psychological traits. For this purpose, we recruited 203 participants through *Prolific Academic*. Participants who failed at least one of two attention checks were excluded from the analyses (n = 7). The 196 participants in the final sample were 39.7 ± 14.59 (mean ± std) years old and 48% of them identified as female.

Participants read the five speeches advocating for climate action created for Study 2, one speech at a time, and indicated their agreement with the following two statements: “This is a compelling argument for climate action” and “The argument has made me more interested in supporting climate action”. Responses were recorded on a 7-point scale ranging from 1 = Strongly Disagree to 7 = Strongly Agree. Persuasiveness was calculated as the mean across the two items. Participants were also asked to indicate the amount of money they would be willing to donate to the depicted politician’s campaign with values ranging from $1 to $100 (see Fig. S8 for distribution of outcome variables). This range was selected based on interpretability as well as survey data showing that the average American donates less than $100 to political causes^68^.

Given that liberals are generally more likely to consider climate-change affirming messaging effective and are more likely to donate to climate related causes, we calculated the outcome measure as a difference score. That is, we calculated the difference between the average scores of the liberal-leaning messages (Care and Fairness) and the average scores of the conservative-leaning messages (Purity, Loyalty and Authority).

As in Study 3a, we calculated the average effectiveness/WTP for each participant to further control for differences in average effectiveness and WTP ratings and included the position in which a particular speech was displayed to control for order effects. Participants reported their political ideology on a scale ranging from 1 = Very conservative to 7 = Very liberal.

### Results Study 3b

Replicating the findings from Study 3a, we found that participants assign higher persuasiveness scores to AI-generated messages that align with their political ideology (*β* = 0.18, CI_95_ 0.03–0.33, *p* = 0.018) and are willing to donate more to the politicians that use these messages (*β* = 0.24, CI_95_ 0.09–0.38, *p* = 0.002). An increase of one standard deviation in participant’s liberalism was akin to an additional $2 (out of $100) donated to the politician using matching (liberal compared to conservative) messages (see Fig. S9 for a visualization of marginal effects, Table S26 for zero-order correlations, and Table S27 for full regression outputs). Additional analyses of the residualized effectiveness and WTP scores for each message show that the effects were largely driven by the Fairness and Loyalty messages (Tables S28–29 in the Supplementary Information).

### Methods Study 3c

We recruited 350 participants through *Prolific Academic*, excluding those who failed at least one of two attention checks (*n* = 7). From those remaining, we selected the first 320 responses to adhere to our preregistered target sample (the results remain unchanged when using all 343 participants). The 320 participants in the final sample were 36.1 ± 12.3 (mean ± std) years old and 52% identified as female.

We used two sets of messages: ads that were tailored to high and low levels of Extraversion for the iPhone (see stimuli from Studies 1 and 3a, Table S1), and speeches tailored to Fairness and Loyalty for the political messages on climate action (see Stimuli from Study 2, Table S12). Each participant responded to both scenarios (iPhone and political) but saw and rated only one of the respective messages in each scenario (e.g., either the introverted or extraverted iPhone ad). This design further helped simulate ecologically realistic conditions (i.e., people exposed to ads for different topics), removed demand effects (i.e., by only soliciting evaluations for one of the ads), and eliminated any influence of contrast effects from mismatched messages (i.e., as matching/mismatching was done between-participants).

As preregistered, we collected the same measures as Study 3a (ad effectiveness and willingness to pay; see Fig. S10 for distributions of outcome variables). As before, we measured participants’ Big Five personality traits using the 30-item BFI-2-S^57^. With Cronbach’s alphas ranging from 0.77 to 0.88, the scale reliabilities were found to be good (Openness = 0.82, Conscientiousness = 0.87, Extraversion = 0.84, Agreeableness = 0.77 and Neuroticism = 0.88). Participants also reported their political ideology on a scale ranging from 1 = Very conservative to 7 = Very liberal.

### Results 3c

We ran a series of linear regression analyses, regressing the effectiveness ratings and WTP for each persuasion scenario on the interaction between the type of AI-generated message (specific personality or moral foundation) and the psychological profile of the participant (personality or political ideology). Because responses to our political ideology measure were negatively skewed, we could not dichotomize this variable as preregistered (i.e., any split would have led to an arbitrary distinction or highly uneven groups). Thus, we analyzed the interactions with the continuous Extraversion and political ideology measures (which we had described as an additional robustness check in the preregistration). Importantly, the findings for Extraversion—which allowed for a meaningful median split—remain unchanged when using the dichotomized version.

**Figure 5:**
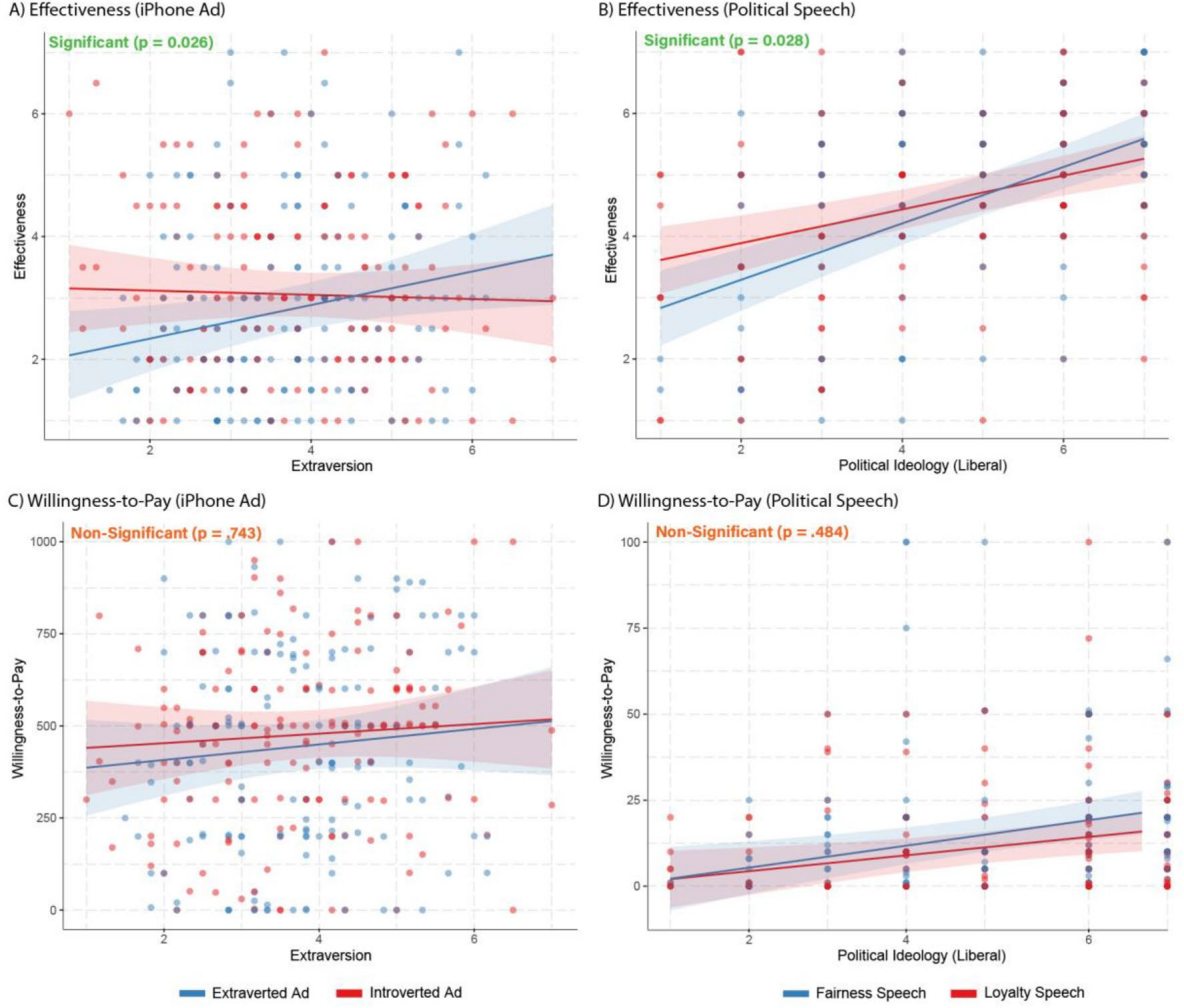
Interaction effects (with 95% confidence intervals) of participants and message personality/political ideology on effectiveness ratings and WTP for iPhone ads and political speeches.

The results of this study replicate our earlier matching effects for the ad effectiveness measure. The AI-generated matched messages were perceived to be more effective in both the iPhone scenario (*β* = 0.25, CI_95_ 0.03–0.47, *p* = 0.026) and the political speech scenario (*β* = 0.23, CI_95_ 0.02–0.43, *p* = 0.028; Fig. 5, see also Table S30 for zero-order correlations, and Table S31 for full regression outputs). Although the effects for the WTP measure were directionally consistent, they were not significant (iPhone: *β* = 0.04, CI_95_ − 0.19 to 0.27, *p* = 0.743, political speech: *β* = 0.08, CI_95_ − 0.14 to 0.30, *p* = 0.484; Fig. 5 and Table S31 for full regression outputs). Instead, we found strong main effects of Extraversion and political ideology on WTP, with extraverts being willing to spend more on the iPhone in general, and liberals willing to donate more to political candidates advocating for climate action. Although the matching effects were weaker for the WTP measure, this is a finding consistent with behavioral phenomena more broadly^69^ and one we discuss in greater detail in “ Discussion”.

## Study 4

In our final study, we more fully simulate the process involved in scaling the use of ChatGPT for personalized persuasion in the real world. That is, instead of designing a limited set of messages ahead of time and assessing participants’ psychological traits post hoc, we recruited participants whose personality profile was known to us prior to the study (i.e., from participating in our earlier studies) and prompted ChatGPT to dynamically create a personalized ad for each individual participant. By subsequently comparing the effectiveness of these personalized ads to that of ads created based on generic prompts, we offer additional evidence for the effectiveness of personalized persuasion using generative AI. The study focuses on two different consumer products, one experiential and one material: a weekend getaway to Rome and Nike sneakers.

### Methods

Approximately 6 to 9 months after data collection for Studies 3a–c, we invited all participants to this new survey on *Prolific Academic*, ending data collection after a requested 300 responses. In total, we received 303 participants, retaining 297 who passed both attention checks (43.4 ± 14.1 (mean ± std) years old and 48% identified as female). We invited these participants specifically, because they completed the Big Five personality test (i.e., the 30-item BFI-2-S)^57^ in our earlier experiments. This allowed us to extract their personality profiles ahead of the current study to prompt ChatGPT to create personality-tailored ads unique to each participant.

The overall procedure for this study was as follows. First, we calculated the percentile scores for Openness, Extraversion and Conscientiousness for each participant (based on the means and standard deviations of all participants from Studies 1–3). We again did not consider Neuroticism (i.e., due to its unique theoretical nature), and likewise, did not include Agreeableness as our previous studies suggested no significant matching-effects for this trait (see “Discussion”).

Second, we identified the most salient personality trait for each participant, defined as the trait whose percentile score deviated the most strongly from the 50% median (in absolute terms). For example, a participant with percentile scores of 40% for Openness, 70% for Extraversion and 15% for Conscientiousness would have been assigned a salient personality of “Low Conscientiousness” (absolute difference to average: |50% − 15%|= 35%). We chose this procedure of identifying a single salient trait over one that would have provided ChatGPT with all the information about a person’s profile to keep the ads focused and concise. This decision was based on pre-testing different options and realizing that a more comprehensive personality profile resulted in ChatGPT using this information in an additive rather than integrative way (see Discussion for a more detailed elaboration and potential future directions).

Third, we used each person’s most salient personality trait to create an ad tailored to this particular trait via OpenAI’s ChatGPT API (version 3.5 Turbo). To simplify the task, we categorized the percentile score into low (≤ 33%), medium(> 33% and < 66%) and high (≥ 66%). Given that we specifically selected the traits based on their level of extremeness (i.e., deviation from 50%), all but 10 out of the 297 participants were described with either a low or a high score of Openness, Extraversion or Conscientiousness (see Table S32 for the relative frequencies of each trait).

To explore the different capabilities of ChatGPT in interpreting our personalized prompts, we followed two different approaches. For the getaway to Rome, we simply supplied ChatGPT with the label of the personality trait (e.g., “*Write a short ad for a weekend getaway to Rome that is tailored to a person who scores low on the Big Five personality trait of Conscientiousness. Do not explicitly mention personality traits in the ad*.”). For the Nike sneakers, we translated the personality trait’s labels as we did in the previous studies (e.g., “*Write a short ad for a Nike Sneaker that is tailored to a person who is disorganized and careless. Do not explicitly mention these personality traits in the ad.*”). Table 2 displays example ads for the weekend getaway. To test the effectiveness of this personalization approach, we also generated five generic ads for each product (prompts: “Write a short ad for a weekend getaway to Rome” and “Write a short ad for a Nike Sneaker”) and randomly assigned one of the outputs to each participant (all participant-specific and generic stimuli can be found on the paper’s OSF page: https://osf.io/79wcm/).

**Table 2:** Examples of generic and personalized ads for a weekend getaway to Rome generated by ChatGPT for individual participants.

Targeted trait	Personalized Ad
Generic	Escape to the enchanting city of Rome for an unforgettable weekend getaway. Discover the city's rich history and culture as you stroll through the charming streets, indulge in authentic Italian cuisine at local trattorias and explore breathtaking landmarks such as the Colosseum and Vatican City. Relax in luxury accommodations and let the magic of Rome enchant you. Book your weekend getaway today and experience the allure of this timeless destination. #RomeWeekendGetaway #ExploreItaly #LaDolceVita
High Openness	Experience all the wonders of ancient Rome on a weekend getaway that will excite your imagination and satisfy your curiosity. Wander the vibrant streets, taking in the stunning architecture and vibrant culture that make Rome one of the world's most fascinating destinations. Enjoy world-class cuisine at local restaurants, see the city's renowned art and historical landmarks, and mingle with fascinating locals. With so much to explore and discover, you're sure to leave Rome feeling invigorated, enlightened, and refreshed. Book your excursion today and get ready to experience Rome's unique brand of magic and splendor!
Low Conscientiousness	Looking for an exciting and effortless break from everyday life? Look no further! The vibrant city of Rome is calling your name. With its sumptuous cuisine, stunning architecture, and endless entertainment options, your itinerary is already set. Sleep in and let yourself be guided by the whims of the moment; who cares about schedules and plans? You're all about seizing the day and living in the now. Let yourself get lost in the maze of cobbled streets, explore hidden corners and indulge in some retail therapy. If you're feeling adventurous, why not try your hand at some local language? The city is your oyster, and you are the free-spirited pearl. Come and recharge your batteries with an unforgettable weekend in Rome!
High Extraversion	Ready to explore the city of la dolce vita? Come to Rome for a weekend full of excitement, adventure, and socializing. You'll be surrounded by incredible architecture, world-famous cuisine, and a vibrant nightlife that will keep you on your toes until sunrise. From the Colosseum to the Piazza Navona, there's no shortage of sights to see, but you'll also have plenty of opportunities to get to know the locals and make new friends. Whether you want to hit up a trendy bar or dance the night away at a club, Rome has something for everyone. Don't wait, book your weekend getaway to Rome now and experience the extraverted side of life!

Participants rated both the generic and the personalized ads for both products. While the order of products was held constant (weekend getaway first, sneakers second), the order in which the generic and personalized ads were shown was randomized. This procedure allowed us to use the first rating for each product in a between-subjects analysis, and the comparison between generic and personalized ads within each product in a within-subjects analysis. Similar to Study 3, we collected two outcome measures: ad effectiveness and willingness to pay (WTP; see Fig. S11 for distributions of all outcome variables). As before, ad effectiveness was measured on a 7-point scale using the following two questions: “I find this ad to be persuasive” and “This ad has made me more interested in [visiting Rome| the Nike sneakers].” WTP was measured using a slider scale ranging from USD 0 to 2000 for the weekend getaway and USD 0 to 150 for the Nike sneakers.

After evaluating the ads, participants completed the 30-item BFI-2-S measure of personality^57^, allowing us to compare the scores we obtained from the prior studies (and used to personalize the ads) with the most current measure of personality. With re-test correlations ranging from *r* = 0.85 for Openness and *r* = 0.91 for Extraversion (mean(*r*) = 0.88), the personality profiles used in the analyses appear to be largely robust and valid. Still, the fact that some of the profiles have shifted makes our estimates of personalized persuasion’s effectiveness conservative but also more realistic. That is, in real-world applications, estimates of consumers’ personality from digital traces might contain substantial amounts of prediction error, or could become outdated over time.

### Results

We conducted both between and within-subjects analyses (see Table S33 for zero-order correlations). The between-subjects analyses compared participants’ evaluations of the first ad they rated for each product using linear regression models. In line with our expectations, participants who were shown a personalized weekend getaway ad rated the ad as significantly more effective (B = 0.43, *β* = 0.31, CI_95_ 0.08–0.53, *p* = 0.008) and were willing to spend a significantly larger amount of money on the trip (B = 116.57, *β* = 0.24, CI_95_ 0.01–0.47, *p* = 0.037) than those who were shown the generic version. Specifically, the personalized ads increased people’s willingness to spend by $117. Although the effects for the sneaker product trended in the expected direction, they were found to be non-significant for both rated effectiveness (B = 0.17, *β* = 0.12, CI_95_ − 0.11 to 0.35, *p* = 0.322) and WTP (B = 5.28, *β* = 0.17, CI_95_ − 0.06 to 0.40, *p* = 0.151). All effects remain robust when including the same set of control variables used in the previous studies (i.e., age, gender, ethnicity, education and employment, see Table S34 for detailed model outputs).

For the within-subjects analyses, we ran a series of paired t-tests comparing each participant's evaluations for the generic ad to that of the personalized ad. The results mirror those observed for the between-person analyses. Participants significantly preferred the personalized ads over the generic ones for the weekend getaway (effectiveness: mean difference = 0.24, *t*(296) = 2.73, *d* = 0.16, *p* = 0.007; WTP: mean difference = 58.13, *t*(296) = 3.22, *d* = 0.19, *p* = 0.001). Although the effects trended in the expected direction for the sneakers, they were not found to be statistically significant (effectiveness: mean difference = 0.11, *t*(296) = 1.41, *d* = 0.08, *p* = 0.161; WTP: mean difference = 1.85, *t*(296) = 1.71, *d* = 0.10, *p* = 0.088). We further discuss the discrepancy between the results on these topics—as well as the varying effectiveness of personalized persuasion for different personality traits, topics and measures—in the following discussion.

## Discussion

The present findings offer robust evidence for the viability of LLMs—and ChatGPT in particular—to automatically generate a diverse array of personalized messages that influence people’s attitudes and behavioral intentions. While prior work has established reliable matching effects (e.g.^4^), some authors have argued that various methodological factors have contributed to the strength of these findings^3^. In the present research, we used a series of conservative tests to instantiate and study matching effects (e.g., consumer and political topics, within- and between-subjects designs, different outcome measures and matched vs. generic messages), consistently demonstrating AI’s proficiency at personalized persuasion.

**Figure 6:**
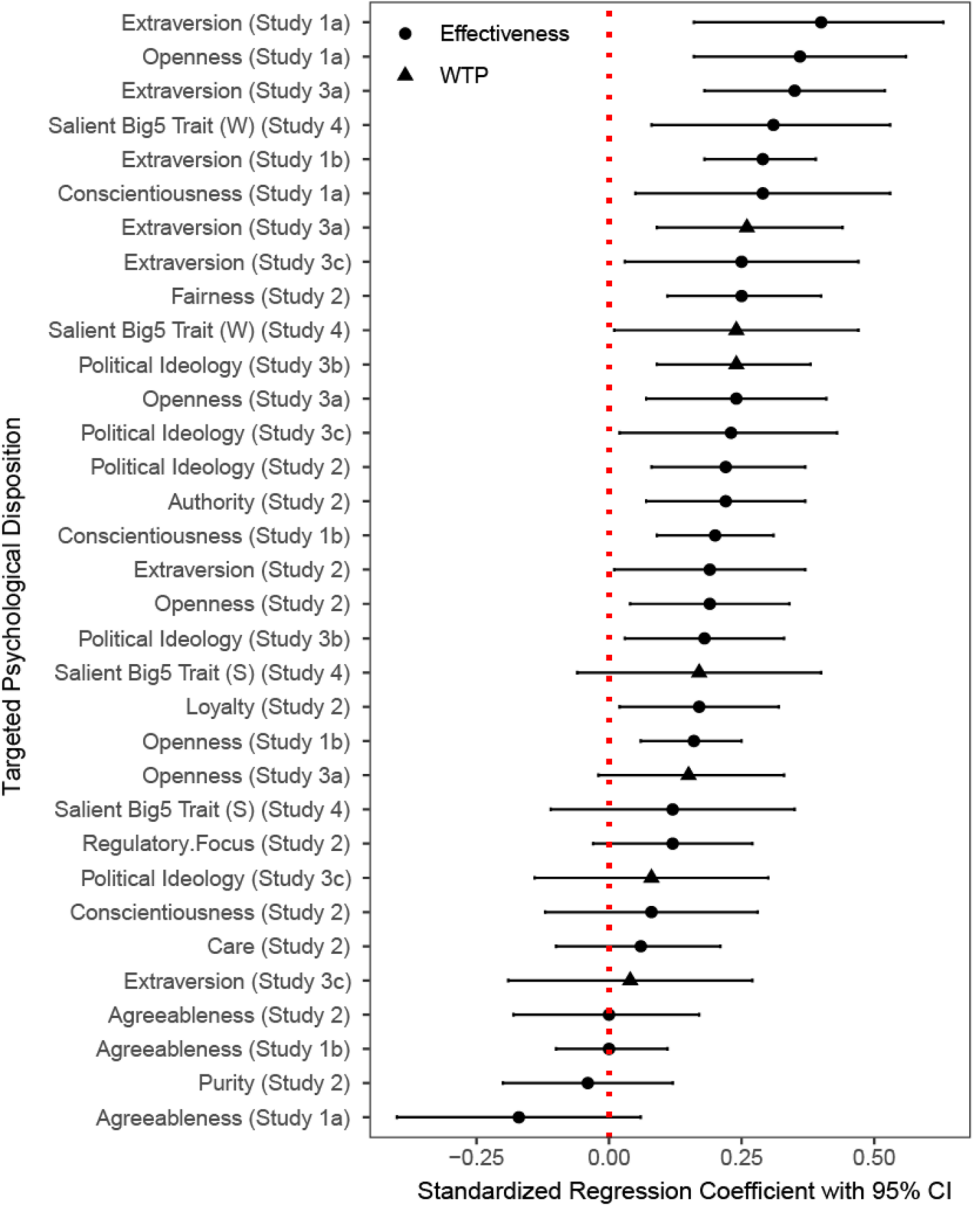
Standardized regression coefficients with 95% confidence interval for the 33 effects tested in this paper (sorted by effect size).

Of the 33 message instantiations we tested, 30 were directionally effective, and 20 were significantly so (61%; Fig. 6). This proportion of significant effects is higher than chance (*t* = 8.30, *p* < 0.001). When extrapolating this effect to the hundreds of advertisements people see daily^70^, the ease with which AI can personalize persuasive message makes their potential influence unprecedented.

Notably, our findings likely represent a relatively conservative estimate of generative AI’s potential to facilitate personalized persuasion at scale. First and foremost, all our studies relied on very short prompts that supplied ChatGPT with a minimal amount of information about the target’s psychological profile as well as the meaning of the specific personality traits. That is, we only focused on high-level traits (e.g., Extraversion) rather than more nuanced personality facets or more granular descriptions of a person’s moral beliefs. In addition, we only prompted ChatGPT with simple sentences that merely named the psychological trait to be targeted (e.g., “Write a short ad for a person who scores high on Extraversion”) or offered a very brief description of the trait (e.g., “Write an ad for someone who is extraverted and enthusiastic.”). While such an approach is likely to mimic many real-world instances where information about targets is scant, the effectiveness of personalized persuasion using LLMs could likely be boosted by offering more detailed insights about the target. Additionally, taking into consideration the rapid advancements in LLMs (e.g., the shift from GPT-3 to GPT-4 that occurred during the progression of this work) as well as the expansion to other modalities known to play a critical role in persuasion (e.g., visual stimuli), the next few years will likely see the continuously growing effectiveness of generative AI in the context of personalized persuasion.

## Heterogeneity in effect sizes

Our findings support the overall effectiveness of personalized persuasion using ChatGPT. However, effect sizes were not uniformly distributed across psychological dimensions, topics and measures. For example, while some psychological traits produced consistent and relatively pronounced matching effects (e.g., Openness and Extraversion), others failed to produce robust effects or reach statistical levels of significance (e.g., Agreeableness). The consistent effects for Openness and Extraversion might be explained by the fact that they are the two most observable traits among the Big Five^48^. Consequently, it is possible, for example, that generative LLMs have more training data available on these characteristics. Indeed, algorithms are able to more accurately predict Openness and Extraversion from people’s Facebook status updates than Conscientiousness and Agreeableness^71^. In contrast, the consistent null effect that was observed for AI-generated messages matched to participants’ level of Agreeableness might be explained by the association of the trait with a broad susceptibility to persuasion^72^. That is, people who are higher in Agreeableness are more likely to respond to persuasive content than those lower in Agreeableness, regardless of the specific personalization strategy deployed. Future research should establish whether these differences are unique to the LLM-generated content, or whether theoretical factors do indeed underlie them (i.e., they would emerge in human-generated content, too). For example, some research suggests that Agreeableness is associated with altruism and harmony in social relationships^73^, which none of our AI-generated messages referenced.

In addition to effect size differences between psychological profiles, we also found differences in the effectiveness of personalized persuasion across topics. For example, in Study 4, the matched messages were more effective for the experiential product (a Rome getaway) than they were for the material one (sneakers). These differences might be explained by a number of factors. For example, the experiential nature of the weekend getaway—with the opportunity to highlight different activities and aspects of the trip—might allow for more genuine and meaningful personalization than that for a pair of sneakers. Additionally, prior research has suggested that matching is more effective for expensive products^74^, because people are more concerned about making the right choice in these instances. Naturally, many other differences between our topics could explain our varying effect sizes within our studies; however, these differences might not be unique to AI-generated personalized persuasion and would be broadly worth considering in this area of research.

Finally, our effects tended to be stronger for the self-reported persuasiveness measures than our behavioral intention ones (i.e., willingness to pay). This is consistent with research in the behavioral sciences^69^ which finds that self-report measures are more sensitive to treatment effects than behavioral measures. That is, although people find matched messages generated by AI more compelling, the translation of those effects onto behavioral proxies (WTP) might require a larger sample to detect small effects or repeated exposures to strengthen the effectiveness of the treatment (especially when the manipulation is rather basic as was the case in our study). Without these strengthening factors, behavioral effects might be overshadowed by strong individual differences that dictate people’s general attitudes/preferences toward a topic^75^.

## Practical implications

While prior research has shown that machine learning algorithms can predict a person’s psychological profile from their digital footprints (e.g.^19,20^) the present work showcases how algorithms can now also design messages that appeal to these traits—even when given very limited input. In other words, current technologies, which continue to innovate and improve, have the potential to allow message sources to fully “close the loop” on automating personalized persuasion. In short, one of the most powerful forms of behavioral influence now has the potential to be implemented at scale.

Companies, for example, could set up fully automated processes that leverage AI to execute sophisticated personalized marketing at scale. Using consumer data from various sources (e.g., browsing patterns, the user’s demographics data, public Facebook Likes or Instagram posts) in combination with predictive algorithms, they could first generate detailed profiles of their consumers’ psychological traits. These profiles could then be automatically funneled to generative AI models designed to automatically create persuasive communication (“write an ad for toothpaste *X* that is shown to an extraverted, 33 years old man, who is likely to pay up to $6 for the product”). In addition to personalized textual content, such algorithms could also produce visual content (still or video) or auditory stimuli. The combined marketing message could subsequently be displayed to the consumer in real-time and adjusted dynamically based on the consumer's interaction with the content. As the entire process relies on AI, it could operate at nearly no cost (outside of the development and maintenance) and readily adapt to recipients’ responses and consequent behavior.

It has not escaped our notice that although these technological developments offer the potential for great societal good (i.e., encourage greater engagement in prosocial behaviors, such as vaccinations or voting), they also pose both short- and long-term threats to the wellbeing of individuals and communities^11^. Facebook, for example, announced that they will use AI generated messages by the end of 2023 to design personalized advertisements^76^. While this integration might make content more engaging, it could also lead to users purchasing products and services they do not need or cannot afford, intensify the battle over social issues and exacerbate mental health challenges (i.e., loneliness, addiction).

Beyond consumerism, the use of LLMs for persuasion also raises serious concerns with regards to politics and society. For example, describing a politician’s stance in language that matches a person’s psychological profile (e.g., talking in terms of the moral foundation of Loyalty for those who value it) could lead people to be more positive toward candidates or issues than they would be otherwise (e.g.^63^). Social media platforms have already been accused of situating people in information ecologies that serve as “echo chambers”, only showing them content that reinforces their interests or worldviews^77^. The ability of LLMs to tailor the language of advertisements, news articles or political speeches, creates a dangerous potential to further enmesh people in their own idiosyncratic worlds that are devoid of a shared reality with distinct others^78^.

In light of these potential risks, it is imperative that oversight of this LLM-personalized content is maintained. The implementation of such oversight, however, is complicated by a few factors. First, one of the foremost solutions being considered for combatting AI influence—disclosing whether a message was generated by AI—may be ineffective at curbing their effects. Our findings offer initial evidence that disclosing the source of a persuasive message (i.e., “made by AI”) did not change its persuasive impact. Thus, future research should replicate this finding to offer stronger, more generalizable advice for policy makers. Second, because each piece of AI-generated content is personalized to a particular individual, it will be nearly impossible to recreate a viewer’s journey for auditing purposes. Relatedly, the speed with which LLMs can create this personalized content further challenges individual oversight (i.e., as the influx of content could be too vast to moderate). Consequently, safeguards against the influence of AI-generated messages might have to rely less on whether each advertisement maintains an appropriate level of veracity for the person to which it is shown, and instead, focus on ensuring that the account behind the persuasive appeal (or the online platform who hosts them) meets aggregated veracity standards. For example, academic proposals to regulate generative AI have collectively argued that regulation should occur at the stage of deployment (e.g., at the advertiser and platform level), rather than at the level of message reception (e.g., at the time point consumers are seeing the specific ad)^79^. In the absence of such broader oversight, an arms race may ignite (akin to the one that occurred in the world of computer viruses) where “auditing AI” software will be created to test the content for unlawful levels of intrusiveness, while the major platforms and malevolent players will work to evade the scrutiny of algorithms as they attempt to exert their influence. Future work should investigate how regulation at the back-end of widely available LLMs can prevent the misuse of these technologies in various contexts, such as companies encouraging compulsive buying (e.g.^80^) and deploying manipulative marketing (e.g.^81^).

## Limitations and conclusion

The current research serves as compelling empirical evidence for the effectiveness of LLM-generated personalized persuasion. However, there are a number of important limitations that should be addressed by future research. First, while behavioral intentions and participants’ willingness to pay are predictive of actual behavior^82,83^, the research does not demonstrate their effects outside of self-report measures which are known to be prone to a variety of response biases^84^. Although we used a variety of measurement approaches to circumvent some of these concerns (none of which asked about self-predicted change, which can be problematic^57^), future research would benefit from replicating the current findings using alternative, behavioral outcome measures (e.g., counterfactual formats^85^).

Second, we cannot directly speak to the question of whether our effects are driven by the enhanced persuasiveness of matched messages versus the reduced persuasiveness of mismatched ones^86^. This is a debate within the matching literature more broadly regarding how psychologically-matched messages perform in comparison to “neutral” non-tailored messages. Although Study 4 showed matching effects when comparing personalized messages to generic ones —thereby providing some evidence for the positive utility of matching—future research is needed to replicate this effect and determine the conditions under which this finding is true.

Third, all our studies focused on a single psychological trait rather than a more holistic view of a person’s entire psychological profile (e.g., one Big Five personality trait rather than a combination of all five traits). While this decision was in part driven by the fact that such an approach most closely resembles current applications of personalized persuasion in targeted advertising, future research should explore the utility of different levels of personalization. As we briefly described in “Methods” section of Study 4, we had originally intended to use ChatGPT to dynamically craft messages tailored to people’s holistic profiles (e.g., by prompting ChatGPT to generate an ad tailored to someone who scores high on Openness, low on Extraversion and average on Conscientiousness). However, an inspection of the resulting stimuli revealed that ChatGPT used the insights about people’s personality traits in a somewhat artificial, additive way rather than a more seamless, integrative way. That is, the messages started with a sentence tailored to Openness, followed by a sentence tailored to Extraversion and finally a sentence tailored to Conscientiousness, rather than one integrative message considering the unique needs of a person who is both open-minded and introverted. We encourage future research to investigate how different forms of prompt engineering might make it possible to overcome this current limitation, and test whether doing so could further increase the effectiveness of AI-based personalized persuasion.

Finally, while LLMs can surely speed up and scale the generation of content, it is not clear whether the generated messages outperform those of human authors. While prior work suggests that LLMs could outperform lay people by overcoming common egocentrism biases^47^, they might still underperform compared to professionals with extensive training and experience (e.g. marketing professionals, speech writers). Notably, LLMs are still in their most nascent stage of development, meaning any evidence for their success at present only hints at their potential influence to come. As generative AI becomes increasingly powerful, they could either replace most human experts (e.g., creative individuals and marketing practitioners) or—on a more positive note—empower these experts to expand their skill sets and use AI to elevate their current performance levels.

### Supplementary Information

Supplementary Information.

## References

[1] Parkins, C. The world most valuable resource is no longer oil but data. *The Economist* (2017). https://www.economist.com/leaders/2017/05/06/the-worlds-most-valuable-resource-is-no-longer-oil-but-data.

[2] Bhageshphur, K. Data is the New Oil. *Forbes Magazine* (2019). https://www.forbes.com/sites/forbestechcouncil/2019/11/15/data-is-the-new-oil-and-thats-a-good-thing/?sh=3f380a157304.

[3] Joyal-Desmarais K, Scharmer AK, Madzelan MK, See JV, Rothman AJ, Snyder M (2022). Appealing to motivation to change attitudes, intentions, and behavior: A systematic review and meta-analysis of 702 experimental tests of the effects of motivational message matching on persuasion. Psychol. Bull..

[4] Teeny JD, Siev JJ, Briñol P, Petty RE (2021). A review and conceptual framework for understanding personalized matching effects in persuasion. J. Consum. Psychol..

[5] Matz SC, Kosinski M, Nave G, Stillwell DJ (2017). Psychological targeting as an effective approach to digital mass persuasion. Proc. Natl. Acad. Sci..

[6] Latimer AE, Rivers SE, Rench TA, Katulak NA, Hicks A, Hodorowski JK, Higgins ET, Salovey P (2008). A field experiment testing the utility of regulatory fit messages for promoting physical activity. J. Exp. Soc. Psychol..

[7] Bogg T, Vo PT (2022). Realistic effort action plans (REAP) for exercise among underactive and inactive university students: A randomized trial. J. Am. Coll. Health.

[8] Latimer AE, Katulak NA, Mowad L, Salovey P (2005). Motivating cancer prevention and early detection behaviors using psychologically tailored messages. J. Health Commun..

[9] Matz SC, Gladstone JJ, Farrokhnia RA (2022). Leveraging psychological fit to encourage saving behavior. Am. Psychol..

[10] Feinberg M, Willer R (2013). The moral roots of environmental attitudes. Psychol. Sci..

[11] Matz SC, Appel RE, Kosinski M (2019). Privacy in the age of psychological targeting. Curr. Opin. Psychol..

[12] Lukito J (2020). Coordinating a multi-platform disinformation campaign: Internet Research Agency activity on three US social media platforms, 2015 to 2017. Polit. Commun..

[13] Bailenson JN, Garland P, Iyengar S, Yee N (2006). Transformed facial similarity as a political cue: A preliminary investigation. Polit. Psychol..

[14] Tappin BM, Wittenberg C, Hewitt LB, Berinsky AJ, Rand DG (2023). Quantifying the potential persuasive returns to political microtargeting. Proc. Natl. Acad. Sci..

[15] Jackler, R. K., *et al.*, JUUL Advertising Over its First Three Years on the Market (2019).

[16] Boerman SC, Kruikemeier S, Zuiderveen Borgesius FJ (2017). Online behavioral advertising: A literature review and research agenda. J. Advert..

[17] The White House—Office of Science and Technology Policy, “Blueprint for an AI Bill of Rights: Making Automated Systems Work for The American People” (2022). https://www.whitehouse.gov/ostp/.

[18] Stachl C, Boyd RL, Horstmann KT, Khambatta P, Matz SC, Harari GM (2021). Computational personality assessment. Personal. Sci..

[19] Youyou W, Kosinski M, Stillwell D (2015). Computers judge personalities better than humans. Proc. Natl. Acad. Sci..

[20] Kosinski M, Stillwell D, Graepel T (2013). Private traits and attributes are predictable from digital records of human behavior. Proc. Natl. Acad. Sci. USA.

[21] Park G, Schwartz HA, Eichstaedt J, Kern ML, Kosinski M, Stillwell D, Ungar LH, Seligman MEP (2014). Automatic personality assessment through social media language. J. Pers. Soc. Psychol..

[22] Schwartz HA, Eichstaedt JC, Kern ML, Dziurzynski L, Ramones SM, Agrawal M, Shah A, Kosinski M, Stillwell D, Seligman MEP, Ungar LH (2013). Personality, gender, and age in the language of social media: The open-vocabulary approach. PLoS One.

[23] Cutler A, Condon DM (2022). Deep lexical hypothesis: Identifying personality structure in natural language. J. Pers. Soc. Psychol..

[24] Christian H, Suhartono D, Chowanda A, Zamli KZ (2021). Text based personality prediction from multiple social media data sources using pre-trained language model and model averaging. J. Big Data.

[25] Peters, H., & Matz, S. C. Large language models can infer psychological dispositions of social media users. arXiv:2309.08631 [Preprint] (2023). 10.48550/arXiv.2309.08631. 10.1093/pnasnexus/pgae231 PMC11211928 38948324

[26] Segalin C, Perina A, Cristani M, Vinciarelli A (2017). The pictures we like are our image: Continuous mapping of favorite pictures into self-assessed and attributed personality traits. IEEE Trans. Affect. Comput..

[27] Gladstone JJ, Matz SC, Lemaire A (2019). Can psychological traits be inferred from spending? Evidence from transaction data. Psychol. Sci..

[28] Ramon Y, Farrokhnia RA, Matz SC, Martens D (2021). Explainable AI for psychological profiling from behavioral data: An application to big five personality predictions from financial transaction records. Information.

[29] Stachl, C., et *al.* Behavioral Patterns in Smartphone Usage Predict Big Five Personality Traits (PsyArXiv, 2019).

[30] Liberali G, Hauser JR, Urban GL, Braun M (2009). Website morphing. Mark. Sci..

[31] Touvron, H. *et al.*, Llama 2: Open Foundation and Fine-Tuned Chat Models. arXiv:2307.09288 [Preprint] (2023). 10.48550/arXiv.2307.09288.

[32] Touvron, H., *et al.* LLaMA: Open and Efficient Foundation Language Models. arXiv:2302.13971 [Preprint] (2023). doi:10.48550/arXiv.2302.13971.

[33] Anthropic, “Model Card and Evaluations for Claude Models” (2023).

[34] Anil, R. *et al.*, PaLM 2 Technical Report. arXiv arXiv:2305.10403 [Preprint] (2023). 10.48550/arXiv.2305.10403.

[35] OpenAI, “GPT-4 System Card” (2023).

[36] Vaswani, A., *et al.* Attention Is All You Need. arXiv:1706.03762 [Preprint] (2023). 10.48550/arXiv.1706.03762.

[37] Radford, A., Wu, J., Child, R., Luan, D., Amodei, D., & Sutskever, I. Language Models are Unsupervised Multitask Learners.

[38] Hu, K. ChatGPT sets record for fastest-growing user base—analyst note. *Reuters* (2023). https://www.reuters.com/technology/chatgpt-sets-record-fastest-growing-user-base-analyst-note-2023-02-01/.

[39] Karinshak E, Liu SX, Park JS, Hancock JT (2023). Working with AI to persuade: Examining a large language model’s ability to generate pro-vaccination messages. Proc. ACM Hum.-Comput. Interact..

[40] Bai H, Voelkel JG, Eichstaedt JC, Willer R (2023). Open Sci. Framew..

[41] Graves, C. Generative AI Can Help You Tailor Messaging to Specific Audiences. *Harvard Business Review* (2023). https://hbr.org/2023/02/generative-ai-can-help-you-tailor-messaging-to-specific-audiences.

[42] Marr, B. How Will ChatGPT Affect Your Job If You Work In Advertising And Marketing?. *Forbes Magazine* (2023). https://www.forbes.com/sites/bernardmarr/2023/01/17/how-will-chatgpt-affect-your-job-if-you-work-in-advertising-and-marketing/?sh=6d8e6ed739a3.

[43] Zhang X, Zou Y, Zhang H, Zhou J, Diao S, Chen J, Ding Z, He Z, He X, Xiao Y, Long B, Yu H, Wu L (2022). Automatic product copywriting for E-commerce. Proc. AAAI Conf. Artif. Intell..

[44] Binz M, Schulz E (2023). Using cognitive psychology to understand GPT-3. Proc. Natl. Acad. Sci..

[45] Hagendorff, T., Fabi, S., & Kosinski, M. Machine intuition: Uncovering human-like intuitive decision-making in GPT-3.5. arXiv:2212.05206 [Preprint] (2022). 10.48550/arXiv.2212.05206.

[46] Kosinski, M. Theory of mind may have spontaneously emerged in large language models. arXiv:2302.02083 [Preprint] (2023). 10.48550/arXiv.2302.02083.

[47] Feinberg M, Willer R (2015). From gulf to bridge: When do moral arguments facilitate political influence?. Pers. Soc. Psychol. Bull..

[48] McCrae RR, John OP (1992). An introduction to the five-factor model and its applications. J. Pers..

[49] McCrae RR, Allik IU (2002). The Five-Factor Model of Personality across Cultures.

[50] Hirsh JB, Kang SK, Bodenhausen GV (2012). Personalized persuasion: Tailoring persuasive appeals to recipients’ personality traits. Psychol. Sci..

[51] Matz SC, Kosinski M, Nave G, Stillwell D (2017). Psychological targeting as an effective approach to digital mass persuasion. Proc. Natl. Acad. Sci..

[52] Gerber AS, Huber GA, Doherty D, Dowling CM, Ha SE (2010). Personality and political attitudes: Relationships across issue domains and political contexts. Am. Polit. Sci. Rev..

[53] Goldberg LR, Johnson JA, Eber HW, Hogan R, Ashton MC, Cloninger CR, Gough HG (2006). The international personality item pool and the future of public-domain personality measures. J. Res. Personal..

[54] Gosling SD, Rentfrow PJ, Swann WB (2003). A very brief measure of the Big-Five personality domains. J. Res. Personal..

[55] Matz SC, Jansson-Boyd CV, Zawisza MJ (2017). Personality-customized advertising in the digital environment. Routledge International Handbook of Consumer Psychology.

[56] Graham MH, Coppock A (2021). Asking about attitude change. Public Opin. Q..

[57] Soto CJ, John OP (2017). Short and extra-short forms of the Big Five Inventory-2: The BFI-2-S and BFI-2-XS. J. Res. Personal..

[58] Psychology ES, Review P, Zanna ME (2000). Regulatory focus as a motivational principle. Advances in Experimental Social Psychology, Vol. 30.

[59] Higgins ET, Friedman RS, Harlow RE, Idson LC, Ayduk ON, Taylor A (2001). Achievement orientations from subjective histories of success: Promotion pride versus prevention pride. Eur. J. Soc. Psychol..

[60] Cesario J, Higgins ET, Scholer AA (2008). Regulatory fit and persuasion: Basic principles and remaining questions. Soc. Personal. Psychol. Compass.

[61] Graham J, Haidt J, Koleva S, Motyl M, Iyer R, Wojcik SP, Ditto PH (2013). Moral foundations theory: The pragmatic validity of moral pluralism. Advances in Experimental Social Psychology.

[62] Feinberg M, Willer R (2019). Moral reframing: A technique for effective and persuasive communication across political divides. Soc. Personal. Psychol. Compass.

[63] Voelkel JG, Feinberg M (2018). Morally reframed arguments can affect support for political candidates. Soc. Psychol. Personal. Sci..

[64] Lockwood P, Jordan CH, Kunda Z (2002). Motivation by positive or negative role models: Regulatory focus determines who will best inspire us. J. Pers. Soc. Psychol..

[65] Graham J, Nosek BA, Haidt J, Iyer R, Spassena K, Ditto PH (2008). Moral foundations questionnaire. J. Pers. Soc. Psychol..

[66] Schmidt J, Bijmolt THA (2020). Accurately measuring willingness to pay for consumer goods: A meta-analysis of the hypothetical bias. J. Acad. Mark. Sci..

[67] Miller KM, Hofstetter R, Krohmer H, Zhang ZJ (2011). How should consumers’ willingness to pay be measured? An empirical comparison of state-of-the-art approaches. J. Mark. Res..

[68] Hughes, A. 5 facts about U.S. political donations, *Pew Research Center*. https://www.pewresearch.org/short-reads/2017/05/17/5-facts-about-u-s-political-donations/.

[69] Ajzen I (1991). The theory of planned behavior. Organ. Behav. Hum. Decis. Process..

[70] Anderson, S. How many ads do we really see in a day? Spoiler: It’s not 10,000, *The Drum* (2023). https://www.thedrum.com/news/2023/05/03/how-many-ads-do-we-really-see-day-spoiler-it-s-not-10000.

[71] Park G, Schwartz HA, Eichstaedt JC, Kern ML, Kosinski M, Stillwell DJ, Seligman ME (2015). Automatic personality assessment through social media language. J. Pers. Soc. Psychol..

[72] Alkış N, Taşkaya Temizel T (2015). The impact of individual differences on influence strategies. Personal. Individ. Differ..

[73] Gerber AS, Huber GA, Doherty D, Dowling CM, Panagopoulos C (2013). Big five personality traits and responses to persuasive appeals: Results from voter Turnout experiments. Polit. Behav..

[74] Gladstone JJ, Garbinsky EN, Matz SC (2022). When does psychological fit matter? The moderating role of price on self-brand congruity. Soc. Psychol. Personal. Sci..

[75] Fazio RH, Olson MA (2014). The MODE Model: Attitude-Behavior Processes as a Function of Motivation and Opportunity. In Dual-Process Theories of the Social Mind.

[76] Mehta, I. Meta wants to use generative AI to create ads. *TechCrunch* (2023). https://techcrunch.com/2023/04/05/meta-wants-to-use-generative-ai-to-create-ads/.

[77] Boutyline A, Willer R (2017). The social structure of political echo chambers: Variation in ideological homophily in online networks. Polit. Psychol..

[78] Higgins ET (2019). Shared Reality: What Makes Us Strong and Tears Us Apart.

[79] Hacker, P., Engel, A., & Mauer, M. Regulating ChatGPT and other Large Generative AI Models. In *Proceedings of the 2023 ACM Conference on Fairness, Accountability, and Transparency*, 1112–1123 (Association for Computing Machinery, 2023). 10.1145/3593013.3594067.

[80] O’Guinn TC, Faber RJ (1989). Compulsive buying: A phenomenological exploration. J. Consum. Res..

[81] Aylsworth T (2022). Autonomy and manipulation: Refining the argument against persuasive advertising. J. Bus. Ethics.

[82] Whittington D, Adamowicz W, Lloyd-Smith P (2017). Asking willingness-to-accept questions in stated preference surveys: A review and research agenda. Annu. Rev. Resour. Econ..

[83] Lloyd-Smith P, Adamowicz W (2018). Can stated measures of willingness-to-accept be valid? Evidence from laboratory experiments. J. Environ. Econ. Manag..

[84] Podsakoff PM, MacKenzie SB, Lee J-Y, Podsakoff NP (2003). Common method biases in behavioral research: A critical review of the literature and recommended remedies. J. Appl. Psychol..

[85] Ozer DJ, Benet-Martinez V (2006). Personality and the prediction of consequential outcomes. Annu. Rev. Psychol..

[86] Hersh ED, Schaffner BF (2013). Targeted campaign appeals and the value of ambiguity. J. Polit..

