# Supplementary material for: The potential of generative AI for personalized persuasion at scale
Source: Sci Rep. 2024 Feb 26;14:4692. doi: 10.1038/s41598-024-53755-0 (PMC10897294; doi:10.1038/s41598-024-53755-0)
Supplement: Supplementary file 1 — Supplementary Information. [file 41598_2024_53755_MOESM1_ESM.docx]

**Supplementary Information**

**Study 1a**

**Sample demographics**

| Age | 37.2 (13.2SD) |
| --- | --- |
| Gender | 50% Female |
| Ethnicity | 75% White  9% Black or African American  0% American Indian, Native American or Alaska Native  6% Asian  0% Hawaiian Native or Other Pacific Islander  3% Other  7% Multiracial |
| Education | 2% Some high school or less  12% High school diploma or GED  19% Some college but no degree  11% Associates or technical degree  43% Bachelor’s degree  13% Graduate or professional degree (MA, MS, MBA, PHD, JD, MD etc.) |
| Income | 19% Less than $10,000  6% $10,000 to $14,999  9% $15,000 to $24,999  11% $25,000 to $34,999  11% $35,000 to $49,999  21% $50,000 to $74,999  10% $75,000 to $99,999  9% $100,000 to $149,999  4% $150,000 to $199,999  0% $200,000 or more |
| Employment | 50% Employed (full-time)  15% Employed (part-time)  10% Self-employed  9% Unemployed  8% Student  3% Retired  4% Other |

**Table S1.** GPT-3 prompts and tailored messages for the low and high ends of the four personality traits of Openness (OP), Conscientiousness (CO), Extraversion (EX) and Agreeableness (AG).

| Trait | High | Low |
| --- | --- | --- |
| OP | **Write an iPhone ad for someone who is open to new experience and artistic.**  If you're looking for an iPhone that will help you open up to new experiences and be more artistic, look no further than the iPhone 11 Pro. With its powerful cameras and editing tools, you'll be able to capture and create beautiful images and videos like never before. So whether you're a budding photographer or just someone who appreciates art, the iPhone 11 Pro is the perfect choice for you. | **Write an iPhone ad for someone who is down-to-earth and traditional**  Looking for a phone that won't break the bank and will still get the job done? Look no further than the iPhone! Whether you're checking your email, browsing the web, or playing your favorite games, the iPhone has you covered. Plus, with a traditional design, you'll feel right at home with this phone. |
| CO | **Write an iPhone ad for someone who is dependable and organized.**  If you're looking for someone who is dependable and organized, look no further than the iPhone. With its sleek design and powerful features, the iPhone is perfect for anyone who wants to stay on top of their game. From keeping track of your schedule to managing your finances, the iPhone has everything you need to stay organized and on track. So why wait? Get the iPhone today and start living your best life. | **Write an iPhone ad for someone who is disorganized and careless**  If you're constantly losing your keys, forgetting important dates, and generally feeling disorganized and stressed, our new iPhone app is perfect for you! Stay on top of your life with reminders, alarms, and to-do lists, and never worry about losing track of anything again. |
| EX | **Write an iPhone ad for someone who is extraverted and enthusiastic.**  If you're the life of the party, always up for a good time, and enjoy being surrounded by people, then this is the phone for you! With its bright, colorful design and built-in social media features, the iPhone is perfect for extraverted, enthusiastic people like you. So come on, let's party! | **Write an iPhone ad for someone who is reserved and quiet**  If you're looking for a phone that won't draw attention to itself, the iPhone is the perfect choice. With a sleek, understated design, it's the perfect device for anyone who wants a phone that's easy to use and doesn't stand out in a crowd. |
| AG | **Write an iPhone ad for someone who is sympathetic and warm**  Looking for a phone that will make you feel more connected to the people you care about? Look no further than the iPhone. With its sleek design and powerful features, the iPhone is the perfect way to stay connected to your loved ones. | **Write an iPhone ad for someone who is critical and competitive**  If you're the type of person who is always looking for a challenge and loves to be competitive, then the iPhone is the perfect device for you. With its many features and apps, the iPhone is sure to keep you entertained and engaged. So come on, what are you waiting for? Pick up an iPhone today and see for yourself what all the hype is about. |

**Stimulus validation**

To ensure that the stimuli displayed in Table S1 reflect the personality traits they were meant to target, we ran a small pre-validation study. Specifically, we recruited 20 undergraduate and graduate students to read the messages and indicate which personality trait they believed the message was tailored to:

***“****On the following pages we will show you ads for an****iPhone****and ask you for your reaction. The ads have been designed to appeal to people with different****personality traits****. Please use the ratings scales to indicate which characteristics you believe the ads reflect the most.*

*[AD]*

*Which personality characteristic does this ad reflect the most?”*

*
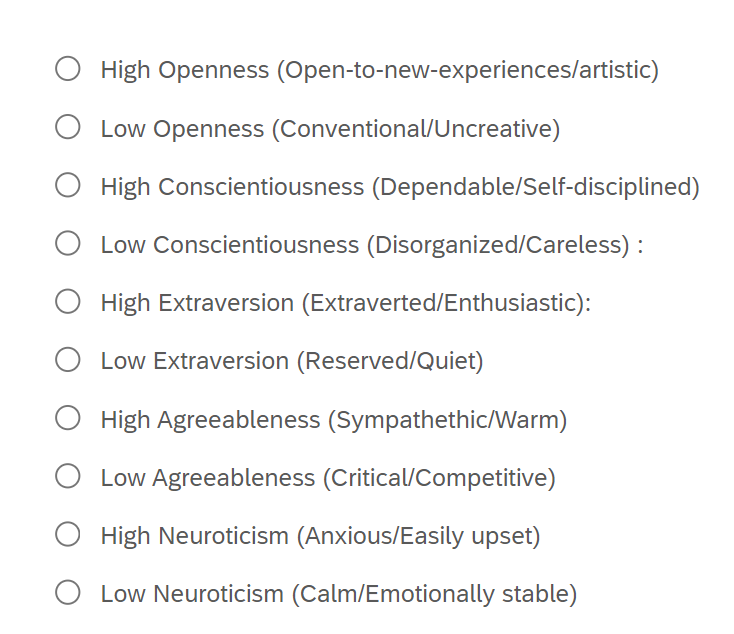
*

Given the ten different response options, the baseline likelihood of participants guessing the correct answer is 10%. Table S2 below shows the actual percentages for each of the eight messages.

**Table S2.** Percentage of participants correctly identifying the targeted trait

|  | Percentage correct | |
| --- | --- | --- |
| Trait | High | Low |
| OP | 20/20 (100%) | 15/20 (75%) |
| CO | 19/20 (95%) | 16/20 (80%) |
| EX | 20/20 (100%) | 16/20 (80%) |
| AG | 18/20 (90%) | 16/20 (80%) |

**Table S3.** Means, standard deviations, and zero-order correlations with confidence intervals of continuous variables in Study 1a

| Variable | *M* | *SD* | 1 | 2 | 3 | 4 | 5 | 6 | 7 | 8 | 9 | 10 |
| --- | --- | --- | --- | --- | --- | --- | --- | --- | --- | --- | --- | --- |
|  |  |  |  |  |  |  |  |  |  |  |  |  |
| 1. Openness (Ad) | 6.35 | 3.33 |  |  |  |  |  |  |  |  |  |  |
|  |  |  |  |  |  |  |  |  |  |  |  |  |
| 2. Conscient. (Ad) | 7.09 | 2.93 | .11 |  |  |  |  |  |  |  |  |  |
|  |  |  | [-.07, .29] |  |  |  |  |  |  |  |  |  |
|  |  |  |  |  |  |  |  |  |  |  |  |  |
| 3. Extraversion (Ad) | 5.44 | 3.01 | .23* | .09 |  |  |  |  |  |  |  |  |
|  |  |  | [.05, .39] | [-.09, .27] |  |  |  |  |  |  |  |  |
|  |  |  |  |  |  |  |  |  |  |  |  |  |
| 4. Agreeableness (Ad) | 7.22 | 3.16 | -.07 | -.27** | -.20* |  |  |  |  |  |  |  |
|  |  |  | [-.25, .11] | [-.43, -.10] | [-.37, -.02] |  |  |  |  |  |  |  |
|  |  |  |  |  |  |  |  |  |  |  |  |  |
| 5. Openness (P) | 5.01 | 1.04 | .29** | .07 | .02 | .05 |  |  |  |  |  |  |
|  |  |  | [.12, .45] | [-.11, .25] | [-.16, .20] | [-.13, .23] |  |  |  |  |  |  |
|  |  |  |  |  |  |  |  |  |  |  |  |  |
| 6. Conscient. (P) | 4.76 | 1.11 | -.05 | .16 | .05 | -.14 | .08 |  |  |  |  |  |
|  |  |  | [-.22, .13] | [-.02, .33] | [-.13, .23] | [-.31, .04] | [-.10, .26] |  |  |  |  |  |
|  |  |  |  |  |  |  |  |  |  |  |  |  |
| 7. Extraversion (P) | 3.78 | 1.15 | .04 | -.03 | .29** | -.02 | .36** | .39** |  |  |  |  |
|  |  |  | [-.14, .21] | [-.21, .15] | [.12, .44] | [-.20, .16] | [.19, .51] | [.22, .53] |  |  |  |  |
|  |  |  |  |  |  |  |  |  |  |  |  |  |
| 8. Agreeableness (P) | 5.08 | 0.98 | .04 | .11 | .12 | -.14 | .01 | .45** | .32** |  |  |  |
|  |  |  | [-.14, .22] | [-.07, .29] | [-.07, .29] | [-.31, .04] | [-.17, .19] | [.30, .58] | [.15, .47] |  |  |  |
|  |  |  |  |  |  |  |  |  |  |  |  |  |
| 9. Neuroticism (P) | 3.63 | 1.33 | .06 | .01 | -.13 | .09 | -.20* | -.52** | -.50** | -.31** |  |  |
|  |  |  | [-.12, .23] | [-.17, .19] | [-.30, .05] | [-.09, .26] | [-.37, -.02] | [-.64, -.37] | [-.62, -.35] | [-.46, -.14] |  |  |
|  |  |  |  |  |  |  |  |  |  |  |  |  |
| 10. Age | 37.16 | 13.23 | -.10 | -.15 | -.01 | -.06 | -.05 | .26** | .10 | .20* | -.32** |  |
|  |  |  | [-.28, .08] | [-.32, .03] | [-.19, .17] | [-.24, .12] | [-.23, .13] | [.09, .42] | [-.08, .27] | [.02, .36] | [-.47, -.15] |  |
|  |  |  |  |  |  |  |  |  |  |  |  |  |
| 11. Education | 4.22 | 1.32 | -.07 | -.08 | -.08 | -.02 | -.07 | .19* | .17 | .16 | -.13 | .11 |
|  |  |  | [-.24, .11] | [-.26, .10] | [-.26, .10] | [-.20, .16] | [-.24, .11] | [.01, .35] | [-.01, .34] | [-.02, .33] | [-.30, .05] | [-.07, .29] |
|  |  |  |  |  |  |  |  |  |  |  |  |  |

*Note.* *M* and *SD* are used to represent mean and standard deviation, respectively. Values in square brackets indicate the 95% confidence interval for each correlation. (Ad) indicates effectiveness ratings for the respective ads. (P) indicates participants’ personality scores on the respective trait. * indicates *p* < .05. ** indicates *p* < .01.

**Table S4.** Full model output of linear regression analyses in Study 1a

|  | **Openness** | | | **Conscientiousness** | | | **Extraversion** | | | **Agreeableness** | | |
| --- | --- | --- | --- | --- | --- | --- | --- | --- | --- | --- | --- | --- |
| *Predictors* | *β* | *CI_95_* | *p* | *β* | *CI_95_* | *p* | *β* | *CI_95_* | *p* | *β* | *CI_95_* | *p* |
| (Intercept) | .20 | -.13 – .53 | .851 | .25 | -.09 – .59 | .339 | .22 | -.11 – .55 | **.028** | -.23 | -.57 – .12 | **.014** |
| Openness | .36 | .16 – .56 | **<.001** | .10 | -.10 – .31 | .310 | -.10 | -.30 – .09 | .300 | .05 | -.16 – .26 | .640 |
| Conscientious. | -.05 | -.29 – .19 | .681 | .29 | .05 – .53 | **.020** | -.13 | -.36 – .11 | .289 | -.07 | -.32 – .18 | .564 |
| Extraversion | -.08 | -.32 – .16 | .527 | -.12 | -.37 – .12 | .319 | .40 | .16 – .63 | **.001** | -.03 | -.28 – .22 | .818 |
| Agreeableness | .17 | -.05 – .39 | .123 | .13 | -.10 – .35 | .261 | .07 | -.15 – .29 | .521 | -.17 | -.40 – .06 | .152 |
| Neuroticism | .14 | -.12 – .40 | .286 | .16 | -.11 – .43 | .240 | -.04 | -.30 – .21 | .738 | -.06 | -.33 – .22 | .672 |
| Age | .02 | -.21 – .26 | .837 | -.15 | -.40 – .09 | .212 | -.12 | -.35 – .12 | .320 | .05 | -.20 – .30 | .685 |
| Female | -.10 | -.53 – .33 | .646 | -.20 | -.64 – .24 | .371 | -.03 | -.45 – .40 | .897 | .45 | -.00 – .90 | .052 |
| Gender (Other) | .41 | -1.11 – 1.94 | .590 | .85 | -.71 – 2.41 | .284 | .11 | -1.39 – 1.61 | .888 | -.17 | -1.76 – 1.42 | .830 |
| Education | -.09 | -.28 – .10 | .343 | -.14 | -.34 – .06 | .166 | -.20 | -.39 – -.01 | **.040** | .05 | -.15 – .25 | .638 |
| Black | -.09 | -.75 – .57 | .784 | .08 | -.60 – .76 | .810 | -.80 | -1.45 – -.15 | **.017** | -.05 | -.74 – .64 | .883 |
| Asian | .55 | -.20 – 1.31 | .149 | .02 | -.75 – .80 | .956 | -.05 | -.79 – .69 | .897 | .11 | -.68 – .90 | .792 |
| Pacific Islander | .08 | -1.10 – 1.25 | .895 | -.68 | -1.88 – .53 | .267 | -.22 | -1.38 – .94 | .706 | .18 | -1.05 – 1.41 | .776 |
| Multi-racial | -.56 | -1.32 – .20 | .147 | -.28 | -1.06 – .50 | .473 | -.82 | -1.57 – -.07 | **.032** | .76 | -.03 – 1.56 | .060 |
| Part-time | -.08 | -.63 – .47 | .762 | .00 | -.56 – .57 | .991 | -.33 | -.87 – .21 | .226 | -.08 | -.65 – .50 | .784 |
| Self-employed | -.19 | -.82 – .44 | .554 | -.43 | -1.08 – .22 | .195 | -.09 | -.72 – .53 | .772 | -.03 | -.69 – .64 | .938 |
| Unemployed | -.68 | -1.41 – .06 | .071 | -.40 | -1.16 – .35 | .294 | -.14 | -.86 – .59 | .705 | .29 | -.49 – 1.06 | .464 |
| Retired | -.30 | -1.01 – .41 | .406 | -.39 | -1.12 – .34 | .289 | -.38 | -1.08 – .32 | .282 | -.09 | -.83 – .66 | .813 |
| Student | -.97 | -2.13 – .18 | .097 | -.30 | -1.49 – .88 | .614 | .21 | -.93 – 1.35 | .714 | -.50 | -1.71 – .70 | .409 |
| Employ. (Other) | .06 | -.92 – 1.03 | .904 | -.48 | -1.48 – .52 | .346 | .58 | -.38 – 1.54 | .231 | -.91 | -1.93 – .11 | .079 |
| Observations | 120 | | | 120 | | | 120 | | | 120 | | |
| R^2^ / R^2^ adjusted | .213 / .064 | | | .173 / .015 | | | .237 / .092 | | | .139 / -.025 | | |

**Study 1b**

**Sample demographics**

| Age | 36.2 (12.5 SD) |
| --- | --- |
| Gender | 48% Female |
| Ethnicity | 77% White  5% Black or African American  1% American Indian, Native American or Alaska Native  10% Asian  0% Hawaiian Native or Other Pacific Islander  1% Other  6% Multiracial |
| Education | 1% Some high school or less  13% High school diploma or GED  21% Some college but no degree  13% Associates or technical degree  48% Bachelor’s degree  14% Graduate or professional degree (MA, MS, MBA, PHD, JD, MD etc.) |
| Income | 20% Less than $10,000  8% $10,000 to $14,999  8% $15,000 to $24,999  10% $25,000 to $34,999  13% $35,000 to $49,999  19% $50,000 to $74,999  11% $75,000 to $99,999  7% $100,000 to $149,999  3% $150,000 to $199,999  1% $200,000 or more |
| Employment | 49% Employed (full-time)  11% Employed (part-time)  12% Self-employed  14% Unemployed  8% Student  3% Retired  3% Other |

**Table S5.** *Means, standard deviations, and zero-order correlations with confidence intervals of continuous variables in Study 1b*

| Variable | *M* | *SD* | 1 | 2 | 3 | 4 | 5 | 6 | 7 | 8 | 9 | 10 |
| --- | --- | --- | --- | --- | --- | --- | --- | --- | --- | --- | --- | --- |
|  |  |  |  |  |  |  |  |  |  |  |  |  |
| 1. Openness (Ad) | 7.05 | 3.12 |  |  |  |  |  |  |  |  |  |  |
|  |  |  |  |  |  |  |  |  |  |  |  |  |
| 2. Conscient. (Ad) | 6.21 | 3.21 | .11* |  |  |  |  |  |  |  |  |  |
|  |  |  | [.02, .20] |  |  |  |  |  |  |  |  |  |
|  |  |  |  |  |  |  |  |  |  |  |  |  |
| 3. Extraversion (Ad) | 5.58 | 3.00 | .19** | .04 |  |  |  |  |  |  |  |  |
|  |  |  | [.10, .28] | [-.05, .13] |  |  |  |  |  |  |  |  |
|  |  |  |  |  |  |  |  |  |  |  |  |  |
| 4. Agreeableness (Ad) | 7.01 | 3.05 | -.05 | -.22** | -.11* |  |  |  |  |  |  |  |
|  |  |  | [-.14, .04] | [-.31, -.14] | [-.20, -.02] |  |  |  |  |  |  |  |
|  |  |  |  |  |  |  |  |  |  |  |  |  |
| 5. Openness (P) | 5.15 | 1.13 | .17** | -.01 | -.06 | .01 |  |  |  |  |  |  |
|  |  |  | [.08, .26] | [-.10, .08] | [-.15, .03] | [-.08, .10] |  |  |  |  |  |  |
|  |  |  |  |  |  |  |  |  |  |  |  |  |
| 6. Conscient. (P) | 4.77 | 1.17 | .08 | .14** | .01 | -.03 | .19** |  |  |  |  |  |
|  |  |  | [-.01, .17] | [.05, .23] | [-.08, .10] | [-.12, .07] | [.10, .28] |  |  |  |  |  |
|  |  |  |  |  |  |  |  |  |  |  |  |  |
| 7. Extraversion (P) | 3.65 | 1.21 | .07 | .00 | .18** | -.02 | .22** | .37** |  |  |  |  |
|  |  |  | [-.02, .16] | [-.09, .10] | [.10, .27] | [-.11, .07] | [.13, .31] | [.28, .44] |  |  |  |  |
|  |  |  |  |  |  |  |  |  |  |  |  |  |
| 8. Agreeableness (P) | 5.07 | 1.00 | .07 | .05 | .03 | .00 | .28** | .40** | .24** |  |  |  |
|  |  |  | [-.02, .16] | [-.04, .14] | [-.06, .12] | [-.09, .09] | [.19, .36] | [.32, .48] | [.16, .33] |  |  |  |
|  |  |  |  |  |  |  |  |  |  |  |  |  |
| 9. Neuroticism (P) | 3.77 | 1.40 | -.05 | -.02 | .02 | .06 | -.19** | -.49** | -.46** | -.38** |  |  |
|  |  |  | [-.14, .04] | [-.11, .07] | [-.07, .11] | [-.03, .15] | [-.28, -.10] | [-.56, -.42] | [-.53, -.38] | [-.45, -.29] |  |  |
|  |  |  |  |  |  |  |  |  |  |  |  |  |
| 10. Age | 36.15 | 12.49 | -.06 | -.02 | -.05 | -.05 | .07 | .21** | .12* | .22** | -.31** |  |
|  |  |  | [-.15, .04] | [-.11, .07] | [-.14, .04] | [-.14, .04] | [-.02, .16] | [.12, .29] | [.03, .20] | [.14, .31] | [-.39, -.23] |  |
|  |  |  |  |  |  |  |  |  |  |  |  |  |
| 11. Education | 4.17 | 1.32 | -.06 | -.04 | .05 | .04 | -.01 | .17** | .12** | .05 | -.10* | .15** |
|  |  |  | [-.15, .03] | [-.13, .05] | [-.04, .14] | [-.05, .13] | [-.10, .08] | [.08, .25] | [.03, .21] | [-.04, .14] | [-.19, -.01] | [.06, .24] |
|  |  |  |  |  |  |  |  |  |  |  |  |  |

*Note.* *M* and *SD* are used to represent mean and standard deviation, respectively. Values in square brackets indicate the 95% confidence interval for each correlation. (Ad) indicates effectiveness ratings for the respective ads. (P) indicates participants’ personality scores on the respective trait. * indicates *p* < .05. ** indicates *p* < .01.

**Table S6.** Full model output of linear regression analyses in Study 1b

|  | **Openness** | | | **Conscientiousness** | | | **Extraversion** | | | **Agreeableness** | | |
| --- | --- | --- | --- | --- | --- | --- | --- | --- | --- | --- | --- | --- |
| *Predictors* | *β* | *CI_95_* | *p* | *β* | *CI_95_* | *p* | *β* | *CI_95_* | *p* | *β* | *CI_95_* | *p* |
| (Intercept) | -0.05 | -0.21 – 0.11 | **0.004** | 0.03 | -0.14 – 0.19 | **0.015** | -0.08 | -0.24 – 0.09 | 0.074 | -0.21 | -0.38 – -0.05 | **<0.001** |
| Openness | 0.16 | 0.06 – 0.25 | **0.001** | -0.05 | -0.14 – 0.05 | 0.355 | -0.12 | -0.21 – -0.02 | **0.017** | 0.00 | -0.09 – 0.10 | 0.923 |
| Conscientious. | 0.06 | -0.05 – 0.17 | 0.289 | 0.20 | 0.09 – 0.31 | **<0.001** | -0.01 | -0.12 – 0.10 | 0.817 | -0.01 | -0.12 – 0.11 | 0.905 |
| Extraversion | 0.04 | -0.07 – 0.14 | 0.481 | 0.01 | -0.10 – 0.12 | 0.876 | 0.29 | 0.18 – 0.39 | **<0.001** | 0.02 | -0.09 – 0.13 | 0.756 |
| Agreeableness | 0.03 | -0.08 – 0.13 | 0.620 | 0.03 | -0.08 – 0.14 | 0.580 | 0.05 | -0.05 – 0.16 | 0.334 | 0.00 | -0.10 – 0.11 | 0.952 |
| Neuroticism | -0.02 | -0.14 – 0.10 | 0.779 | 0.08 | -0.04 – 0.21 | 0.181 | 0.11 | -0.01 – 0.24 | 0.067 | 0.01 | -0.11 – 0.14 | 0.847 |
| Age | -0.09 | -0.21 – 0.02 | 0.120 | -0.05 | -0.17 – 0.06 | 0.385 | -0.06 | -0.17 – 0.06 | 0.311 | -0.04 | -0.15 – 0.08 | 0.534 |
| Female | 0.04 | -0.15 – 0.24 | 0.663 | -0.12 | -0.32 – 0.08 | 0.241 | 0.04 | -0.16 – 0.23 | 0.723 | 0.18 | -0.02 – 0.38 | 0.084 |
| Gender (Other) | -0.10 | -0.66 – 0.45 | 0.721 | -0.23 | -0.79 – 0.34 | 0.427 | -0.28 | -0.84 – 0.27 | 0.318 | 0.14 | -0.43 – 0.71 | 0.635 |
| Education | -0.05 | -0.15 – 0.04 | 0.276 | -0.05 | -0.15 – 0.05 | 0.368 | 0.06 | -0.04 – 0.16 | 0.220 | 0.07 | -0.03 – 0.17 | 0.163 |
| Black | -0.66 | -1.08 – -0.25 | **0.002** | 0.10 | -0.32 – 0.52 | 0.649 | 0.01 | -0.41 – 0.43 | 0.951 | -0.26 | -0.69 – 0.16 | 0.221 |
| Native American | 0.55 | -0.44 – 1.54 | 0.276 | 0.26 | -0.74 – 1.27 | 0.609 | 0.46 | -0.54 – 1.45 | 0.366 | -0.55 | -1.56 – 0.47 | 0.291 |
| Asian | 0.21 | -0.11 – 0.53 | 0.196 | -0.13 | -0.45 – 0.20 | 0.446 | -0.07 | -0.39 – 0.25 | 0.663 | 0.15 | -0.18 – 0.47 | 0.375 |
| Pacific Islander | 0.67 | -0.09 – 1.42 | 0.082 | -0.04 | -0.80 – 0.72 | 0.918 | 0.18 | -0.57 – 0.94 | 0.634 | 0.23 | -0.54 – 1.00 | 0.561 |
| Multi-racial | 0.30 | -0.08 – 0.68 | 0.121 | 0.37 | -0.01 – 0.76 | 0.057 | -0.09 | -0.47 – 0.29 | 0.649 | 0.06 | -0.33 – 0.45 | 0.746 |
| Part-time | -0.14 | -0.45 – 0.17 | 0.377 | 0.34 | 0.03 – 0.65 | **0.034** | 0.18 | -0.13 – 0.49 | 0.261 | 0.39 | 0.08 – 0.71 | **0.015** |
| Self-employed | -0.06 | -0.36 – 0.24 | 0.713 | -0.10 | -0.41 – 0.20 | 0.516 | 0.15 | -0.15 – 0.46 | 0.317 | 0.23 | -0.07 – 0.54 | 0.136 |
| Unemployed | 0.13 | -0.16 – 0.43 | 0.373 | 0.13 | -0.17 – 0.42 | 0.411 | 0.25 | -0.05 – 0.54 | 0.106 | 0.16 | -0.14 – 0.47 | 0.285 |
| Retired | 0.00 | -0.36 – 0.37 | 0.992 | -0.26 | -0.63 – 0.11 | 0.170 | 0.01 | -0.36 – 0.38 | 0.947 | 0.17 | -0.20 – 0.55 | 0.365 |
| Student | 0.34 | -0.20 – 0.89 | 0.218 | 0.13 | -0.43 – 0.68 | 0.654 | -0.15 | -0.70 – 0.40 | 0.581 | -0.09 | -0.64 – 0.47 | 0.764 |
| Employ. (Other) | 0.16 | -0.40 – 0.72 | 0.572 | -0.22 | -0.78 – 0.34 | 0.444 | 0.28 | -0.28 – 0.83 | 0.333 | 0.59 | 0.02 – 1.16 | **0.041** |
| Observations | 471 | | | 471 | | | 471 | | | 471 | | |
| R^2^ / R^2^ adjusted | 0.090 / 0.049 | | | 0.063 / 0.022 | | | 0.078 / 0.037 | | | 0.047 / 0.005 | | |

**Table S7.** Full model output of linear regression analyses with interaction terms between traits and conditions in Study 1b

|  | **Openness** | | | **Conscientiousness** | | | **Extraversion** | | | **Agreeableness** | | |
| --- | --- | --- | --- | --- | --- | --- | --- | --- | --- | --- | --- | --- |
| *Predictors* | *β* | *CI_95_* | *p* | *β* | *CI_95_* | *p* | *β* | *CI_95_* | *p* | *β* | *CI_95_* | *p* |
| (Intercept) | -0.06 | -0.27 – 0.16 | 0.603 | -0.07 | -0.28 – 0.15 | 0.541 | -0.19 | -0.40 – 0.03 | 0.092 | -0.20 | -0.42 – 0.02 | 0.068 |
| Openness (OP) | 0.15 | -0.01 – 0.30 | 0.069 | -0.05 | -0.15 – 0.05 | 0.323 | -0.12 | -0.22 – -0.02 | **0.017** | 0.01 | -0.09 – 0.11 | 0.874 |
| OP × Disclosure 1 | -0.02 | -0.25 – 0.20 | 0.857 | - | - | - | - | - | - | - | - | - |
| OP × Disclosure 2 | 0.05 | -0.17 – 0.26 | 0.671 | - | - | - | - | - | - | - | - | - |
| Conscientious. (CO) | 0.06 | -0.05 – 0.17 | 0.284 | 0.31 | 0.13 – 0.49 | **0.001** | -0.01 | -0.12 – 0.10 | 0.808 | -0.01 | -0.12 – 0.10 | 0.889 |
| CO × Disclosure 1 | - | - | - | -0.15 | -0.38 – 0.09 | 0.215 | - | - | - | - | - | - |
| CO × Disclosure 2 | - | - | - | -0.17 | -0.39 – 0.06 | 0.147 | - | - | - | - | - | - |
| Extraversion (EX) | 0.04 | -0.07 – 0.15 | 0.471 | 0.01 | -0.10 – 0.12 | 0.875 | 0.33 | 0.17 – 0.50 | **<0.001** | 0.01 | -0.10 – 0.12 | 0.797 |
| EX × Disclosure 1 | - | - | - | - | - | - | -0.08 | -0.31 – 0.14 | 0.468 | - | - | - |
| EX × Disclosure 2 | - | - | - | - | - | - | -0.05 | -0.28 – 0.17 | 0.635 | - | - | - |
| Agreeableness (AG) | 0.03 | -0.08 – 0.14 | 0.588 | 0.02 | -0.09 – 0.13 | 0.679 | 0.05 | -0.06 – 0.15 | 0.410 | -0.03 | -0.21 – 0.14 | 0.692 |
| AG × condition [2] | - | - | - | - | - | - | - | - | - | 0.05 | -0.19 – 0.29 | 0.657 |
| AG × condition [3] | - | - | - | - | - | - | - | - | - | 0.05 | -0.17 – 0.27 | 0.640 |
| Neuroticism (NE) | -0.01 | -0.14 – 0.11 | 0.817 | 0.07 | -0.05 – 0.20 | 0.237 | 0.11 | -0.02 – 0.23 | 0.086 | 0.01 | -0.11 – 0.14 | 0.859 |
| Disclosure 1 | -0.01 | -0.24 – 0.22 | 0.915 | 0.09 | -0.14 – 0.32 | 0.444 | 0.16 | -0.07 – 0.40 | 0.163 | 0.02 | -0.21 – 0.26 | 0.841 |
| Disclosure 2 | 0.02 | -0.19 – 0.24 | 0.830 | 0.16 | -0.06 – 0.38 | 0.144 | 0.13 | -0.09 – 0.35 | 0.233 | -0.05 | -0.27 – 0.18 | 0.683 |
| Age | -0.09 | -0.21 – 0.02 | 0.121 | -0.05 | -0.17 – 0.06 | 0.375 | -0.06 | -0.17 – 0.06 | 0.329 | -0.04 | -0.15 – 0.08 | 0.544 |
| Female | 0.05 | -0.15 – 0.24 | 0.653 | -0.11 | -0.31 – 0.09 | 0.292 | 0.05 | -0.15 – 0.24 | 0.648 | 0.18 | -0.02 – 0.38 | 0.085 |
| Gender (Other) | -0.10 | -0.66 – 0.46 | 0.715 | -0.17 | -0.74 – 0.39 | 0.546 | -0.24 | -0.80 – 0.32 | 0.398 | 0.13 | -0.44 – 0.70 | 0.649 |
| Education | -0.05 | -0.15 – 0.05 | 0.290 | -0.06 | -0.16 – 0.04 | 0.276 | 0.06 | -0.04 – 0.16 | 0.256 | 0.08 | -0.03 – 0.18 | 0.146 |
| Black | -0.67 | -1.09 – -0.25 | **0.002** | 0.11 | -0.31 – 0.53 | 0.612 | 0.04 | -0.38 – 0.47 | 0.837 | -0.27 | -0.70 – 0.16 | 0.224 |
| Native American | 0.55 | -0.45 – 1.55 | 0.279 | 0.32 | -0.68 – 1.33 | 0.530 | 0.54 | -0.46 – 1.55 | 0.290 | -0.56 | -1.58 – 0.46 | 0.278 |
| Asian | 0.21 | -0.12 – 0.53 | 0.208 | -0.12 | -0.45 – 0.20 | 0.453 | -0.08 | -0.40 – 0.25 | 0.642 | 0.14 | -0.19 – 0.47 | 0.392 |
| Pacific Islander | 0.67 | -0.09 – 1.42 | 0.083 | -0.08 | -0.85 – 0.68 | 0.829 | 0.14 | -0.62 – 0.91 | 0.709 | 0.21 | -0.56 – 0.99 | 0.588 |
| Multi-racial | 0.30 | -0.09 – 0.68 | 0.129 | 0.39 | 0.00 – 0.78 | **0.048** | -0.07 | -0.45 – 0.32 | 0.737 | 0.06 | -0.33 – 0.46 | 0.753 |
| Part-time | -0.14 | -0.45 – 0.17 | 0.366 | 0.34 | 0.03 – 0.66 | **0.032** | 0.18 | -0.13 – 0.50 | 0.246 | 0.39 | 0.07 – 0.71 | **0.016** |
| Self-employed | -0.06 | -0.36 – 0.24 | 0.706 | -0.11 | -0.41 – 0.20 | 0.490 | 0.15 | -0.15 – 0.45 | 0.326 | 0.24 | -0.07 – 0.55 | 0.126 |
| Unemployed | 0.14 | -0.16 – 0.43 | 0.370 | 0.10 | -0.20 – 0.40 | 0.511 | 0.24 | -0.06 – 0.54 | 0.119 | 0.17 | -0.13 – 0.48 | 0.269 |
| Retired | 0.01 | -0.36 – 0.38 | 0.967 | -0.26 | -0.63 – 0.11 | 0.167 | 0.01 | -0.36 – 0.38 | 0.965 | 0.16 | -0.21 – 0.54 | 0.390 |
| Student | 0.34 | -0.21 – 0.89 | 0.220 | 0.13 | -0.42 – 0.69 | 0.639 | -0.16 | -0.71 – 0.39 | 0.568 | -0.10 | -0.66 – 0.47 | 0.737 |
| Employ. (Other) | 0.16 | -0.40 – 0.72 | 0.578 | -0.20 | -0.76 – 0.36 | 0.484 | 0.28 | -0.28 – 0.84 | 0.333 | 0.59 | 0.02 – 1.17 | **0.042** |
| Observations | 471 | | | 471 | | | 471 | | | 471 | | |
| R^2^ / R^2^ adjusted | 0.091 / 0.042 | | | 0.073 / 0.023 | | | 0.084 / 0.034 | | | 0.049 / -0.002 | | |

**Table S8.** Full model output of linear regression analyses for the ‘Baseline’ Condition in Study 1b

|  | **Openness** | | | **Conscientiousness** | | | **Extraversion** | | | **Agreeableness** | | |
| --- | --- | --- | --- | --- | --- | --- | --- | --- | --- | --- | --- | --- |
| *Predictors* | *β* | *CI_95_* | *p* | *β* | *CI_95_* | *p* | *β* | *CI_95_* | *p* | *β* | *CI_95_* | *p* |
| (Intercept) | -0.03 | -0.32 – 0.26 | 0.430 | 0.12 | -0.17 – 0.41 | 0.185 | 0.00 | -0.30 – 0.30 | 0.093 | -0.14 | -0.44 – 0.16 | 0.263 |
| Openness | 0.10 | -0.08 – 0.28 | 0.269 | -0.11 | -0.29 – 0.08 | 0.247 | 0.00 | -0.19 – 0.19 | 0.979 | 0.06 | -0.13 – 0.24 | 0.552 |
| Conscientious. | 0.05 | -0.15 – 0.24 | 0.642 | 0.30 | 0.10 – 0.49 | **0.003** | -0.09 | -0.29 – 0.11 | 0.369 | 0.06 | -0.14 – 0.26 | 0.550 |
| Extraversion | 0.10 | -0.09 – 0.29 | 0.287 | -0.04 | -0.24 – 0.15 | 0.658 | 0.31 | 0.11 – 0.51 | **0.002** | 0.06 | -0.14 – 0.26 | 0.564 |
| Agreeableness | 0.20 | -0.00 – 0.40 | 0.053 | 0.02 | -0.19 – 0.22 | 0.869 | -0.01 | -0.22 – 0.21 | 0.954 | -0.03 | -0.24 – 0.18 | 0.763 |
| Neuroticism | 0.11 | -0.09 – 0.32 | 0.284 | 0.03 | -0.18 – 0.24 | 0.809 | 0.02 | -0.20 – 0.24 | 0.840 | 0.12 | -0.09 – 0.34 | 0.260 |
| Age | -0.16 | -0.36 – 0.03 | 0.101 | 0.03 | -0.17 – 0.23 | 0.758 | -0.14 | -0.35 – 0.06 | 0.164 | -0.01 | -0.21 – 0.19 | 0.918 |
| Female | -0.08 | -0.43 – 0.27 | 0.654 | -0.32 | -0.68 – 0.04 | 0.078 | 0.01 | -0.36 – 0.38 | 0.959 | 0.12 | -0.25 – 0.48 | 0.524 |
| Gender (Other) | -0.23 | -1.05 – 0.59 | 0.579 | -0.20 | -1.02 – 0.63 | 0.638 | -0.20 | -1.05 – 0.66 | 0.646 | 0.29 | -0.56 – 1.14 | 0.503 |
| Education | -0.09 | -0.26 – 0.09 | 0.333 | -0.03 | -0.21 – 0.15 | 0.744 | 0.02 | -0.16 – 0.21 | 0.824 | 0.12 | -0.07 – 0.30 | 0.214 |
| Black | -0.82 | -1.45 – -0.18 | **0.012** | 0.19 | -0.45 – 0.83 | 0.560 | 0.33 | -0.34 – 0.99 | 0.331 | -0.44 | -1.09 – 0.22 | 0.193 |
| Native American | 1.21 | 0.01 – 2.40 | **0.047** | 0.38 | -0.83 – 1.58 | 0.538 | 0.61 | -0.64 – 1.85 | 0.340 | -0.24 | -1.48 – 1.00 | 0.700 |
| Asian | -0.17 | -0.80 – 0.45 | 0.580 | 0.32 | -0.31 – 0.95 | 0.311 | -0.30 | -0.95 – 0.35 | 0.370 | 0.26 | -0.39 – 0.90 | 0.431 |
| Pacific Islander | 1.48 | -0.55 – 3.52 | 0.152 | -1.54 | -3.60 – 0.51 | 0.140 | -1.66 | -3.79 – 0.47 | 0.126 | 1.59 | -0.52 – 3.70 | 0.139 |
| Multi-racial | 0.22 | -0.33 – 0.78 | 0.421 | 0.57 | 0.01 – 1.13 | **0.045** | 0.03 | -0.55 – 0.60 | 0.922 | 0.14 | -0.43 – 0.71 | 0.624 |
| Part-time | 0.26 | -0.26 – 0.78 | 0.324 | 0.27 | -0.25 – 0.80 | 0.303 | 0.21 | -0.33 – 0.75 | 0.435 | 0.42 | -0.12 – 0.95 | 0.126 |
| Self-employed | -0.08 | -0.61 – 0.46 | 0.780 | -0.10 | -0.64 – 0.44 | 0.725 | -0.07 | -0.63 – 0.49 | 0.815 | -0.02 | -0.58 – 0.53 | 0.933 |
| Unemployed | 0.42 | -0.10 – 0.93 | 0.111 | -0.10 | -0.62 – 0.42 | 0.716 | -0.12 | -0.65 – 0.42 | 0.672 | 0.28 | -0.26 – 0.81 | 0.307 |
| Retired | 0.22 | -0.48 – 0.92 | 0.530 | -0.36 | -1.06 – 0.35 | 0.314 | -0.18 | -0.91 – 0.55 | 0.631 | -0.37 | -1.10 – 0.35 | 0.308 |
| Student | 0.50 | -0.45 – 1.45 | 0.302 | -0.22 | -1.19 – 0.74 | 0.644 | 0.13 | -0.87 – 1.12 | 0.803 | -0.66 | -1.64 – 0.33 | 0.191 |
| Employ. (Other) | -0.25 | -1.18 – 0.67 | 0.590 | -0.46 | -1.40 – 0.48 | 0.332 | 0.09 | -0.88 – 1.06 | 0.857 | 0.78 | -0.18 – 1.74 | 0.111 |
| Observations | 154 | | | 154 | | | 154 | | | 154 | | |
| R^2^ / R^2^ adjusted | 0.200 / 0.080 | | | 0.182 / 0.059 | | | 0.125 / -0.007 | | | 0.139 / 0.009 | | |

**Table S9.** Full model output of linear regression analyses for the ‘Disclosure 1’ Condition in Study 1b

|  | **Openness** | | | **Conscientiousness** | | | **Extraversion** | | | **Agreeableness** | | |
| --- | --- | --- | --- | --- | --- | --- | --- | --- | --- | --- | --- | --- |
| *Predictors* | *β* | *CI_95_* | *p* | *β* | *CI_95_* | *p* | *β* | *CI_95_* | *p* | *β* | *CI_95_* | *p* |
| (Intercept) | -0.26 | -0.57 – 0.05 | 0.072 | 0.04 | -0.30 – 0.37 | 0.228 | -0.33 | -0.65 – -0.00 | 0.495 | -0.25 | -0.59 – 0.09 | 0.207 |
| Openness | 0.07 | -0.11 – 0.24 | 0.452 | -0.06 | -0.25 – 0.13 | 0.512 | -0.20 | -0.38 – -0.01 | **0.034** | 0.01 | -0.18 – 0.20 | 0.914 |
| Conscientious. | -0.05 | -0.25 – 0.14 | 0.584 | 0.13 | -0.08 – 0.34 | 0.233 | 0.15 | -0.05 – 0.35 | 0.142 | -0.01 | -0.22 – 0.20 | 0.938 |
| Extraversion | -0.06 | -0.25 – 0.13 | 0.518 | 0.08 | -0.12 – 0.28 | 0.440 | 0.21 | 0.02 – 0.41 | **0.033** | 0.01 | -0.20 – 0.22 | 0.918 |
| Agreeableness | 0.14 | -0.06 – 0.34 | 0.157 | 0.12 | -0.09 – 0.33 | 0.252 | 0.06 | -0.14 – 0.26 | 0.556 | 0.08 | -0.14 – 0.29 | 0.470 |
| Neuroticism | -0.16 | -0.37 – 0.05 | 0.128 | 0.09 | -0.14 – 0.31 | 0.450 | 0.10 | -0.12 – 0.32 | 0.363 | 0.11 | -0.12 – 0.33 | 0.354 |
| Age | 0.06 | -0.16 – 0.27 | 0.595 | -0.21 | -0.44 – 0.02 | 0.079 | 0.01 | -0.22 – 0.23 | 0.959 | -0.09 | -0.32 – 0.15 | 0.467 |
| Female | 0.24 | -0.12 – 0.59 | 0.195 | -0.11 | -0.49 – 0.28 | 0.583 | 0.32 | -0.05 – 0.69 | 0.094 | 0.13 | -0.26 – 0.52 | 0.521 |
| Gender (Other) | -0.46 | -1.61 – 0.70 | 0.433 | 1.04 | -0.19 – 2.28 | 0.097 | -0.49 | -1.68 – 0.71 | 0.423 | -0.34 | -1.60 – 0.92 | 0.590 |
| Education | -0.03 | -0.21 – 0.16 | 0.760 | -0.01 | -0.21 – 0.19 | 0.925 | -0.01 | -0.20 – 0.18 | 0.931 | 0.08 | -0.12 – 0.28 | 0.428 |
| Black | -0.89 | -1.70 – -0.08 | **0.032** | -0.43 | -1.30 – 0.43 | 0.323 | -0.09 | -0.93 – 0.75 | 0.828 | 0.58 | -0.30 – 1.46 | 0.197 |
| Asian | 0.43 | -0.10 – 0.96 | 0.112 | -0.12 | -0.69 – 0.45 | 0.676 | -0.06 | -0.61 – 0.49 | 0.829 | 0.23 | -0.35 – 0.81 | 0.441 |
| Pacific Islander | 0.84 | -0.31 – 1.98 | 0.150 | -0.23 | -1.45 – 1.00 | 0.715 | 0.41 | -0.78 – 1.60 | 0.496 | 0.47 | -0.78 – 1.72 | 0.459 |
| Multi-racial | 0.75 | -0.07 – 1.58 | 0.074 | 0.94 | 0.06 – 1.83 | **0.036** | -0.37 | -1.23 – 0.49 | 0.395 | -0.73 | -1.63 – 0.18 | 0.115 |
| Part-time | -0.39 | -0.93 – 0.16 | 0.161 | 0.11 | -0.47 – 0.69 | 0.711 | 0.24 | -0.32 – 0.80 | 0.398 | 0.24 | -0.35 – 0.83 | 0.421 |
| Self-employed | 0.46 | -0.13 – 1.04 | 0.125 | -0.32 | -0.95 – 0.30 | 0.312 | 0.68 | 0.07 – 1.29 | **0.028** | 0.48 | -0.16 – 1.11 | 0.143 |
| Unemployed | 0.06 | -0.49 – 0.60 | 0.833 | -0.02 | -0.60 – 0.56 | 0.956 | 0.44 | -0.12 – 1.01 | 0.121 | 0.43 | -0.17 – 1.02 | 0.156 |
| Retired | 0.18 | -0.42 – 0.78 | 0.551 | -0.23 | -0.87 – 0.41 | 0.477 | 0.33 | -0.29 – 0.94 | 0.300 | 0.08 | -0.57 – 0.73 | 0.813 |
| Student | 0.55 | -0.36 – 1.45 | 0.233 | 0.62 | -0.35 – 1.58 | 0.210 | -0.20 | -1.13 – 0.74 | 0.681 | 0.22 | -0.76 – 1.21 | 0.654 |
| Employ. (Other) | 1.07 | 0.05 – 2.10 | **0.040** | 0.56 | -0.53 – 1.66 | 0.309 | 0.61 | -0.45 – 1.67 | 0.258 | 0.56 | -0.56 – 1.67 | 0.327 |
| Observations | 141 | | | 141 | | | 141 | | | 141 | | |
| R^2^ / R^2^ adjusted | 0.244 / 0.126 | | | 0.136 / 0.000 | | | 0.187 / 0.059 | | | 0.098 / -0.043 | | |

**Table S10.** Full model output of linear regression analyses for the ‘Disclosure 2’ Condition in Study 1b

|  | **Openness** | | | **Conscientiousness** | | | **Extraversion** | | | **Agreeableness** | | |
| --- | --- | --- | --- | --- | --- | --- | --- | --- | --- | --- | --- | --- |
| *Predictors* | *β* | *CI_95_* | *p* | *β* | *CI_95_* | *p* | *β* | *CI_95_* | *p* | *β* | *CI_95_* | *p* |
| (Intercept) | 0.08 | -0.19 – 0.35 | 0.084 | -0.05 | -0.32 – 0.22 | 0.094 | 0.03 | -0.24 – 0.30 | 0.479 | -0.30 | -0.58 – -0.03 | **<0.001** |
| Openness | 0.22 | 0.06 – 0.39 | **0.007** | 0.02 | -0.14 – 0.18 | 0.798 | -0.14 | -0.30 – 0.02 | 0.093 | -0.04 | -0.20 – 0.12 | 0.626 |
| Conscientious. | 0.10 | -0.10 – 0.29 | 0.325 | 0.20 | 0.01 – 0.39 | **0.037** | -0.07 | -0.26 – 0.12 | 0.453 | -0.07 | -0.26 – 0.12 | 0.458 |
| Extraversion | 0.11 | -0.08 – 0.30 | 0.235 | 0.07 | -0.11 – 0.26 | 0.440 | 0.31 | 0.12 – 0.50 | **0.001** | -0.08 | -0.27 – 0.11 | 0.412 |
| Agreeableness | -0.17 | -0.35 – 0.01 | 0.064 | -0.10 | -0.28 – 0.08 | 0.285 | 0.05 | -0.13 – 0.23 | 0.577 | -0.02 | -0.20 – 0.16 | 0.799 |
| Neuroticism | 0.07 | -0.17 – 0.30 | 0.578 | 0.10 | -0.12 – 0.33 | 0.368 | 0.19 | -0.04 – 0.42 | 0.112 | -0.20 | -0.43 – 0.03 | 0.086 |
| Age | -0.06 | -0.25 – 0.14 | 0.582 | -0.06 | -0.26 – 0.14 | 0.557 | 0.01 | -0.18 – 0.21 | 0.891 | -0.04 | -0.23 – 0.16 | 0.716 |
| Female | -0.07 | -0.41 – 0.28 | 0.704 | -0.05 | -0.39 – 0.29 | 0.771 | -0.17 | -0.51 – 0.18 | 0.337 | 0.38 | 0.04 – 0.72 | **0.030** |
| Gender (Other) | 0.40 | -0.84 – 1.65 | 0.522 | -1.09 | -2.33 – 0.14 | 0.081 | -0.25 | -1.49 – 0.99 | 0.691 | 0.61 | -0.62 – 1.85 | 0.327 |
| Education | -0.05 | -0.22 – 0.13 | 0.594 | -0.08 | -0.26 – 0.09 | 0.347 | 0.16 | -0.01 – 0.34 | 0.065 | 0.00 | -0.17 – 0.17 | 0.986 |
| Black | -0.06 | -0.87 – 0.75 | 0.888 | 0.57 | -0.23 – 1.38 | 0.162 | 0.05 | -0.76 – 0.86 | 0.897 | -0.70 | -1.51 – 0.11 | 0.088 |
| Native American | -0.81 | -3.22 – 1.61 | 0.510 | 0.82 | -1.57 – 3.22 | 0.498 | 0.44 | -1.96 – 2.85 | 0.718 | -0.56 | -2.96 – 1.84 | 0.644 |
| Asian | 0.36 | -0.20 – 0.91 | 0.208 | -0.38 | -0.93 – 0.17 | 0.175 | 0.05 | -0.50 – 0.61 | 0.849 | 0.14 | -0.41 – 0.69 | 0.607 |
| Pacific Islander | 0.55 | -0.68 – 1.77 | 0.377 | 0.35 | -0.86 – 1.56 | 0.571 | 0.51 | -0.71 – 1.72 | 0.414 | -0.56 | -1.78 – 0.65 | 0.360 |
| Multi-racial | 0.32 | -0.42 – 1.06 | 0.395 | -0.24 | -0.97 – 0.49 | 0.520 | 0.30 | -0.44 – 1.04 | 0.422 | 0.44 | -0.29 – 1.18 | 0.234 |
| Part-time | -0.31 | -0.89 – 0.27 | 0.297 | 0.59 | 0.01 – 1.16 | **0.046** | 0.13 | -0.44 – 0.71 | 0.650 | 0.60 | 0.03 – 1.18 | **0.040** |
| Self-employed | -0.35 | -0.86 – 0.16 | 0.182 | 0.12 | -0.39 – 0.62 | 0.651 | -0.06 | -0.57 – 0.45 | 0.812 | 0.19 | -0.32 – 0.70 | 0.464 |
| Unemployed | -0.07 | -0.59 – 0.45 | 0.794 | 0.41 | -0.10 – 0.93 | 0.114 | 0.38 | -0.14 – 0.89 | 0.150 | -0.07 | -0.58 – 0.45 | 0.800 |
| Retired | -0.23 | -0.91 – 0.45 | 0.510 | -0.28 | -0.95 – 0.40 | 0.418 | -0.43 | -1.10 – 0.25 | 0.214 | 0.77 | 0.09 – 1.44 | **0.026** |
| Student | -0.02 | -1.03 – 1.00 | 0.973 | 0.15 | -0.86 – 1.15 | 0.774 | -0.37 | -1.38 – 0.64 | 0.469 | 0.09 | -0.92 – 1.10 | 0.866 |
| Employ. (Other) | -0.58 | -1.66 – 0.51 | 0.296 | -0.23 | -1.31 – 0.85 | 0.672 | -0.18 | -1.26 – 0.90 | 0.745 | 0.29 | -0.79 – 1.37 | 0.595 |
| Observations | 176 | | | 176 | | | 176 | | | 176 | | |

**Figure S1.** Distributions of outcome variables in Study 1a

| Openness  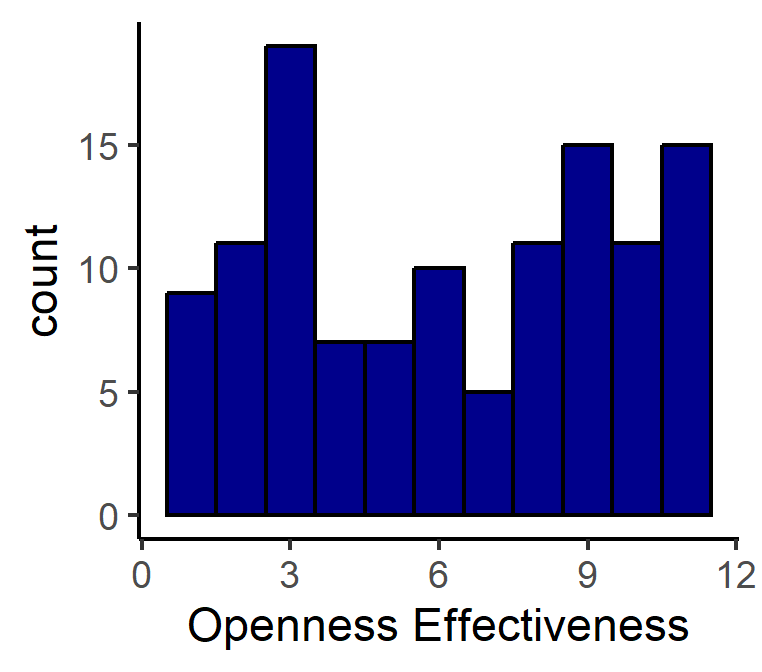 | Conscientiousness  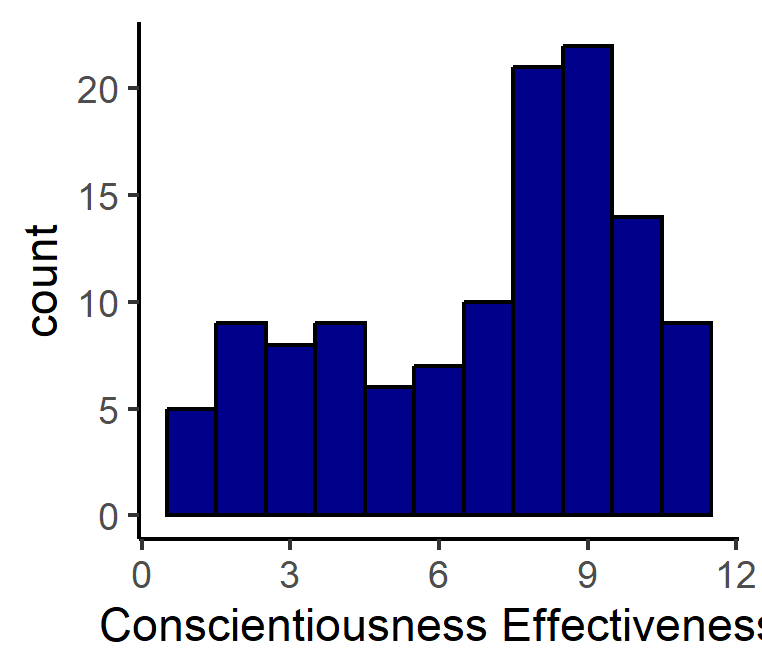 |
| --- | --- |
| Extraversion  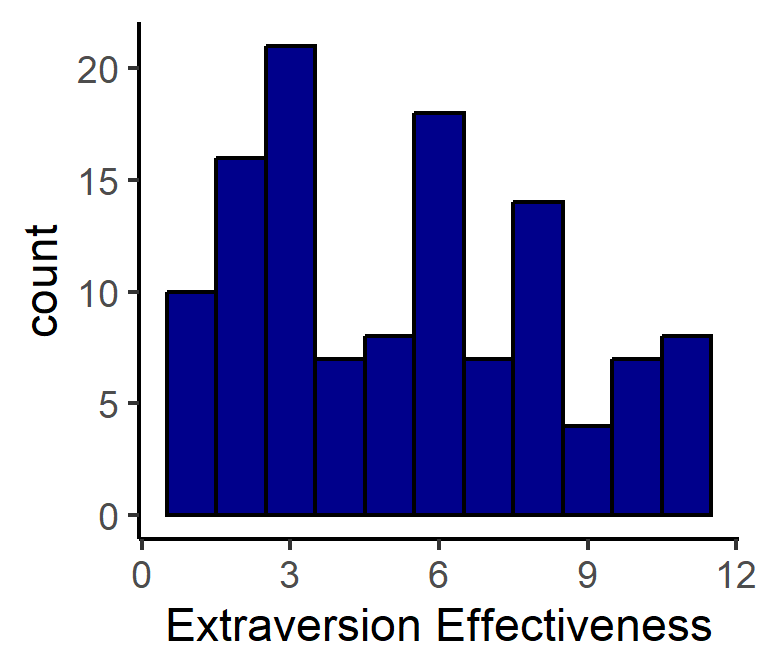 | Agreeableness  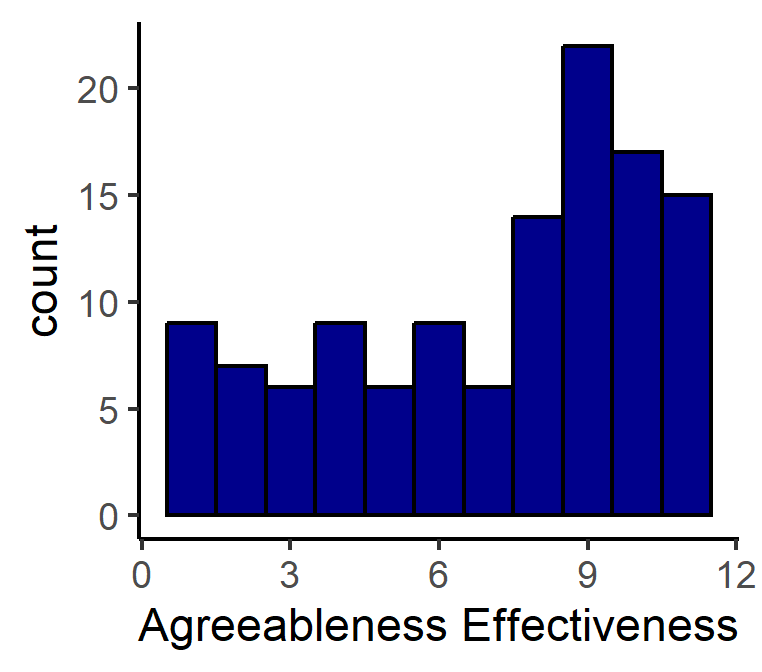 |

**Figure S2**. Distributions of outcome variables in Study 1b

| Openness  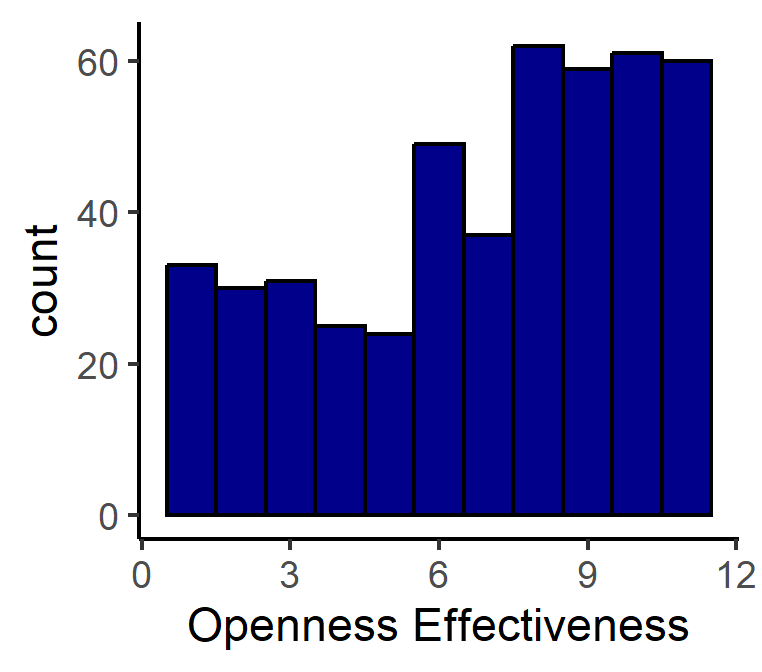 | Conscientiousness  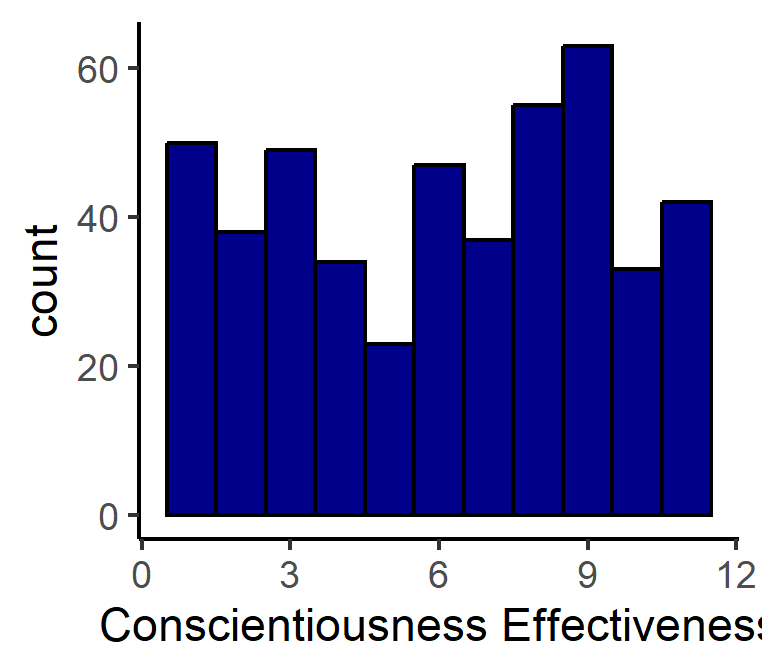 |
| --- | --- |
| Extraversion  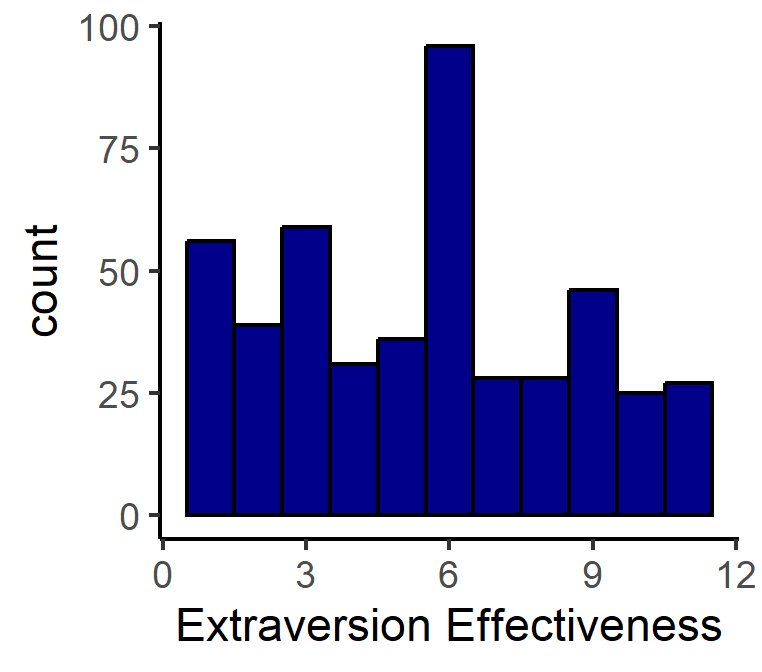 | Agreeableness  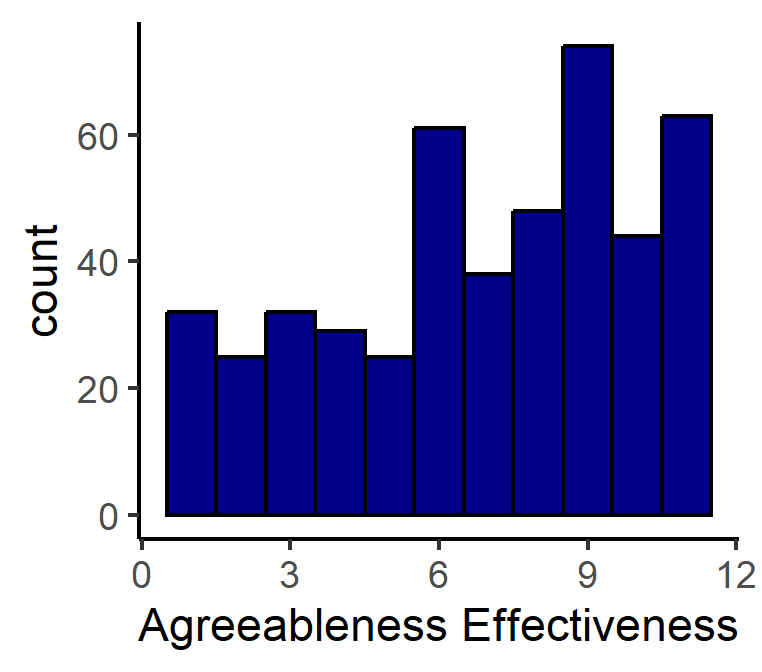 |

**Figure S3.** Marginal effects of Big Five personality traits on effectiveness ratings broken down by experimental condition.

*
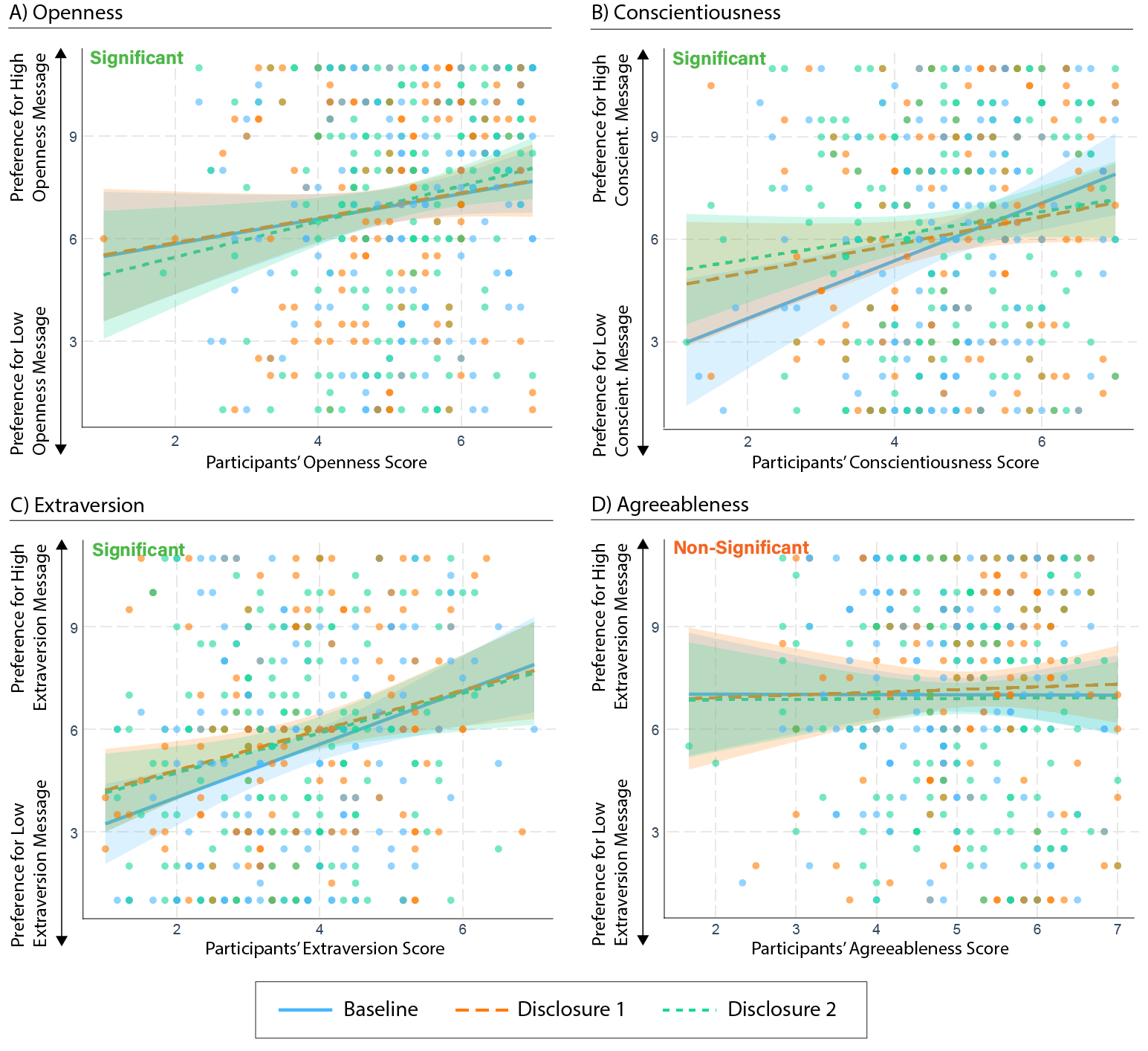
*

**Study 2**

**Sample demographics**

| Age | 36.7 (13.0SD) |
| --- | --- |
| Gender | 49% Female |
| Ethnicity | 70% White  13% Black or African American  1% American Indian, Native American or Alaska Native  8% Asian  0% Hawaiian Native or Other Pacific Islander  1% Other  7% Multiracial |
| Education | 2% Some high school or less  20% High school diploma or GED  25% Some college but no degree  10% Associates or technical degree  33% Bachelor’s degree  11% Graduate or professional degree (MA, MS, MBA, PHD, JD, MD etc.) |
| Income | 20% Less than $10,000  11% $10,000 to $14,999  7% $15,000 to $24,999  11% $25,000 to $34,999  13% $35,000 to $49,999  17% $50,000 to $74,999  10% $75,000 to $99,999  8% $100,000 to $149,999  1% $150,000 to $199,999  2% $200,000 or more |
| Employment | 42% Employed (full-time)  14% Employed (part-time)  10% Self-employed  18% Unemployed  10% Student  4% Retired  3% Other |

**Table S11.** ChatGPT Prompts and Materials for the Big Five Personality Traits

| **Trait** | **High** | **Low** |
| --- | --- | --- |
| OP | **Write a short Nike sneaker ad for someone who is open to new experience and artistic.**  Experience style and function like never before with the new Nike sneaker collection. Designed for those who value creativity and self-expression, our sneakers are more than just shoes – they're a canvas for your individuality. With bold and innovative designs, our sneakers are the perfect accessory to complete any look. Made with a focus on both style and comfort, our shoes are for those who want to look and feel their best. Our design team is made up of talented artists and creatives who are constantly pushing the boundaries of design and innovation. Try out our new Nike sneaker collection and elevate your style with the ultimate fusion of art and function. | **Write a short Nike sneaker ad for someone who is down-to-earth and traditional**  If you're someone who values practicality and simplicity, the new Nike sneaker collection is perfect for you. Our sneakers are designed with a focus on quality and durability, so you can trust that they will last through the wear and tear of everyday life. With classic and timeless designs, our sneakers are the perfect complement to any outfit. Made with a focus on both style and function, our shoes are for those who want to look and feel their best. Try out our new Nike sneaker collection and step into a world of practical, yet stylish footwear that will withstand the test of time. |
| CO | **Write a short Nike sneaker ad for someone who is dependable and organized.**  If you're someone who values reliability and organization, the new Nike sneaker collection is the perfect fit for you. Our sneakers are designed with a focus on quality and consistency, so you can trust that they will perform at their best in any situation. With clean and minimalistic designs, our sneakers are the perfect complement to any organized wardrobe. Made with a focus on both style and function, our shoes are for those who want to look and feel their best, while maintaining their dependable and organized nature. Try out our new Nike sneaker collection and step up your game with the ultimate fusion of reliability and style. | **Write a short Nike sneaker ad for someone who is disorganized and careless**  If you're someone who is always on the go and doesn't have time to worry about what you're wearing, the new Nike sneaker collection is perfect for you. Our sneakers are designed to keep up with your fast-paced lifestyle, while still looking stylish and on-trend. With vibrant and eye-catching designs, our sneakers will add a pop of color to any outfit, even if you're not sure what you're wearing yet. Made with a focus on both style and function, our shoes are for those who want to look and feel their best, without having to put in too much effort. Try out our new Nike sneaker collection and step into a world of effortless style that will keep up with your hectic lifestyle. |
| EX | **Write a short Nike sneaker ad for someone who is extraverted and enthusiastic.**  For those who thrive on energy and enthusiasm, the new Nike sneaker collection is the perfect match for your vibrant personality. Our sneakers are designed with bold and daring styles that will help you stand out from the crowd. With vibrant colors and eye-catching designs, our sneakers will keep up with your outgoing and adventurous nature. Made with a focus on both style and function, our shoes are for those who want to look and feel their best, while embracing their extroverted side. Try out our new Nike sneaker collection and take your enthusiasm to the next level with the ultimate fusion of energy and style. | **Write a short Nike sneaker ad for someone who is reserved and quiet**  If you're someone who values understated style and timeless designs, the new Nike sneaker collection is the perfect match for your reserved and quiet personality. Our sneakers are designed with clean lines and classic styles that will seamlessly complement any outfit. With a focus on simplicity and elegance, our sneakers will help you make a statement without saying a word. Made with a focus on both style and function, our shoes are for those who want to look and feel their best, while embracing their reserved and quiet nature. Try out our new Nike sneaker collection and discover the ultimate fusion of understated style and sophistication. |
| AG | **Write a short Nike sneaker ad for someone who is sympathetic and warm**  For those who value warmth and empathy, the new Nike sneaker collection is the perfect match for your sympathetic and caring personality. Our sneakers are designed with soft and comfortable materials that will keep your feet feeling cozy and supported. With a focus on comfort and style, our sneakers will keep you feeling your best while looking your best. Made with a focus on both style and function, our shoes are for those who want to look and feel their best, while embracing their caring and empathetic nature. Try out our new Nike sneaker collection and discover the ultimate fusion of comfort and style that will help you spread warmth wherever you go. | **Write a short Nike sneaker ad for someone who is critical and competitive**  For those who value competition and performance, the new Nike sneaker collection is the perfect match for your critical and competitive personality. Our sneakers are designed with cutting-edge materials and advanced technology that will take your athletic performance to the next level. With a focus on durability and performance, our sneakers will help you push your limits and achieve your goals. Made with a focus on both style and function, our shoes are for those who want to look and feel their best, while embracing their competitive and critical nature. Try out our new Nike sneaker collection and discover the ultimate fusion of high-performance technology and sleek style. |

**Table S12.** ChatGPT Prompts and Materials for Regulatory Focus

| **Prevention Focus** | **Promotion Focus** |
| --- | --- |
| **Write a short persuasive ad to convince a prevention-focused person to exercise more.**  Are you the type of person who loves setting goals and achieving them? Do you thrive on the thrill of success and the rewards that come with it? If so, then I have a proposal for you: start exercising more.  Not only is regular exercise good for your physical health, but it can also be a powerful tool to help you achieve your goals. Think about it – when you exercise, you're pushing yourself to improve and become stronger. You're setting a goal for yourself and working hard to achieve it.  And the best part? When you exercise regularly, you'll start to see results. You'll feel better, look better, and have more energy. Plus, as you become more fit, you'll be able to tackle other challenges with ease.  So if you're someone who loves a good challenge and wants to achieve big things, start incorporating exercise into your routine. Set a goal for yourself – whether it's running a 5K or mastering a new yoga pose – and work hard to make it happen. You'll be amazed at what you can achieve when you put your mind to it. | **Write a short persuasive ad to convince a promotion-focused person to exercise more.**  Do you value the importance of maintaining good health and preventing future health problems? Do you prioritize taking care of yourself and avoiding potential risks to your well-being? If so, then I have a suggestion for you: start exercising more.  Regular exercise is one of the best things you can do to prevent a wide range of health issues, from heart disease and diabetes to depression and anxiety. By incorporating physical activity into your daily routine, you'll be taking a proactive step towards protecting your long-term health and reducing your risk of developing serious health conditions.  In addition, exercise has been shown to boost the immune system, improve sleep quality, and reduce stress levels. These benefits can help you feel better both physically and mentally, and set you up for a happier, healthier future.  So if you're someone who values prevention and wants to take control of your health, start making exercise a priority. It doesn't have to be complicated or time-consuming – even a brisk walk or a short yoga session can make a difference. By taking care of your body now, you'll be giving yourself the best chance for a healthy and vibrant life in the years to come. |

**Table S13.** ChatGPT Prompts and Materials for the Moral Foundations

| **Foundation** | **Message** |
| --- | --- |
| Purity | **Write a short political ad in favor of taking climate action that is tailored to people scoring high on the moral foundation of purity.**  Do you believe in protecting our planet and preserving the natural world for future generations? Do you believe in keeping our air and water clean, and ensuring that our environment is free from pollution and contamination? If so, then I urge you to consider the benefits of taking action on climate change.  Climate change is not just an abstract concept or a far-off threat – it's a real and pressing danger to the purity and health of our planet. Rising temperatures, extreme weather events, and sea level rise are all symptoms of a global problem that requires urgent action.  By taking action on climate change, we can help to protect the purity and integrity of our environment, preserving the natural world for future generations. We can reduce our carbon emissions, promote renewable energy sources, and work to create a more sustainable and resilient economy.  Moreover, taking action on climate change is not just the right thing to do – it's also an opportunity to create jobs, stimulate innovation, and build a stronger and more vibrant economy. By investing in clean energy and sustainable infrastructure, we can create new opportunities for growth and prosperity while also protecting the purity and health of our planet.  So if you believe in the importance of keeping our planet clean and pure, we urge you to support action on climate change. Together, we can create a future that is both sustainable and prosperous, reflecting the best of our shared values and commitments to the purity and health of our planet. |
| Care | **Write a short political ad in favor of taking climate action that is tailored to people scoring high on the moral foundation of care.**  Do you believe in taking care of the planet and protecting the natural world for future generations? Do you feel a strong sense of responsibility to the people and creatures that share our world with us? If so, then I urge you to consider the benefits of taking action on climate change.  Climate change is not just an environmental issue – it's a moral imperative to care for the planet and all its inhabitants. From the smallest creatures to the largest ecosystems, the impacts of climate change are being felt by all, and it is our duty to take action to mitigate the damage.  By taking action on climate change, we can help to care for the planet and protect the natural world for future generations. We can invest in renewable energy sources, promote sustainable agriculture, and work to create a more equitable and just society that prioritizes the needs of all beings.  Moreover, taking action on climate change is not just a matter of caring for the planet – it's also an opportunity to lead by example and inspire others to do the same. By showing our care for the planet and taking steps to protect it, we can help to create a brighter and more hopeful future for all.  So if you believe in taking care of the planet and protecting the natural world, we urge you to support action on climate change. Together, we can create a future that is both sustainable and just, reflecting the best of our shared values and commitments to the planet and all its inhabitants. |
| Loyalty | **Write a short political ad in favor of taking climate action that is tailored to people scoring high on the moral foundation of loyalty.**  Do you believe in standing up for the people and places that you love? Do you feel a strong sense of loyalty to your community, your country, and the natural world around you? If so, then I urge you to consider the benefits of taking action on climate change.  Climate change is not just an environmental issue – it's a threat to the places and people that we hold dear. From the rising sea levels that threaten our coastal communities to the extreme weather events that disrupt our lives and livelihoods, the impacts of climate change are being felt all around us.  By taking action on climate change, we can help to protect the people and places that we love, showing our loyalty to our communities and our country. We can invest in resilient infrastructure, prepare for the impacts of climate change, and work to build a stronger and more secure future for all.  Moreover, taking action on climate change is not just a matter of protecting our own interests – it's also an opportunity to lead by example and inspire others to do the same. By showing our loyalty to the natural world and taking steps to protect it, we can help to create a brighter and more hopeful future for all.  So if you believe in standing up for the people and places that you love, we urge you to support action on climate change. Together, we can create a future that is both loyal and resilient, reflecting the best of our shared values and commitments to the people and places that we call home. |
| Fairness | **Write a short political ad in favor of taking climate action that is tailored to people scoring high on the moral foundation of fairness.**  Do you believe in creating a fair and just world for all? Do you feel a strong sense of responsibility to ensure that everyone has access to clean air, clean water, and a healthy environment? If so, then I urge you to consider the benefits of taking action on climate change.  Climate change is not just an environmental issue – it's a matter of fairness and justice. The impacts of climate change are being felt most acutely by the most vulnerable members of society, including low-income communities and people of color. These communities often lack the resources and political power to protect themselves from the effects of climate change.  By taking action on climate change, we can help to create a fairer and more just world for all. We can invest in renewable energy sources, promote sustainable transportation, and work to create a more equitable and inclusive society that prioritizes the needs of all people.  Moreover, taking action on climate change is not just a matter of fairness – it's also an opportunity to lead by example and inspire others to do the same. By showing our commitment to fairness and justice, we can help to create a brighter and more hopeful future for all.  So if you believe in creating a fair and just world for all, we urge you to support action on climate change. Together, we can create a future that is both sustainable and equitable, reflecting the best of our shared values and commitments to fairness and justice for all. |
| Authority | **Write a short political ad in favor of taking climate action that is tailored to people scoring high on the moral foundation of authority.**  Do you believe in the importance of responsible leadership and making decisions based on expert knowledge? Do you feel a strong sense of obligation to follow the guidance of respected authorities and scientific consensus? If so, then I urge you to consider the benefits of taking action on climate change.  Climate change is not just an environmental issue – it's a matter of following the guidance of respected authorities and scientific consensus. The overwhelming majority of climate scientists agree that the Earth is experiencing significant changes due to human activity, and that action is needed to mitigate the worst effects of climate change.  By taking action on climate change, we can show our commitment to responsible leadership and following the guidance of respected authorities. We can invest in renewable energy sources, reduce our carbon footprint, and work to create a more sustainable and resilient society that reflects the latest scientific knowledge.  Moreover, taking action on climate change is not just a matter of following the guidance of respected authorities – it's also an opportunity to lead by example and inspire others to do the same. By showing our commitment to responsible leadership, we can help to create a brighter and more hopeful future for all.  So if you believe in the importance of responsible leadership and following the guidance of respected authorities, we urge you to support action on climate change. Together, we can create a future that is both sustainable and reflective of the best of our shared values and commitments to science and responsible leadership. |

**Stimulus validation**

To ensure that the stimuli displayed in Table S11-S13 reflect the psychological traits they were meant to target, we ran a small pre-validation study. Specifically, we recruited 20 undergraduate and graduate students (the same students used to validate the stimuli for Study 1) to read the messages and indicate which personality trait they believed the message was tailored to.

1. ***Sneaker ads tailored to Big Five personality traits***

***“****On the following pages we will show you ads for* ***Nike sneakers****and ask you for your reaction. The ads have been designed to appeal to people with different****personality traits****. Please use the ratings scales to indicate which characteristics you believe the ads reflect the most.*

*[AD]*

*Which personality characteristic does this ad reflect the most?”*

*
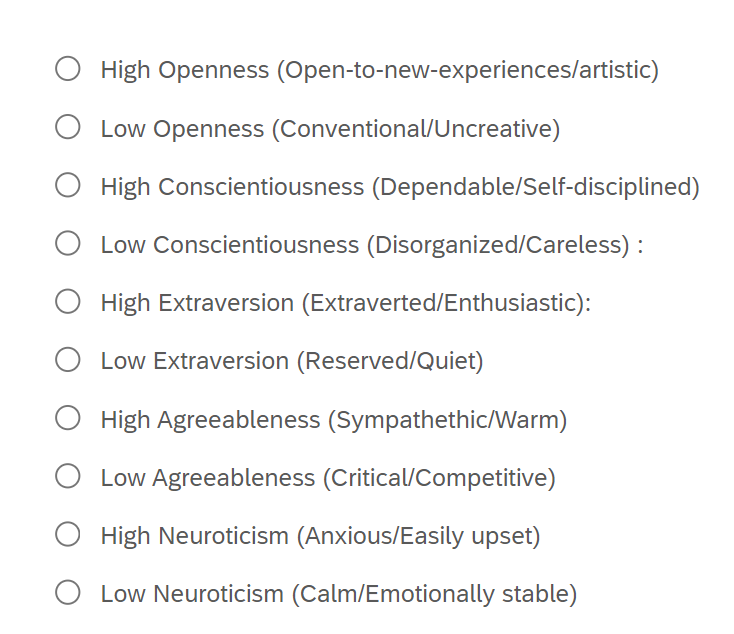
*

Given the ten different response options, the baseline likelihood of participants guessing the correct answer is 10%. Table S14 below shows the actual percentages for each of the eight messages.

**Table S14.** Percentage of participants correctly identifying the targeted trait.

|  | Percentage correct | |
| --- | --- | --- |
| Trait | High | Low |
| OP | 20/20 (100%) | 10/20 (50%) |
| CO | 20/20 (100%) | 8/20 (40%) |
| EX | 20/20 (100%) | 16/20 (80%) |
| AG | 20/20 (100%) | 20/20 (100%) |

1. ***Short political speeches tailored to the Moral Foundations***

***“****On the following pages we will show you short****political speeches advocating for climate action.****The speeches have been designed to appeal to people of different****moral values****. Please use the ratings scales to indicate which values you believe the speeches reflect the most.*

*[SPEECH]*

*Which moral foundation does this speech reflect most?”*

***
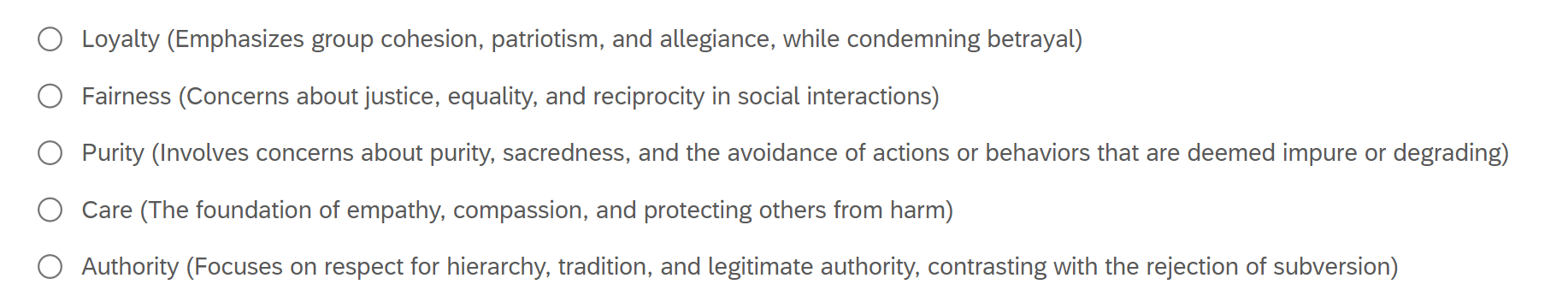
***

Given the five different response options, the baseline likelihood of participants guessing the correct answer is 20%. Table S15 below shows the actual percentages for each of the eight messages.

**Table S15.** Percentage of participants correctly identifying the targeted trait

| Trait | Percentage correct |
| --- | --- |
| Loyalty | 16/20 (80%) |
| Fairness | 20/20 (100%) |
| Purity | 20/20 (100%) |
| Care | 16/20 (80%) |
| Authority | 20/20 (100%) |

1. ***Health messages tailored to regulatory focus***

*“On the following pages we will show you short persuasive messages trying to****convince people to exercise more****. The messages have been designed to appeal to people with different****motivational orientations (promotion versus prevention focus)****. Please use the ratings scales to indicate which motivational orientation you believe the messages reflect the most.*

*[MESSAGE]*

*Which motivational orientation does this message reflect most?”*


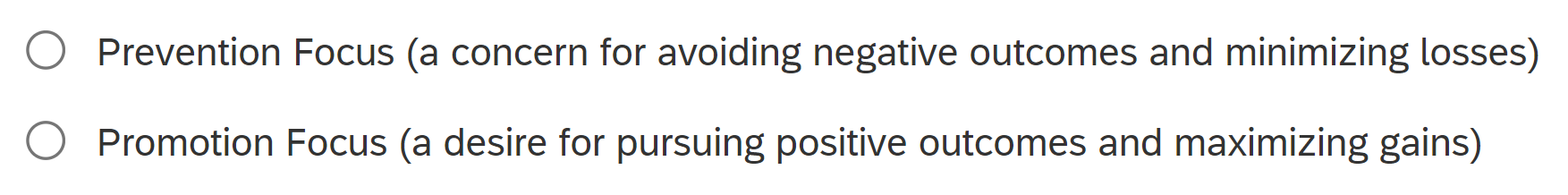


Given the two different response options, the baseline likelihood of participants guessing the correct answer is 50%. Table S16 below shows the actual percentages for each of the eight messages.

**Table S16.** Percentage of participants correctly identifying the targeted trait

| Trait | Percentage correct |
| --- | --- |
| Promotion | 20/20 (100%) |
| Prevention | 20/20 (100%) |

**Table S17.** *Means, standard deviations, and zero-order correlations with confidence intervals of Big Five Personality variables in Study 2*

| Variable | *M* | *SD* | 1 | 2 | 3 | 4 | 5 | 6 | 7 | 8 | 9 | 10 |
| --- | --- | --- | --- | --- | --- | --- | --- | --- | --- | --- | --- | --- |
|  |  |  |  |  |  |  |  |  |  |  |  |  |
| 1. Openness (Ad) | 6.14 | 3.40 |  |  |  |  |  |  |  |  |  |  |
|  |  |  |  |  |  |  |  |  |  |  |  |  |
| 2. Conscient. (Ad) | 5.56 | 3.40 | -.13 |  |  |  |  |  |  |  |  |  |
|  |  |  | [-.27, .01] |  |  |  |  |  |  |  |  |  |
|  |  |  |  |  |  |  |  |  |  |  |  |  |
| 3. Extraversion (Ad) | 5.64 | 3.32 | .39** | -.23** |  |  |  |  |  |  |  |  |
|  |  |  | [.26, .50] | [-.36, -.09] |  |  |  |  |  |  |  |  |
|  |  |  |  |  |  |  |  |  |  |  |  |  |
| 4. Agreeableness (Ad) | 5.02 | 3.22 | -.24** | -.01 | -.30** |  |  |  |  |  |  |  |
|  |  |  | [-.37, -.10] | [-.15, .13] | [-.43, -.17] |  |  |  |  |  |  |  |
|  |  |  |  |  |  |  |  |  |  |  |  |  |
| 5. Openness (P) | 5.29 | 1.11 | .21** | .06 | .14* | .01 |  |  |  |  |  |  |
|  |  |  | [.07, .34] | [-.09, .20] | [.00, .28] | [-.14, .15] |  |  |  |  |  |  |
|  |  |  |  |  |  |  |  |  |  |  |  |  |
| 6. Conscient. (P) | 4.53 | 1.33 | -.07 | .05 | -.07 | .01 | .06 |  |  |  |  |  |
|  |  |  | [-.21, .07] | [-.09, .19] | [-.21, .07] | [-.13, .16] | [-.08, .20] |  |  |  |  |  |
|  |  |  |  |  |  |  |  |  |  |  |  |  |
| 7. Extraversion (P) | 3.68 | 1.22 | .00 | -.01 | .12 | -.01 | .18* | .45** |  |  |  |  |
|  |  |  | [-.14, .14] | [-.15, .13] | [-.02, .26] | [-.15, .14] | [.04, .32] | [.33, .55] |  |  |  |  |
|  |  |  |  |  |  |  |  |  |  |  |  |  |
| 8. Agreeableness (P) | 5.08 | 1.02 | -.00 | -.02 | .02 | .03 | .08 | .44** | .19** |  |  |  |
|  |  |  | [-.14, .14] | [-.16, .13] | [-.13, .16] | [-.12, .17] | [-.06, .22] | [.32, .55] | [.05, .32] |  |  |  |
|  |  |  |  |  |  |  |  |  |  |  |  |  |
| 9. Neuroticism (P) | 3.88 | 1.43 | -.04 | -.09 | .03 | -.02 | .01 | -.61** | -.50** | -.42** |  |  |
|  |  |  | [-.18, .11] | [-.22, .06] | [-.12, .17] | [-.16, .12] | [-.13, .15] | [-.69, -.51] | [-.60, -.39] | [-.53, -.30] |  |  |
|  |  |  |  |  |  |  |  |  |  |  |  |  |
| 10. Age | 36.74 | 12.98 | -.05 | -.12 | -.10 | .06 | -.08 | .21** | .11 | .13 | -.22** |  |
|  |  |  | [-.19, .09] | [-.26, .02] | [-.24, .05] | [-.08, .20] | [-.22, .06] | [.07, .34] | [-.03, .25] | [-.02, .26] | [-.35, -.08] |  |
|  |  |  |  |  |  |  |  |  |  |  |  |  |
| 11. Education | 3.86 | 1.39 | .07 | .05 | .03 | -.02 | .06 | .17* | .16* | .09 | -.17* | .10 |
|  |  |  | [-.07, .21] | [-.09, .19] | [-.11, .17] | [-.16, .12] | [-.09, .20] | [.03, .31] | [.02, .30] | [-.06, .23] | [-.30, -.03] | [-.04, .24] |
|  |  |  |  |  |  |  |  |  |  |  |  |  |

*Note.* *M* and *SD* are used to represent mean and standard deviation, respectively. Values in square brackets indicate the 95% confidence interval for each correlation. (Ad) indicates effectiveness ratings for the respective ads. (P) indicates participants’ personality scores on the respective trait. * indicates *p* < .05. ** indicates *p* < .01.

**Table S18.** Full model output of linear regression analyses for Big Five Personality Traits in Study 2

|  | **Openness** | | | **Conscientiousness** | | | **Extraversion** | | | **Agreeableness** | | |
| --- | --- | --- | --- | --- | --- | --- | --- | --- | --- | --- | --- | --- |
| *Predictors* | *β* | *CI_95_* | *p* | *β* | *CI_95_* | *p* | *β* | *CI_95_* | *p* | *β* | *CI_95_* | *p* |
| (Intercept) | -0.17 | -0.46 – 0.13 | **0.041** | 0.05 | -0.26 – 0.35 | **0.004** | -0.25 | -0.55 – 0.06 | 0.434 | -0.06 | -0.37 – 0.25 | **0.035** |
| Openness | 0.19 | 0.04 – 0.34 | **0.012** | 0.06 | -0.09 – 0.22 | 0.425 | 0.12 | -0.04 – 0.27 | 0.141 | 0.03 | -0.13 – 0.19 | 0.685 |
| Conscientious. | -0.20 | -0.40 – -0.01 | **0.039** | 0.08 | -0.12 – 0.28 | 0.424 | -0.15 | -0.35 – 0.05 | 0.149 | -0.02 | -0.22 – 0.18 | 0.847 |
| Extraversion | -0.07 | -0.25 – 0.10 | 0.405 | -0.15 | -0.33 – 0.03 | 0.095 | 0.19 | 0.01 – 0.37 | **0.040** | -0.03 | -0.22 – 0.15 | 0.718 |
| Agreeableness | 0.05 | -0.12 – 0.21 | 0.580 | -0.04 | -0.21 – 0.13 | 0.664 | 0.06 | -0.11 – 0.24 | 0.461 | -0.00 | -0.18 – 0.17 | 0.984 |
| Neuroticism | -0.18 | -0.39 – 0.02 | 0.077 | -0.16 | -0.37 – 0.05 | 0.139 | 0.01 | -0.20 – 0.22 | 0.950 | -0.08 | -0.30 – 0.13 | 0.436 |
| Age | -0.05 | -0.22 – 0.13 | 0.605 | -0.12 | -0.31 – 0.06 | 0.184 | -0.13 | -0.32 – 0.05 | 0.146 | -0.02 | -0.20 – 0.17 | 0.833 |
| Female | -0.01 | -0.32 – 0.29 | 0.924 | -0.21 | -0.53 – 0.10 | 0.180 | 0.13 | -0.18 – 0.45 | 0.400 | 0.31 | -0.01 – 0.63 | 0.057 |
| Gender (Other) | 0.13 | -1.29 – 1.56 | 0.854 | 0.59 | -0.88 – 2.06 | 0.430 | 0.19 | -1.28 – 1.66 | 0.800 | 0.52 | -0.97 – 2.01 | 0.488 |
| Education | 0.13 | -0.03 – 0.29 | 0.118 | 0.04 | -0.13 – 0.21 | 0.667 | 0.08 | -0.09 – 0.25 | 0.370 | 0.00 | -0.17 – 0.18 | 0.967 |
| Black | 0.64 | 0.21 – 1.07 | **0.004** | 0.15 | -0.29 – 0.60 | 0.500 | 0.03 | -0.42 – 0.47 | 0.908 | -0.08 | -0.53 – 0.37 | 0.725 |
| Native American | -1.58 | -3.05 – -0.12 | **0.034** | -0.66 | -2.17 – 0.85 | 0.390 | 0.65 | -0.86 – 2.16 | 0.395 | -1.24 | -2.78 – 0.29 | 0.111 |
| Asian | -0.12 | -0.67 – 0.43 | 0.673 | 0.10 | -0.47 – 0.66 | 0.741 | 0.21 | -0.35 – 0.78 | 0.457 | -0.41 | -0.99 – 0.16 | 0.157 |
| Pacific Islander | -0.68 | -2.09 – 0.73 | 0.345 | 1.11 | -0.35 – 2.57 | 0.134 | 0.52 | -0.93 – 1.98 | 0.478 | 0.01 | -1.46 – 1.49 | 0.986 |
| Multi-racial | 0.48 | -0.11 – 1.08 | 0.110 | -0.32 | -0.94 – 0.29 | 0.301 | -0.10 | -0.71 – 0.52 | 0.753 | -0.07 | -0.69 – 0.55 | 0.830 |
| Part-time | 0.24 | -0.22 – 0.70 | 0.311 | 0.50 | 0.02 – 0.98 | **0.041** | 0.32 | -0.15 – 0.80 | 0.181 | -0.35 | -0.83 – 0.14 | 0.157 |
| Self-employed | 0.13 | -0.42 – 0.67 | 0.650 | -0.04 | -0.61 – 0.52 | 0.876 | 0.07 | -0.50 – 0.63 | 0.816 | -0.10 | -0.67 – 0.47 | 0.722 |
| Unemployed | -0.06 | -0.51 – 0.40 | 0.810 | -0.10 | -0.57 – 0.37 | 0.667 | 0.07 | -0.40 – 0.54 | 0.768 | 0.21 | -0.26 – 0.69 | 0.384 |
| Retired | 0.27 | -0.31 – 0.84 | 0.364 | 0.01 | -0.58 – 0.61 | 0.960 | 0.49 | -0.10 – 1.09 | 0.102 | -0.35 | -0.95 – 0.25 | 0.253 |
| Student | 0.37 | -0.54 – 1.27 | 0.427 | -0.19 | -1.13 – 0.75 | 0.686 | 0.80 | -0.14 – 1.74 | 0.094 | 0.53 | -0.43 – 1.48 | 0.277 |
| Employ. (Other) | 0.63 | -0.34 – 1.60 | 0.201 | 0.04 | -0.96 – 1.04 | 0.936 | 0.38 | -0.62 – 1.38 | 0.455 | -0.03 | -1.05 – 0.98 | 0.949 |
| Observations | 192 | | | 192 | | | 192 | | | 192 | | |
| R^2^ / R^2^ adjusted | 0.172 / 0.075 | | | 0.117 / 0.014 | | | 0.119 / 0.016 | | | 0.092 / -0.014 | | |

**Table S19.** *Means, standard deviations, and zero-order correlations with confidence intervals of Regulatory Focus variables in Study 2*

| Variable | *M* | *SD* | 1 | 2 | 3 |
| --- | --- | --- | --- | --- | --- |
|  |  |  |  |  |  |
| 1. Regulatory Focus (Ad) | 7.40 | 3.28 |  |  |  |
|  |  |  |  |  |  |
| 2. Regulatory Focus (P) | 1.01 | 2.03 | .09 |  |  |
|  |  |  | [-.05, .23] |  |  |
|  |  |  |  |  |  |
| 3. Age | 36.74 | 12.98 | .19** | .11 |  |
|  |  |  | [.05, .32] | [-.03, .25] |  |
|  |  |  |  |  |  |
| 4. Education | 3.86 | 1.39 | .01 | .20** | .10 |
|  |  |  | [-.13, .15] | [.06, .33] | [-.04, .24] |

*Note.* *M* and *SD* are used to represent mean and standard deviation, respectively. Values in square brackets indicate the 95% confidence interval for each correlation. (Ad) indicates effectiveness ratings for the respective ads. (P) indicates participants’ regulatory focus score. * indicates *p* < .05. ** indicates *p* < .01.

**Table S20.** Full model output of linear regression analyses for Regulatory Focus in Study 2

|  | **Promotion Focus** | | |
| --- | --- | --- | --- |
| *Predictors* | *β* | *CI_95_* | *p* |
| (Intercept) | -0.03 | -0.32 – 0.25 | **<0.001** |
| Promotion  (- Prevention) | 0.12 | -0.03 – 0.27 | 0.125 |
| Age | 0.13 | -0.05 – 0.30 | 0.150 |
| Female | 0.20 | -0.10 – 0.49 | 0.186 |
| Gender (Other) | 0.61 | -0.82 – 2.03 | 0.401 |
| Education | -0.02 | -0.19 – 0.15 | 0.822 |
| Black | -0.40 | -0.83 – 0.03 | 0.069 |
| Native American | 0.57 | -0.88 – 2.02 | 0.438 |
| Asian | -0.07 | -0.63 – 0.49 | 0.808 |
| Pacific Islander | -1.96 | -3.36 – -0.57 | **0.006** |
| Multi-racial | -0.56 | -1.15 – 0.03 | 0.064 |
| Part-time | 0.14 | -0.31 – 0.60 | 0.533 |
| Self-employed | -0.06 | -0.59 – 0.48 | 0.833 |
| Unemployed | 0.06 | -0.38 – 0.49 | 0.804 |
| Retired | -0.00 | -0.58 – 0.57 | 0.998 |
| Student | 0.09 | -0.82 – 1.00 | 0.839 |
| Employ. (Other) | 0.39 | -0.56 – 1.33 | 0.421 |
| Observations | 192 | | |
| R^2^ / R^2^ adjusted | 0.135 / 0.056 | | |

**Table S21.** *Means, standard deviations, and zero-order correlations with 95% CI of Moral Foundations in Study 2*

| Variable | *M* | *SD* | 1 | 2 | 3 | 4 | 5 | 6 | 7 | 8 | 9 | 10 | 11 | 12 | 13 |
| --- | --- | --- | --- | --- | --- | --- | --- | --- | --- | --- | --- | --- | --- | --- | --- |
|  |  |  |  |  |  |  |  |  |  |  |  |  |  |  |  |
| 1. Pol. Ideol (Ad) | 40.14 | 17.16 |  |  |  |  |  |  |  |  |  |  |  |  |  |
|  |  |  |  |  |  |  |  |  |  |  |  |  |  |  |  |
| 2. Purity (Ad) | 26.12 | 15.96 | -.39** |  |  |  |  |  |  |  |  |  |  |  |  |
|  |  |  | [-.51, -.27] |  |  |  |  |  |  |  |  |  |  |  |  |
|  |  |  |  |  |  |  |  |  |  |  |  |  |  |  |  |
| 3. Care (Ad) | 21.97 | 13.59 | .64** | -.17* |  |  |  |  |  |  |  |  |  |  |  |
|  |  |  | [.54, .71] | [-.30, -.03] |  |  |  |  |  |  |  |  |  |  |  |
|  |  |  |  |  |  |  |  |  |  |  |  |  |  |  |  |
| 4. Loyalty (Ad) | 20.62 | 13.43 | -.43** | -.32** | -.30** |  |  |  |  |  |  |  |  |  |  |
|  |  |  | [-.54, -.31] | [-.44, -.18] | [-.42, -.16] |  |  |  |  |  |  |  |  |  |  |
|  |  |  |  |  |  |  |  |  |  |  |  |  |  |  |  |
| 5. Fairness (Ad) | 18.17 | 13.52 | .63** | -.33** | -.20** | -.25** |  |  |  |  |  |  |  |  |  |
|  |  |  | [.54, .71] | [-.45, -.20] | [-.33, -.06] | [-.38, -.11] |  |  |  |  |  |  |  |  |  |
|  |  |  |  |  |  |  |  |  |  |  |  |  |  |  |  |
| 6. Authority (Ad) | 13.12 | 13.43 | -.38** | -.37** | -.32** | -.07 | -.17* |  |  |  |  |  |  |  |  |
|  |  |  | [-.50, -.25] | [-.49, -.24] | [-.44, -.18] | [-.21, .07] | [-.30, -.03] |  |  |  |  |  |  |  |  |
|  |  |  |  |  |  |  |  |  |  |  |  |  |  |  |  |
| 7. Pol. Ideol. (P) | 5.08 | 1.62 | .24** | -.01 | .07 | -.16* | .23** | -.13 |  |  |  |  |  |  |  |
|  |  |  | [.10, .37] | [-.15, .13] | [-.08, .21] | [-.30, -.02] | [.10, .36] | [-.27, .01] |  |  |  |  |  |  |  |
|  |  |  |  |  |  |  |  |  |  |  |  |  |  |  |  |
| 8. Purity (P) | 3.21 | 1.26 | -.11 | -.05 | .02 | .05 | -.16* | .15* | -.37** |  |  |  |  |  |  |
|  |  |  | [-.25, .03] | [-.19, .09] | [-.12, .16] | [-.09, .19] | [-.30, -.02] | [.00, .28] | [-.49, -.24] |  |  |  |  |  |  |
|  |  |  |  |  |  |  |  |  |  |  |  |  |  |  |  |
| 9. Care (P) | 4.67 | 0.79 | .16* | .08 | .10 | -.10 | .10 | -.20** | .31** | .18* |  |  |  |  |  |
|  |  |  | [.02, .29] | [-.07, .22] | [-.05, .24] | [-.23, .05] | [-.04, .24] | [-.33, -.06] | [.18, .43] | [.04, .31] |  |  |  |  |  |
|  |  |  |  |  |  |  |  |  |  |  |  |  |  |  |  |
| 10. Loyalty (P) | 3.07 | 0.97 | -.18* | -.08 | -.04 | .13 | -.19** | .20** | -.29** | .58** | .10 |  |  |  |  |
|  |  |  | [-.32, -.04] | [-.22, .06] | [-.18, .10] | [-.01, .27] | [-.32, -.05] | [.06, .33] | [-.41, -.15] | [.48, .67] | [-.04, .24] |  |  |  |  |
|  |  |  |  |  |  |  |  |  |  |  |  |  |  |  |  |
| 11. Fairness (P) | 4.58 | 0.71 | .30** | .04 | .10 | -.18* | .28** | -.25** | .47** | -.04 | .67** | -.14* |  |  |  |
|  |  |  | [.16, .42] | [-.10, .18] | [-.04, .24] | [-.31, -.04] | [.14, .40] | [-.38, -.11] | [.35, .57] | [-.18, .10] | [.58, .74] | [-.28, -.00] |  |  |  |
|  |  |  |  |  |  |  |  |  |  |  |  |  |  |  |  |
| 12. Authority (P) | 3.38 | 1.00 | -.18* | -.05 | -.01 | .11 | -.21** | .18* | -.44** | .75** | .03 | .70** | -.17* |  |  |
|  |  |  | [-.31, -.03] | [-.19, .09] | [-.15, .13] | [-.03, .25] | [-.34, -.07] | [.04, .31] | [-.55, -.32] | [.68, .81] | [-.11, .17] | [.62, .77] | [-.30, -.03] |  |  |
|  |  |  |  |  |  |  |  |  |  |  |  |  |  |  |  |
| 13. Age | 36.74 | 12.98 | .01 | .04 | .14 | -.03 | -.13 | -.03 | -.07 | .19* | .10 | .26** | -.03 | .22** |  |
|  |  |  | [-.14, .15] | [-.10, .18] | [-.01, .27] | [-.17, .12] | [-.27, .01] | [-.17, .11] | [-.21, .07] | [.05, .32] | [-.04, .24] | [.12, .39] | [-.17, .11] | [.08, .35] |  |
|  |  |  |  |  |  |  |  |  |  |  |  |  |  |  |  |
| 14. Education | 3.86 | 1.39 | .13 | -.04 | .15* | -.02 | .00 | -.09 | .15* | .03 | .08 | .06 | .07 | .04 | .10 |
|  |  |  | [-.02, .26] | [-.18, .10] | [.01, .29] | [-.16, .12] | [-.14, .15] | [-.23, .06] | [.00, .28] | [-.11, .17] | [-.06, .22] | [-.08, .20] | [-.07, .21] | [-.10, .19] | [-.04, .24] |
|  |  |  |  |  |  |  |  |  |  |  |  |  |  |  |  |

**Table S22.** Full model output of linear regression analyses for Moral Foundations in Study 2

|  | **Purity** | | | **Care** | | | **Loyalty** | | | **Fairness** | | | **Authority** | | | **Liberalism** | | |
| --- | --- | --- | --- | --- | --- | --- | --- | --- | --- | --- | --- | --- | --- | --- | --- | --- | --- | --- |
| *Predictors* | *β* | *CI_95_* | *p* | *β* | *CI_95_* | *p* | *β* | *CI_95_* | *p* | *β* | *CI_95_* | *p* | *β* | *CI_95_* | *p* | *β* | *CI_95_* | *p* |
| (Intercept) | -0.01 | -0.31 – 0.29 | **<0.001** | -0.01 | -0.30 – 0.29 | 0.449 | -0.26 | -0.55 – 0.03 | **0.010** | -0.05 | -0.34 – 0.23 | 0.648 | 0.46 | 0.17 – 0.74 | **0.002** | -0.11 | -0.40 – 0.17 | **0.002** |
| Purity | -0.04 | -0.20 – 0.12 | 0.645 | - | - | - | - | - | - | - | - | - | - | - | - | - | - | - |
| Care | - | - | - | 0.06 | -0.10 – 0.21 | 0.468 | - | - | - | - | - | - | - | - | - | - | - | - |
| Loyalty | - | - | - | - | - | - | 0.17 | 0.02 – 0.32 | **0.026** | - | - | - | - | - | - | - | - | - |
| Fairness | - | - | - | - | - | - | - | - | - | 0.25 | 0.11 – 0.40 | **0.001** | - | - | - | - | - | - |
| Authority | - | - | - | - | - | - | - | - | - | - | - | - | 0.22 | 0.07 – 0.37 | **0.005** | - | - | - |
| Liberalism | - | - | - | - | - | - | - | - | - | - | - | - | - | - | - | 0.22 | 0.08 – 0.37 | **0.003** |
| Age | 0.09 | -0.10 – 0.27 | 0.355 | 0.16 | -0.02 – 0.34 | 0.078 | -0.13 | -0.31 – 0.05 | 0.161 | -0.20 | -0.37 – -0.03 | **0.025** | -0.01 | -0.18 – 0.17 | 0.954 | -0.03 | -0.20 – 0.15 | 0.763 |
| Female | -0.08 | -0.39 – 0.22 | 0.584 | 0.08 | -0.23 – 0.39 | 0.608 | 0.11 | -0.19 – 0.41 | 0.474 | 0.23 | -0.07 – 0.53 | 0.129 | -0.43 | -0.72 – -0.14 | **0.003** | 0.33 | 0.03 – 0.63 | **0.029** |
| Gender (Other) | 1.17 | -0.32 – 2.65 | 0.122 | -0.75 | -2.21 – 0.72 | 0.314 | -1.10 | -2.55 – 0.34 | 0.134 | 0.30 | -1.12 – 1.72 | 0.676 | -0.34 | -1.74 – 1.06 | 0.635 | 0.12 | -1.32 – 1.56 | 0.874 |
| Education | -0.00 | -0.18 – 0.17 | 0.975 | 0.15 | -0.02 – 0.32 | 0.087 | 0.02 | -0.15 – 0.19 | 0.805 | -0.01 | -0.18 – 0.15 | 0.876 | -0.20 | -0.36 – -0.03 | **0.019** | 0.10 | -0.07 – 0.27 | 0.244 |
| Black | 0.02 | -0.44 – 0.48 | 0.928 | -0.32 | -0.76 – 0.12 | 0.156 | 0.38 | -0.06 – 0.82 | 0.088 | 0.01 | -0.42 – 0.44 | 0.973 | -0.20 | -0.64 – 0.24 | 0.368 | -0.27 | -0.71 – 0.16 | 0.217 |
| Native American | -0.21 | -1.73 – 1.30 | 0.781 | -0.90 | -2.39 – 0.59 | 0.234 | 0.24 | -1.24 – 1.72 | 0.753 | 1.07 | -0.38 – 2.52 | 0.148 | -0.24 | -1.68 – 1.20 | 0.747 | 0.26 | -1.21 – 1.74 | 0.727 |
| Asian | -0.37 | -0.95 – 0.21 | 0.211 | 0.11 | -0.46 – 0.68 | 0.698 | 0.25 | -0.32 – 0.81 | 0.391 | -0.32 | -0.87 – 0.23 | 0.256 | 0.19 | -0.37 – 0.75 | 0.503 | -0.16 | -0.72 – 0.40 | 0.568 |
| Pacific Islander | 0.08 | -1.38 – 1.55 | 0.911 | 0.01 | -1.43 – 1.44 | 0.993 | 0.32 | -1.10 – 1.74 | 0.655 | -0.13 | -1.52 – 1.26 | 0.854 | -0.08 | -1.46 – 1.31 | 0.913 | -0.20 | -1.61 – 1.21 | 0.779 |
| Multi-racial | -0.53 | -1.16 – 0.10 | 0.096 | -0.13 | -0.74 – 0.48 | 0.679 | 0.52 | -0.08 – 1.13 | 0.091 | -0.13 | -0.72 – 0.47 | 0.673 | 0.34 | -0.25 – 0.93 | 0.259 | -0.15 | -0.75 – 0.45 | 0.625 |

**Figure S4**. Distributions of outcome variables in Study 2

| Openness  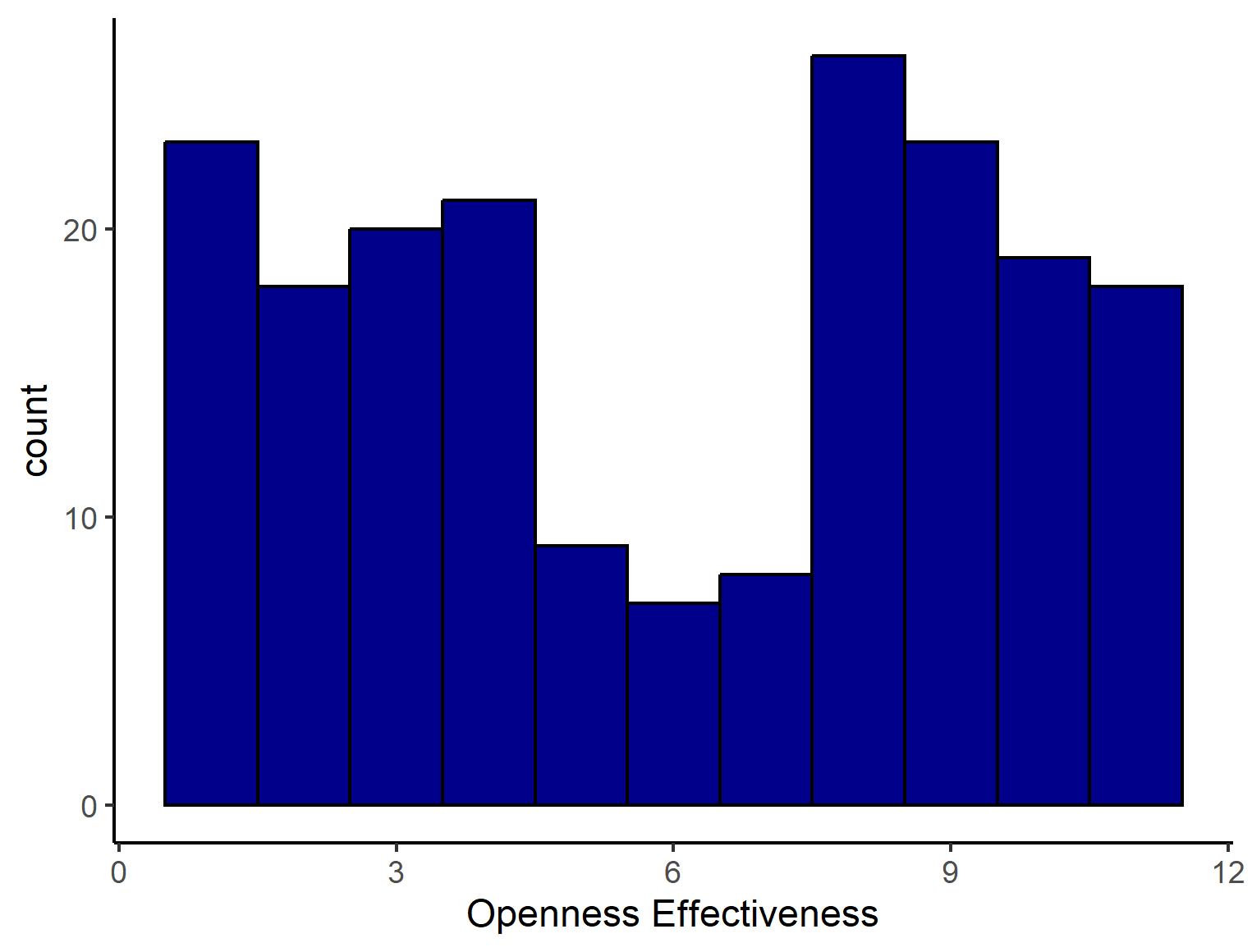 | Conscientiousness  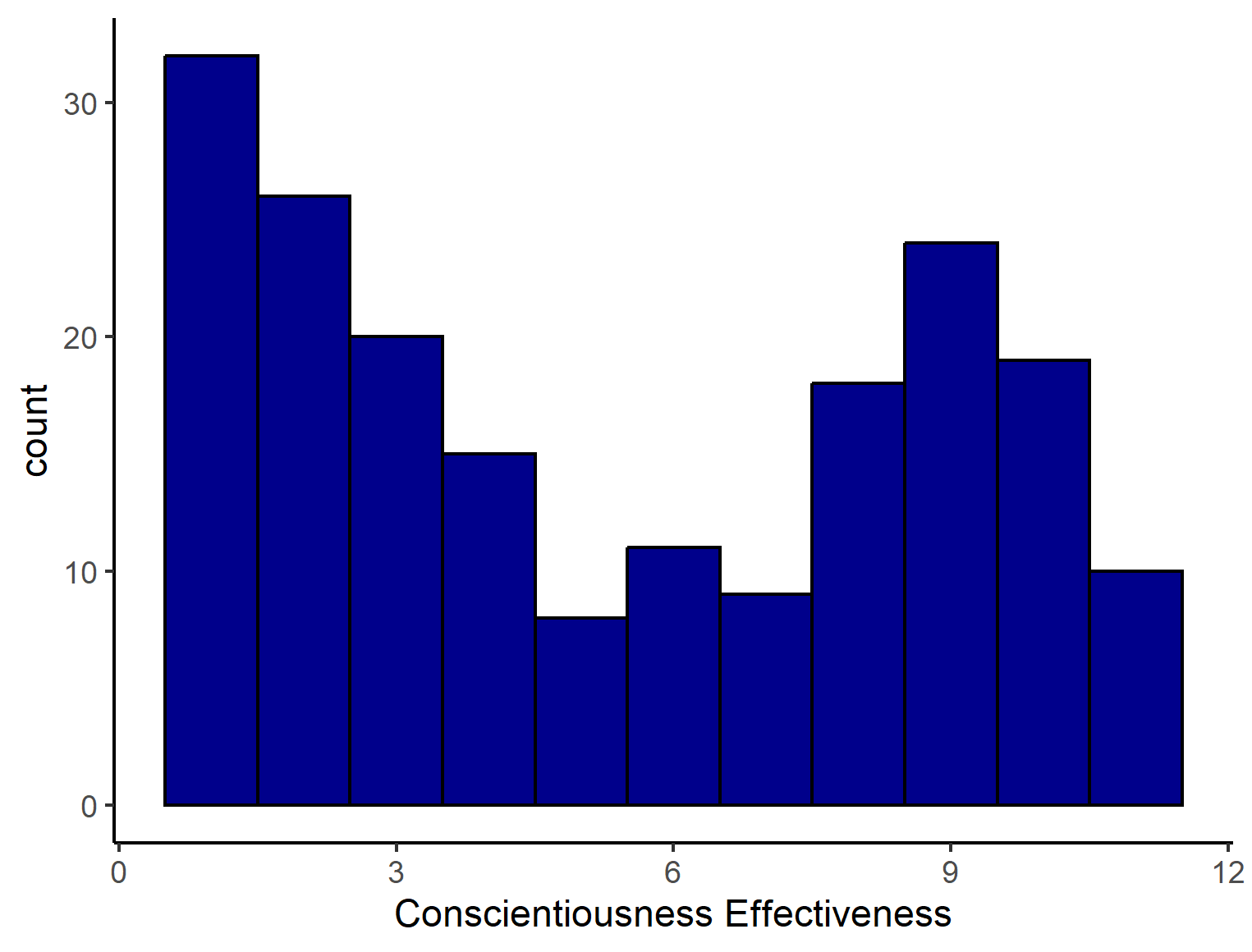 |
| --- | --- |
| Extraversion  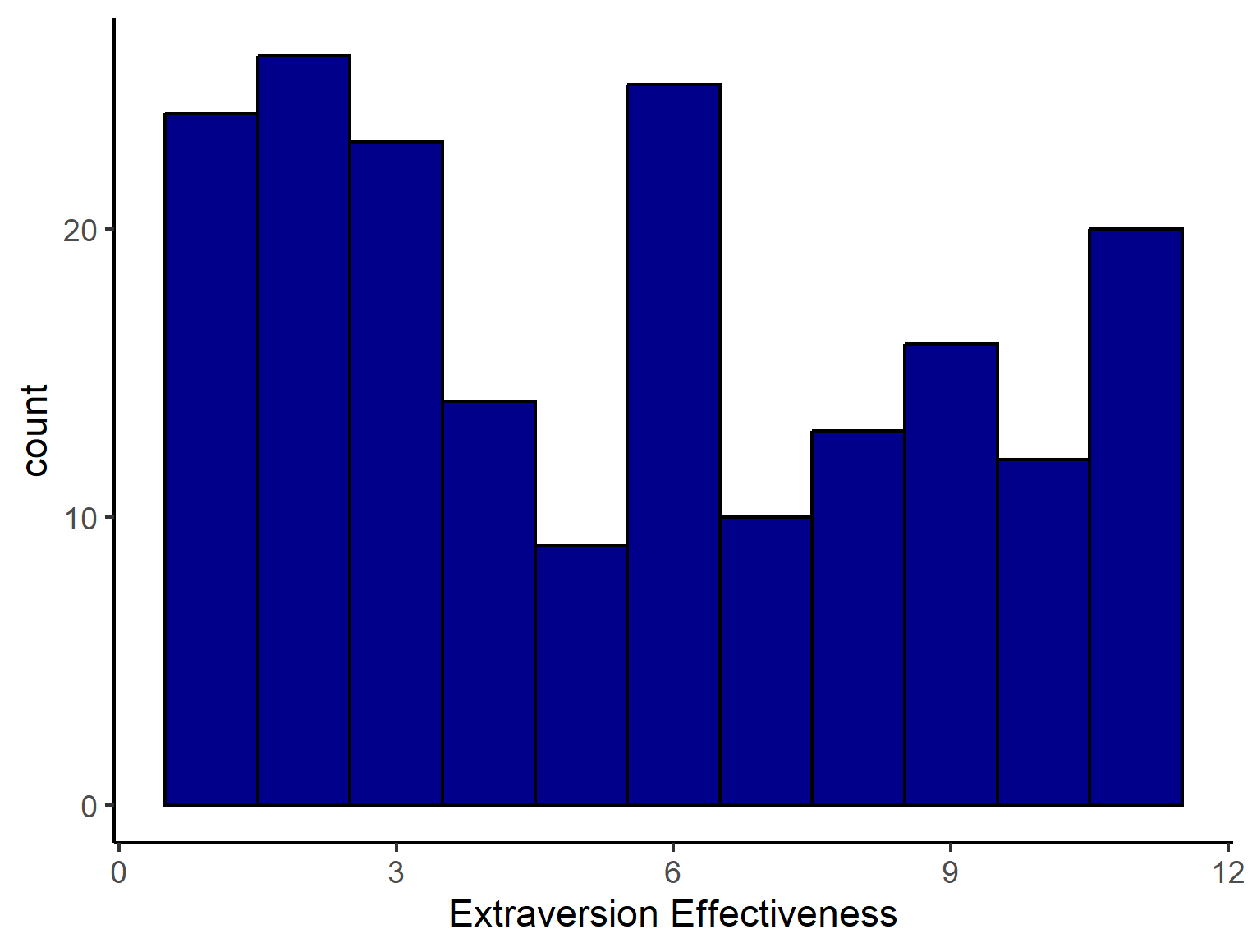 | Agreeableness  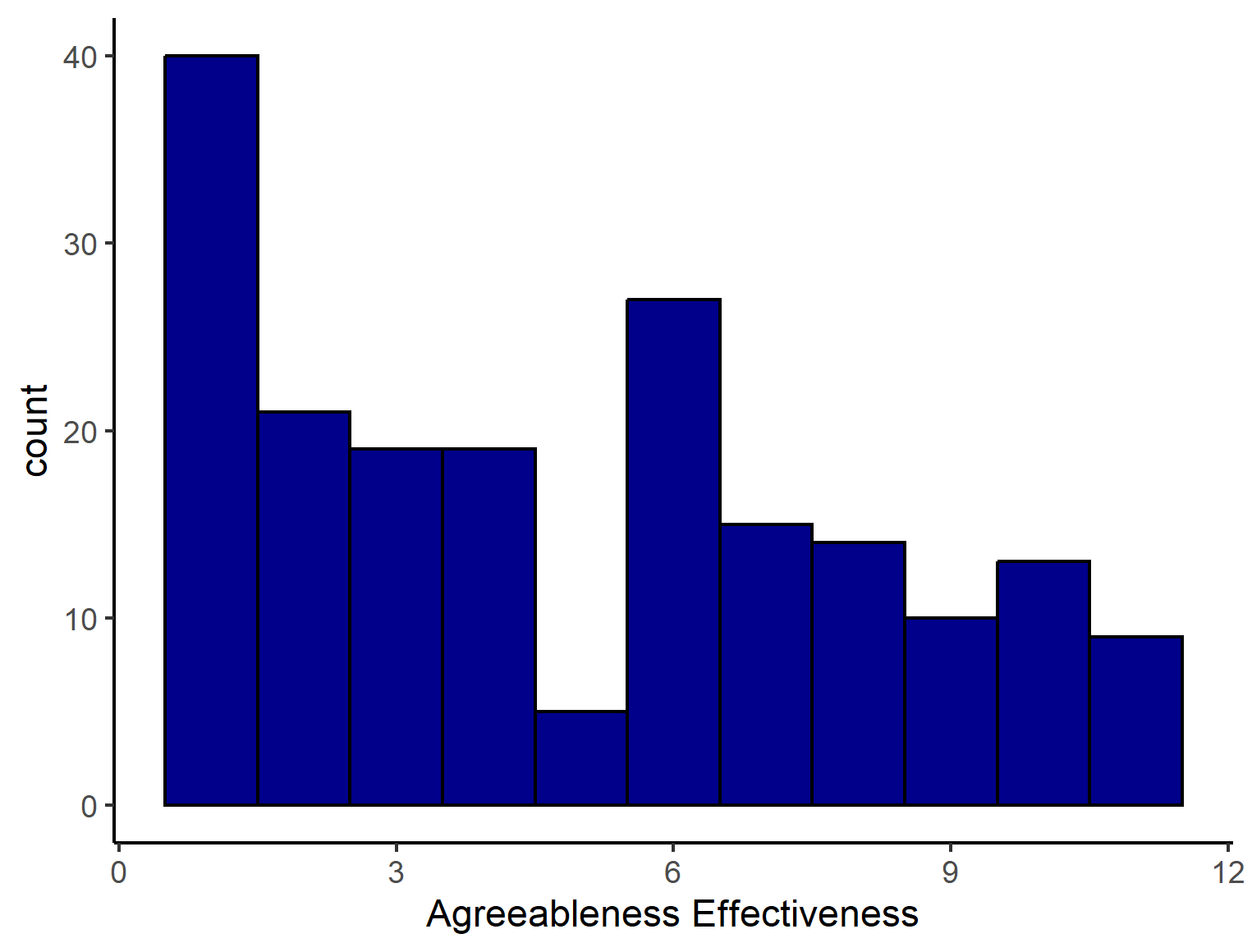 |
| Regulatory Focus  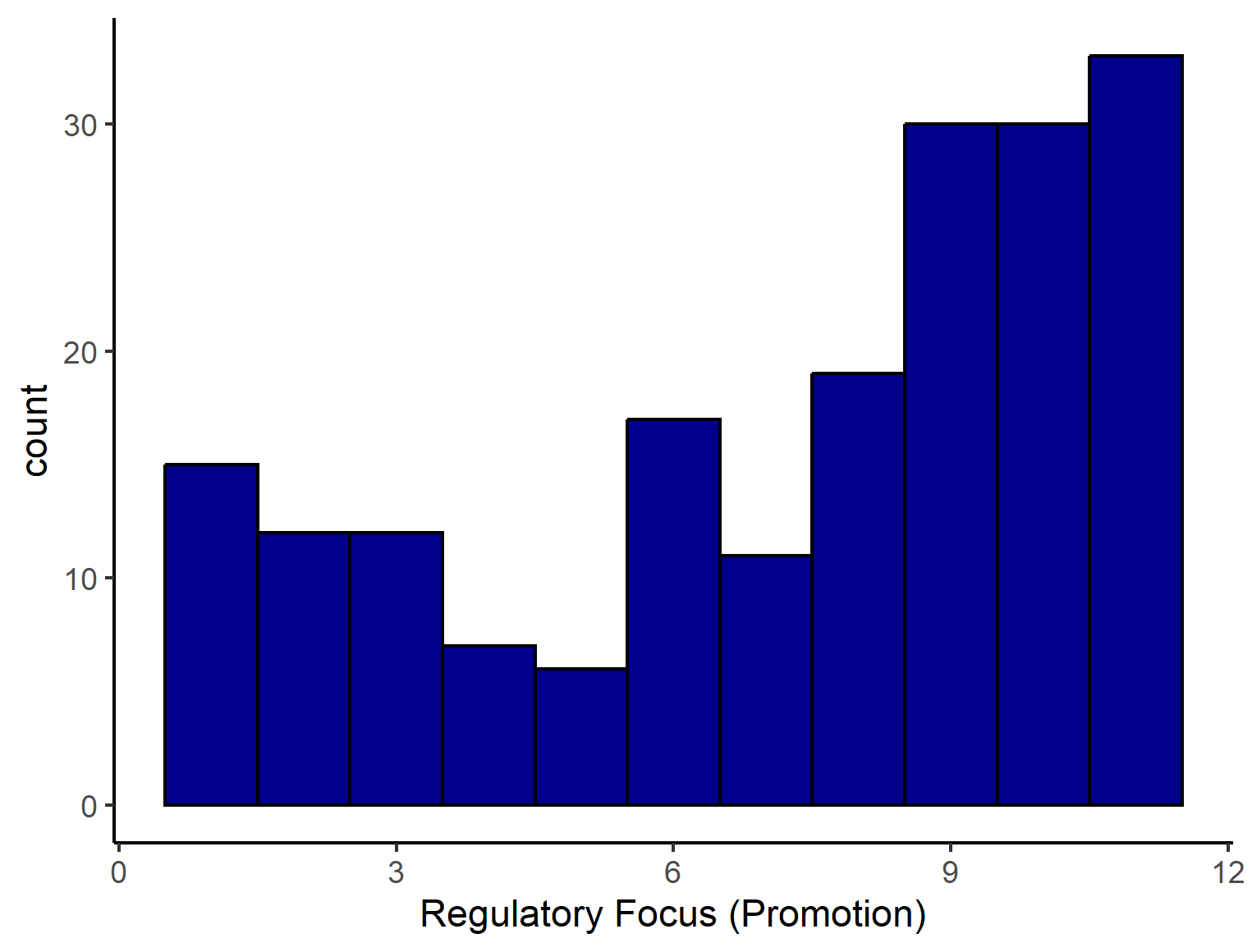 | Political Ideology  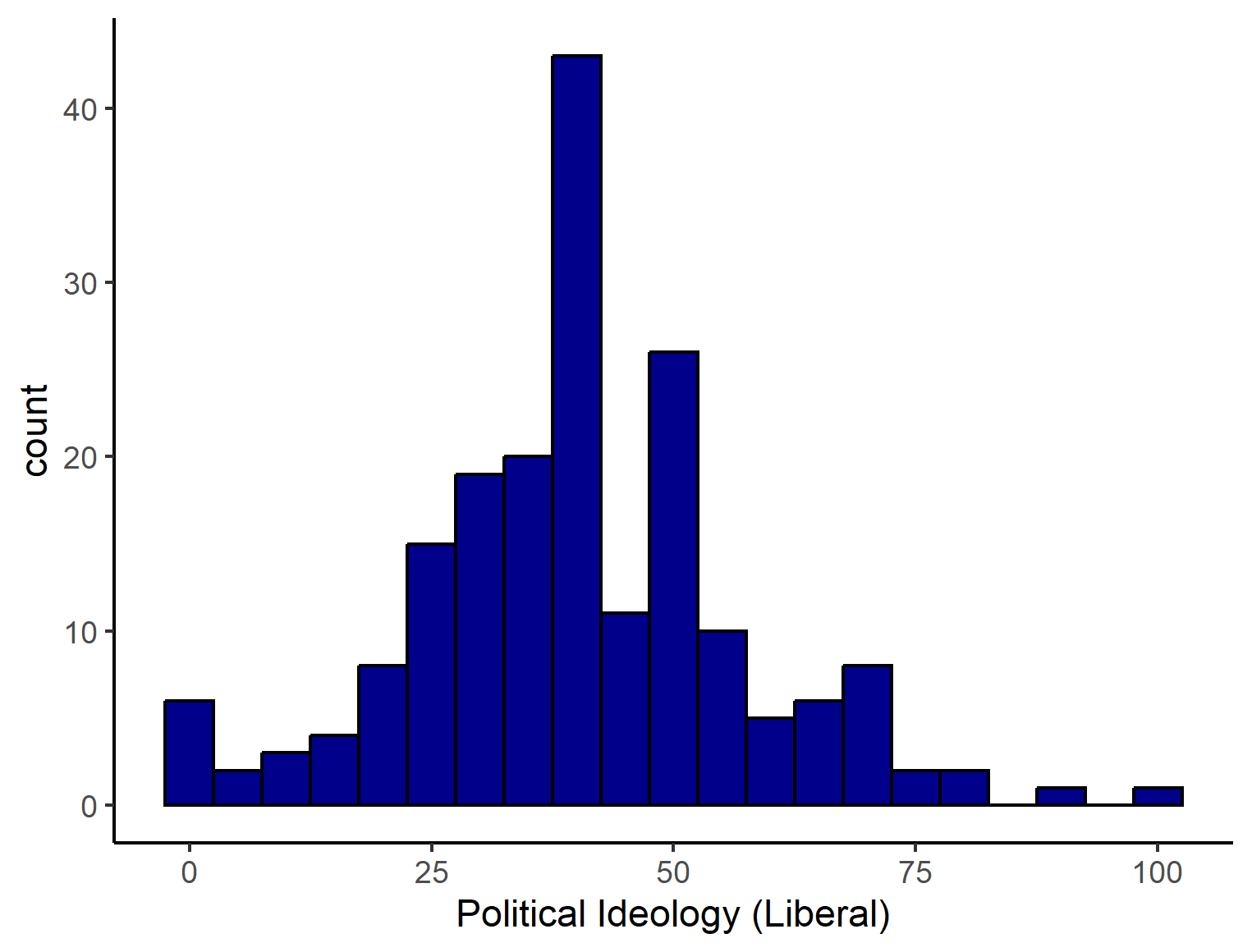 |
| Purity  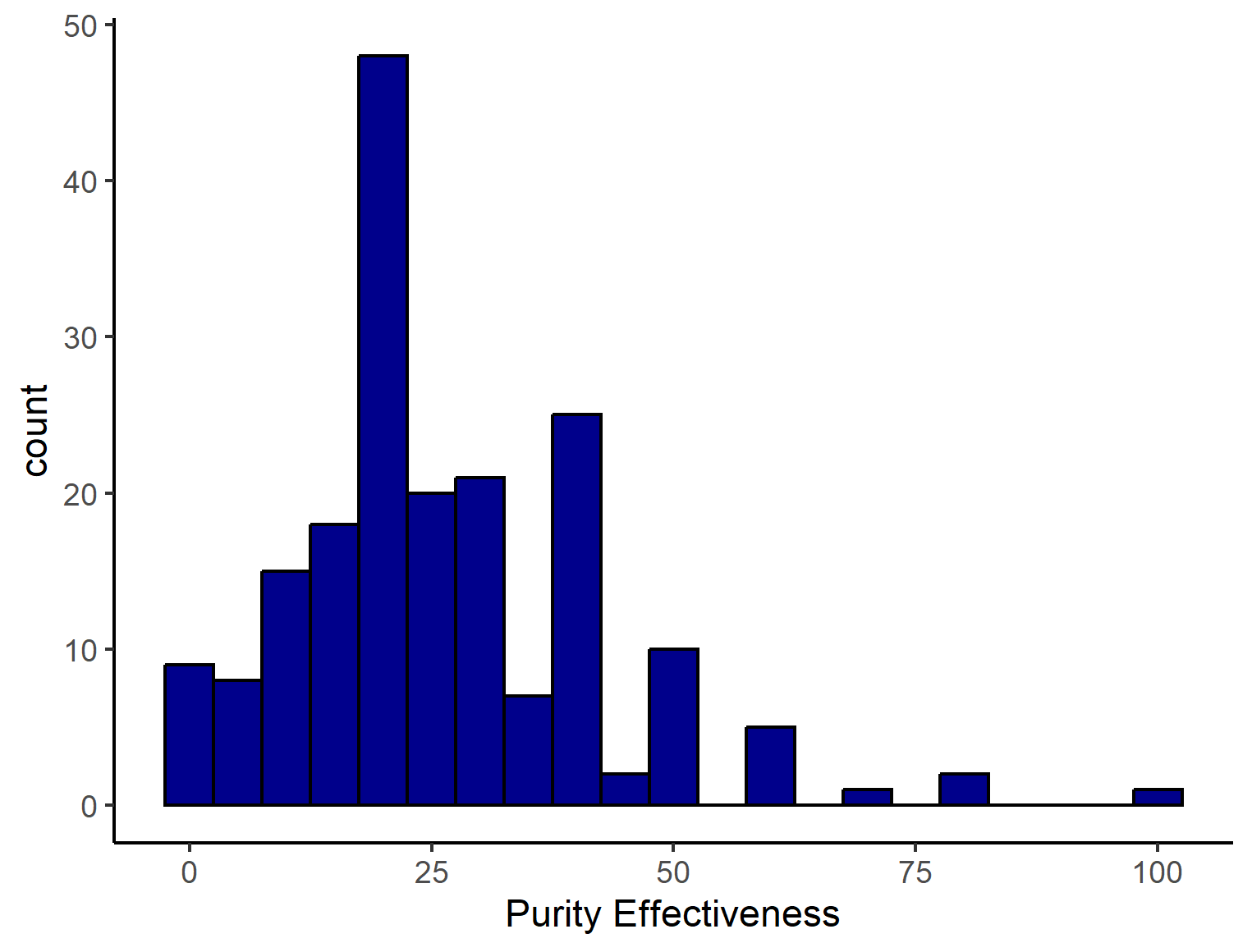 | Care  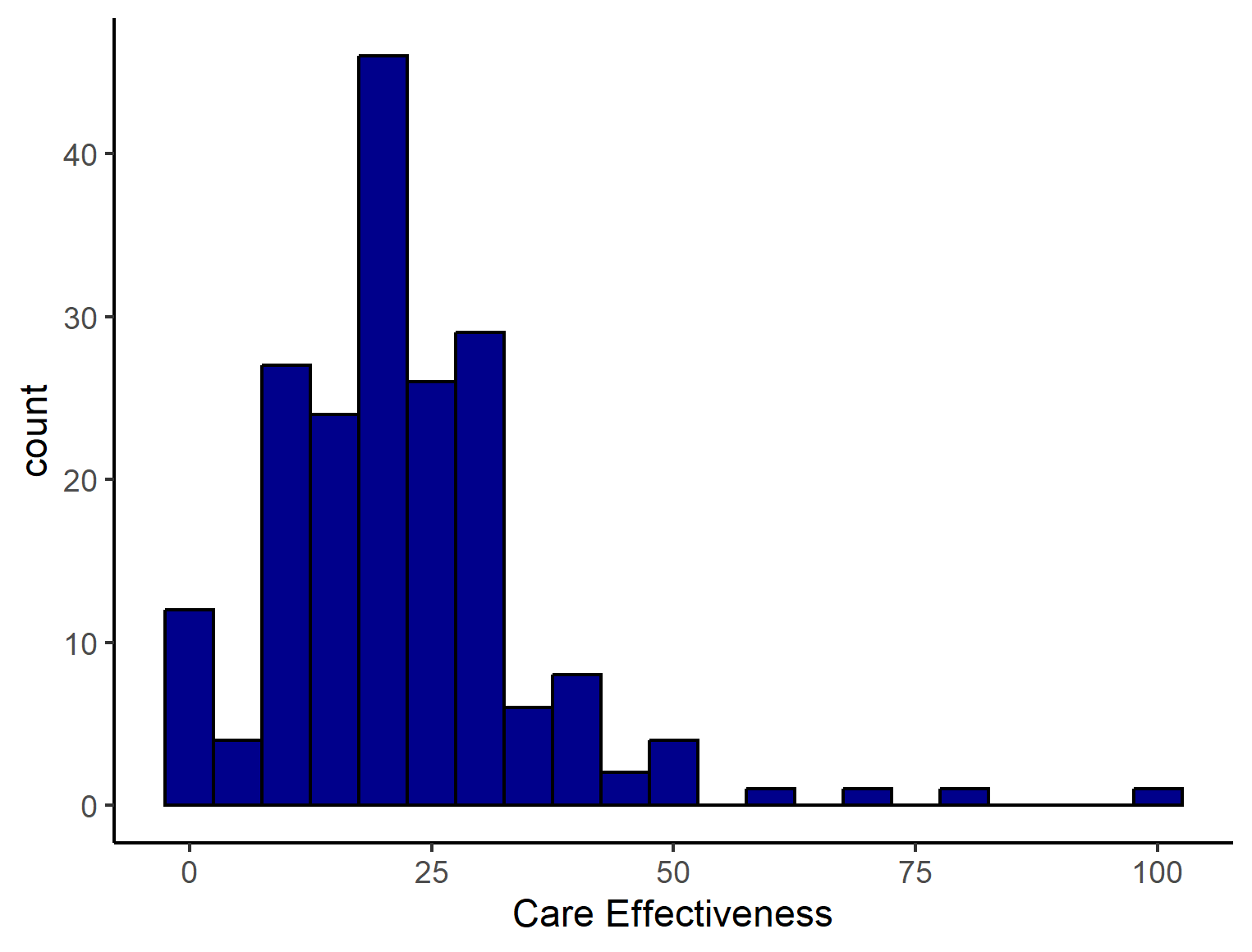 |
| Loyalty  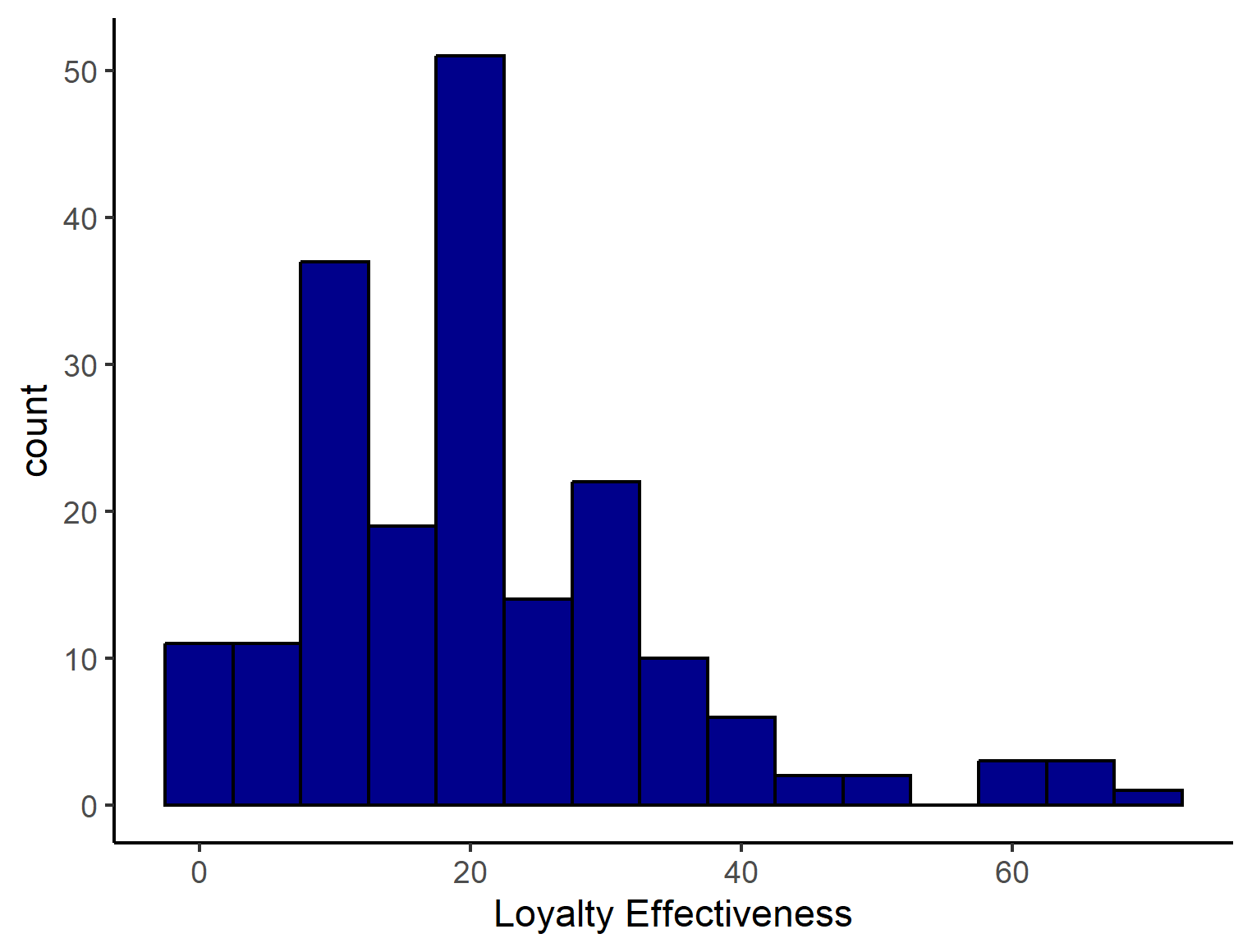 | Fairness  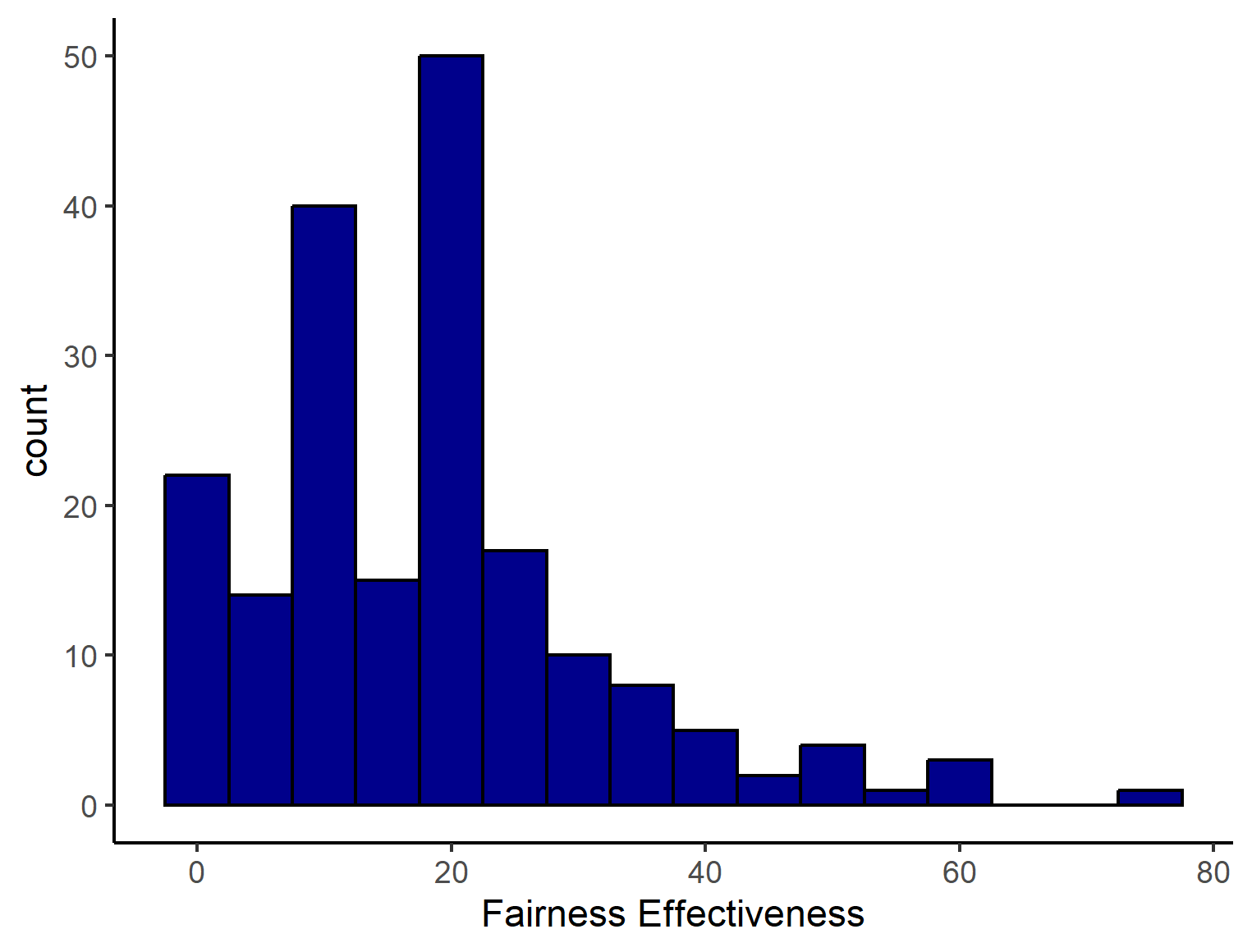 |
| Authority  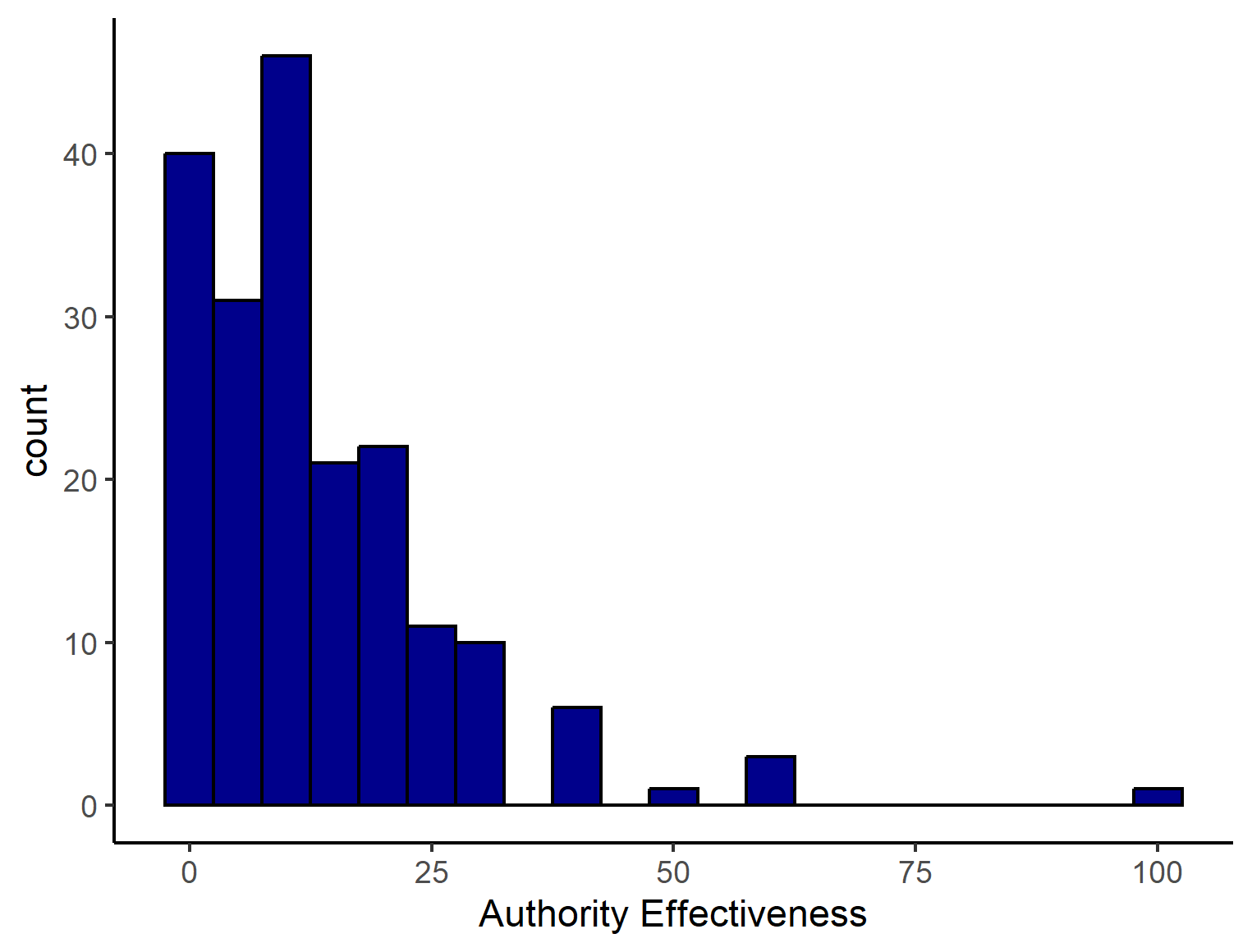 |  |

**Figure S5.** Marginal effects of Big Five personality traits on effectiveness ratings

***
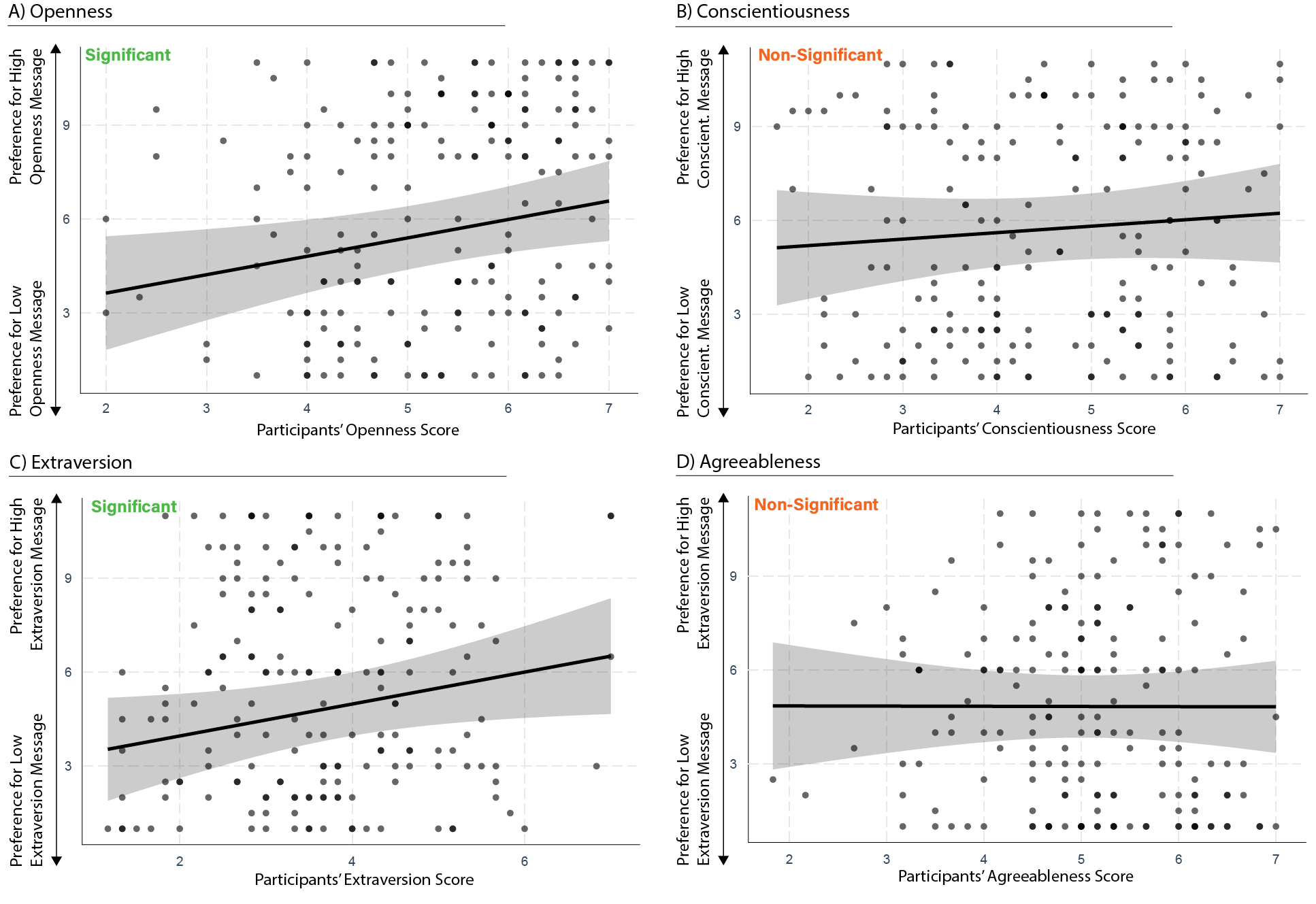
***

**Figure S6.** Marginal effects of Regulatory focus on effectiveness ratings

**
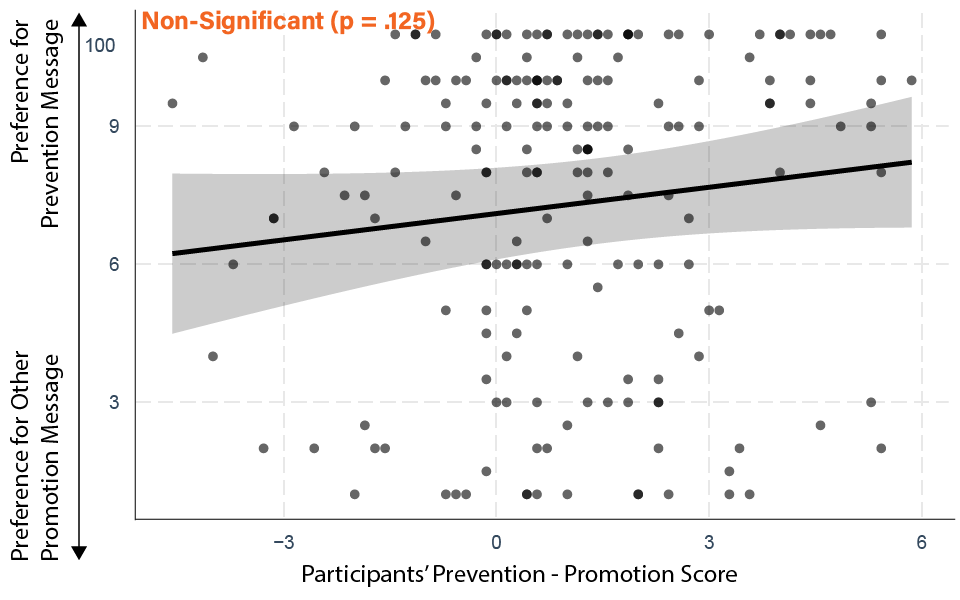
**

**Study 3a**

**Sample demographics**

| Age | 35.7 (13.4SD) |
| --- | --- |
| Gender | 50% Female |
| Ethnicity | 78% White  7% Black or African American  1% American Indian, Native American or Alaska Native  9% Asian  0% Hawaiian Native or Other Pacific Islander  1% Other  4% Multiracial |
| Education | 1% Some high school or less  10% High school diploma or GED  24% Some college but no degree  14% Associates or technical degree  40% Bachelor’s degree  11% Graduate or professional degree (MA, MS, MBA, PHD, JD, MD etc.) |
| Income | 20% Less than $10,000  6% $10,000 to $14,999  11% $15,000 to $24,999  10% $25,000 to $34,999  15% $35,000 to $49,999  14% $50,000 to $74,999  10% $75,000 to $99,999  8% $100,000 to $149,999  3% $150,000 to $199,999  2% $200,000 or more |
| Employment | 43% Employed (full-time)  12% Employed (part-time)  10% Self-employed  13% Unemployed  13% Student  7% Retired  2% Other |

**Table S23.** *Means, standard deviations, and zero-order correlations with 95% CI of variables in Study 3a*

| Variable | *M* | *SD* | 1 | 2 | 3 | 4 | 5 | 6 | 7 | 8 | 9 | 10 | 11 | 12 |
| --- | --- | --- | --- | --- | --- | --- | --- | --- | --- | --- | --- | --- | --- | --- |
|  |  |  |  |  |  |  |  |  |  |  |  |  |  |  |
| 1. OP (E) | 0.00 | 1.24 |  |  |  |  |  |  |  |  |  |  |  |  |
|  |  |  |  |  |  |  |  |  |  |  |  |  |  |  |
| 2. CO (E) | 0.00 | 1.09 | -.33** |  |  |  |  |  |  |  |  |  |  |  |
|  |  |  | [-.45, -.20] |  |  |  |  |  |  |  |  |  |  |  |
|  |  |  |  |  |  |  |  |  |  |  |  |  |  |  |
| 3. EX (E) | 0.00 | 1.19 | -.15* | -.17* |  |  |  |  |  |  |  |  |  |  |
|  |  |  | [-.29, -.01] | [-.31, -.03] |  |  |  |  |  |  |  |  |  |  |
|  |  |  |  |  |  |  |  |  |  |  |  |  |  |  |
| 4. AG (E) | 0.00 | 1.00 | -.12 | -.50** | -.25** |  |  |  |  |  |  |  |  |  |
|  |  |  | [-.26, .02] | [-.60, -.39] | [-.38, -.11] |  |  |  |  |  |  |  |  |  |
|  |  |  |  |  |  |  |  |  |  |  |  |  |  |  |
| 5. OP (WTP) | -0.00 | 128.81 | .65** | -.30** | -.09 | -.06 |  |  |  |  |  |  |  |  |
|  |  |  | [.56, .73] | [-.42, -.16] | [-.23, .05] | [-.20, .08] |  |  |  |  |  |  |  |  |
|  |  |  |  |  |  |  |  |  |  |  |  |  |  |  |
| 6. CO (WTP) | -0.00 | 101.91 | -.23** | .62** | -.04 | -.38** | -.41** |  |  |  |  |  |  |  |
|  |  |  | [-.36, -.09] | [.52, .70] | [-.18, .10] | [-.50, -.25] | [-.52, -.29] |  |  |  |  |  |  |  |
|  |  |  |  |  |  |  |  |  |  |  |  |  |  |  |
| 7. EX (WTP) | -0.00 | 143.36 | -.17* | -.16* | .57** | -.13 | -.27** | -.09 |  |  |  |  |  |  |
|  |  |  | [-.31, -.03] | [-.29, -.02] | [.46, .66] | [-.26, .01] | [-.39, -.13] | [-.23, .05] |  |  |  |  |  |  |
|  |  |  |  |  |  |  |  |  |  |  |  |  |  |  |
| 8. AG (WTP) | -0.00 | 101.77 | -.12 | -.25** | -.23** | .58** | -.16* | -.59** | -.34** |  |  |  |  |  |
|  |  |  | [-.26, .02] | [-.38, -.11] | [-.36, -.09] | [.47, .66] | [-.29, -.01] | [-.68, -.49] | [-.46, -.21] |  |  |  |  |  |
|  |  |  |  |  |  |  |  |  |  |  |  |  |  |  |
| 9. OP (P) | 5.25 | 1.04 | .22** | -.15* | -.11 | .02 | .11 | -.09 | .01 | -.03 |  |  |  |  |
|  |  |  | [.08, .35] | [-.28, -.01] | [-.24, .04] | [-.12, .16] | [-.03, .25] | [-.23, .05] | [-.13, .16] | [-.17, .11] |  |  |  |  |
|  |  |  |  |  |  |  |  |  |  |  |  |  |  |  |
| 10. CO (P) | 4.85 | 1.29 | .09 | .12 | -.03 | -.04 | .06 | .02 | -.03 | .02 | .09 |  |  |  |
|  |  |  | [-.05, .23] | [-.02, .26] | [-.17, .11] | [-.18, .10] | [-.08, .20] | [-.12, .16] | [-.18, .11] | [-.12, .16] | [-.05, .23] |  |  |  |
|  |  |  |  |  |  |  |  |  |  |  |  |  |  |  |
| 11. EX (P) | 3.72 | 1.15 | .05 | -.06 | .23** | .04 | .02 | -.06 | .20** | .00 | .15* | .46** |  |  |
|  |  |  | [-.09, .19] | [-.20, .09] | [.09, .36] | [-.11, .18] | [-.12, .16] | [-.20, .08] | [.06, .33] | [-.14, .15] | [.01, .28] | [.34, .56] |  |  |
|  |  |  |  |  |  |  |  |  |  |  |  |  |  |  |
| 12. AG (P) | 4.99 | 1.06 | .17* | .05 | -.04 | -.02 | .05 | .07 | -.07 | -.03 | .33** | .43** | .13 |  |
|  |  |  | [.03, .30] | [-.09, .19] | [-.18, .11] | [-.16, .13] | [-.09, .19] | [-.07, .21] | [-.21, .07] | [-.17, .11] | [.19, .45] | [.30, .54] | [-.01, .26] |  |
|  |  |  |  |  |  |  |  |  |  |  |  |  |  |  |
| 13. NE (P) | 3.71 | 1.29 | -.03 | .05 | .04 | -.10 | .00 | .03 | .03 | -.12 | -.14 | -.53** | -.48** | -.31** |
|  |  |  | [-.18, .11] | [-.09, .19] | [-.10, .18] | [-.24, .04] | [-.14, .15] | [-.11, .17] | [-.11, .17] | [-.26, .02] | [-.27, .01] | [-.63, -.42] | [-.58, -.36] | [-.43, -.18] |
|  |  |  |  |  |  |  |  |  |  |  |  |  |  |  |

*Note.* *M* and *SD* are used to represent mean and standard deviation, respectively. Values in square brackets indicate the 95% confidence interval for each correlation. (E) = effectiveness ratings, (WTP) = willingsness-to-pay and (P) = participants’ personality scores. * indicates *p* < .05. ** indicates *p* < .01.

**Table S24** Full model output of linear regression analyses for Effectiveness outcome in Study 3a

|  | Openness | | | Extraversion | | |
| --- | --- | --- | --- | --- | --- | --- |
| *Predictors* | *β* | *CI_95_* | *p* | *β* | *CI_95_* | *p* |
| (Intercept) | -.16 | -.46 – .14 | **.001** | -.07 | -.36 – .22 | .106 |
| Openness | .24 | .07 – .41 | **.005** | -.14 | -.30 – .02 | .095 |
| Conscientious. | .06 | -.14 – .26 | .532 | -.04 | -.23 – .15 | .649 |
| Extraversion | -.00 | -.18 – .18 | .985 | .35 | .18 – .52 | **<.001** |
| Agreeableness | .01 | -.17 – .20 | .877 | .02 | -.16 – .19 | .860 |
| Neuroticism | .09 | -.09 – .28 | .330 | .21 | .03 – .39 | **.023** |
| Age | .18 | -.03 – .38 | .086 | .13 | -.06 – .33 | .182 |
| Female | .24 | -.07 – .55 | .131 | -.01 | -.31 – .29 | .955 |
| Gender (Other) | .02 | -1.06 – 1.10 | .977 | .02 | -1.01 – 1.05 | .970 |
| Education | .03 | -.12 – .19 | .681 | -.08 | -.22 – .07 | .318 |
| Black | .05 | -.51 – .62 | .849 | .23 | -.31 – .77 | .393 |
| Native American | -1.41 | -2.93 – .12 | .070 | 1.18 | -.28 – 2.64 | .111 |
| Asian | -.33 | -.85 – .18 | .206 | -.30 | -.80 – .20 | .238 |
| Pacific Islander | .62 | -.82 – 2.07 | .395 | 1.38 | -.00 – 2.75 | .050 |
| Multi-racial | -.30 | -1.04 – .45 | .432 | .98 | .27 – 1.69 | **.007** |
| Part-time | .31 | -.17 – .80 | .198 | .19 | -.27 – .65 | .423 |
| Self-employed | .05 | -.47 – .57 | .851 | -.42 | -.92 – .07 | .093 |
| Unemployed | .31 | -.19 – .81 | .224 | -.03 | -.51 – .45 | .902 |
| Retired | .29 | -.23 – .80 | .270 | .49 | .00 – .98 | **.049** |
| Student | -.37 | -1.08 – .34 | .311 | -.34 | -1.01 – .34 | .331 |
| Employ. (Other) | -.29 | -1.40 – .82 | .601 | .00 | -1.06 – 1.06 | .997 |
| Average Effectiveness | .18 | .02 – .34 | **.024** | .01 | -.14 – .16 | .946 |
| Order OP ad | .08 | -.07 – .22 | .291 |  |  |  |
| Order CO ad |  |  |  |  |  |  |
| Order EX ad |  |  |  | .09 | -.05 – .23 | .188 |
| Order AG ad |  |  |  |  |  |  |
| Observations | 192 | | | 192 | | |
| R^2^ / R^2^ adjusted | .166 / .057 | | | .238 / .139 | | |

**Table S25.** Full model output of linear regression analyses for WTP outcome in Study 3a

|  | **Openness** | | | **Extraversion** | | |
| --- | --- | --- | --- | --- | --- | --- |
| *Predictors* | *β* | *CI_95_* | *p* | *β* | *CI_95_* | *p* |
| (Intercept) | -.01 | -.33 – .30 | **.034** | -.06 | -.36 – .24 | .325 |
| Openness | .15 | -.02 – .33 | .085 | -.04 | -.21 – .13 | .629 |
| Conscientious. | .09 | -.12 – .29 | .406 | -.03 | -.22 – .17 | .771 |
| Extraversion | .02 | -.17 – .20 | .847 | .26 | .09 – .44 | **.004** |
| Agreeableness | .00 | -.19 – .19 | .990 | -.05 | -.23 – .13 | .584 |
| Neuroticism | .12 | -.07 – .31 | .221 | .12 | -.06 – .31 | .196 |
| Age | .04 | -.17 – .25 | .710 | .09 | -.11 – .29 | .355 |
| Female | -.13 | -.45 – .19 | .439 | -.15 | -.46 – .16 | .329 |
| Gender (Other) | -.16 | -1.27 – .95 | .777 | -.35 | -1.42 – .71 | .516 |
| Education | .02 | -.13 – .18 | .758 | -.00 | -.16 – .15 | .960 |
| Black | .37 | -.21 – .95 | .206 | -.29 | -.84 – .27 | .312 |
| Native American | -1.56 | -3.12 – .01 | .051 | 1.35 | -.15 – 2.85 | .078 |
| Asian | -.44 | -.97 – .10 | .107 | -.34 | -.86 – .17 | .186 |
| Pacific Islander | -.04 | -1.52 – 1.44 | .954 | .62 | -.80 – 2.04 | .391 |
| Multi-racial | -.15 | -.91 – .61 | .690 | .60 | -.13 – 1.33 | .109 |
| Part-time | .11 | -.38 – .61 | .646 | .30 | -.18 – .77 | .217 |
| Self-employed | .24 | -.29 – .78 | .366 | .19 | -.32 – .70 | .461 |
| Unemployed | .39 | -.12 – .91 | .136 | .17 | -.32 – .67 | .494 |
| Retired | .13 | -.40 – .66 | .632 | .61 | .10 – 1.11 | **.019** |
| Student | .20 | -.53 – .93 | .593 | -.26 | -.96 – .44 | .464 |
| Employ. (Other) | -.34 | -1.48 – .80 | .559 | .76 | -.33 – 1.85 | .172 |
| Average WTP | .20 | .04 – .36 | **.016** | .03 | -.12 – .19 | .671 |
| Order OP ad | -.07 | -.22 – .08 | .389 |  |  |  |
| Order CO ad |  |  |  |  |  |  |
| Order EX ad |  |  |  | -.07 | -.22 – .07 | .308 |
| Order AG ad |  |  |  |  |  |  |
| Observations | 192 | | | 192 | | |
| R^2^ / R^2^ adjusted | .123 / .008 | | | .190 / .084 | | |

**Figure S7**. Distributions of residualized effectiveness and WTP outcome variables in Study 3a

| Openness (Effectiveness)  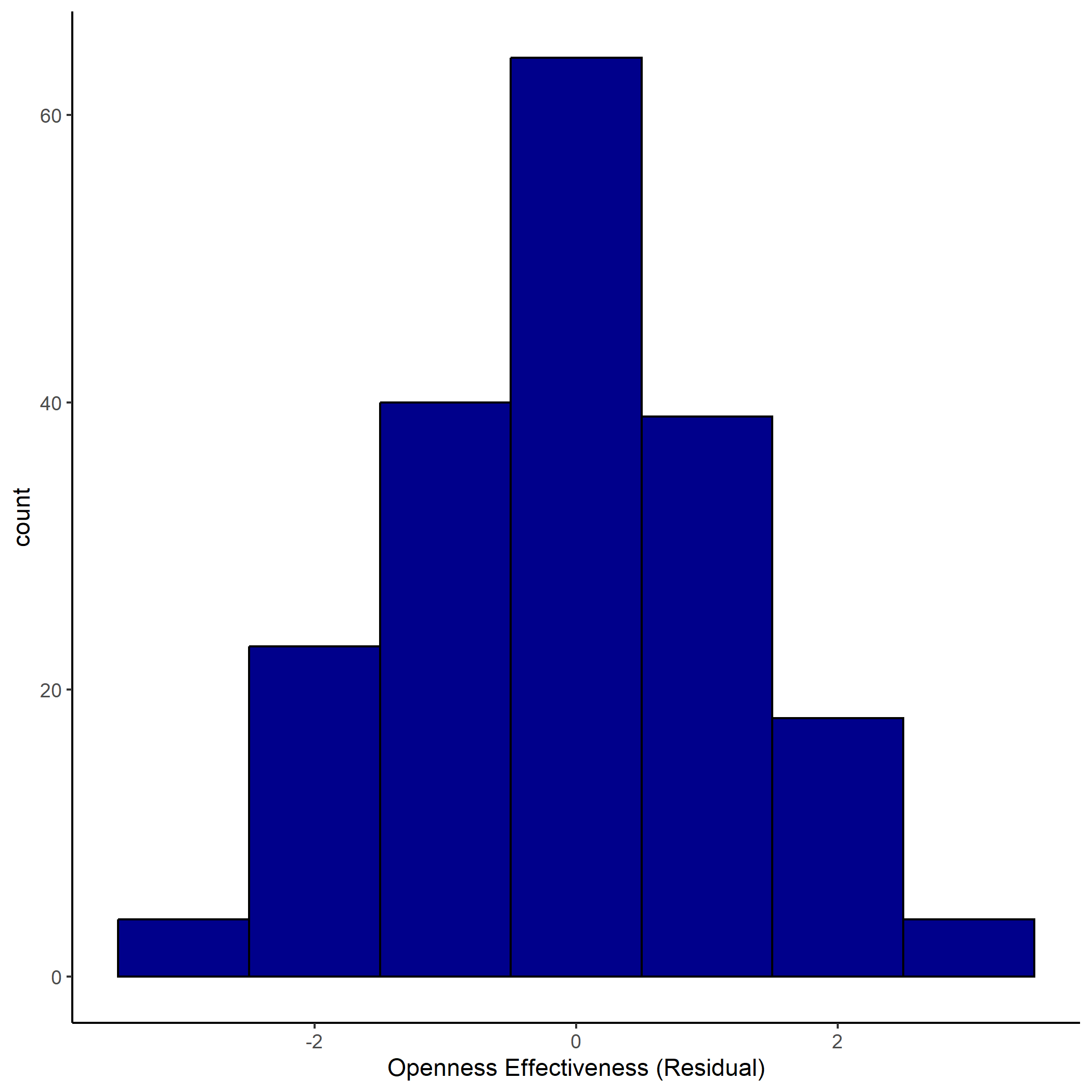 | Openness (WTP)  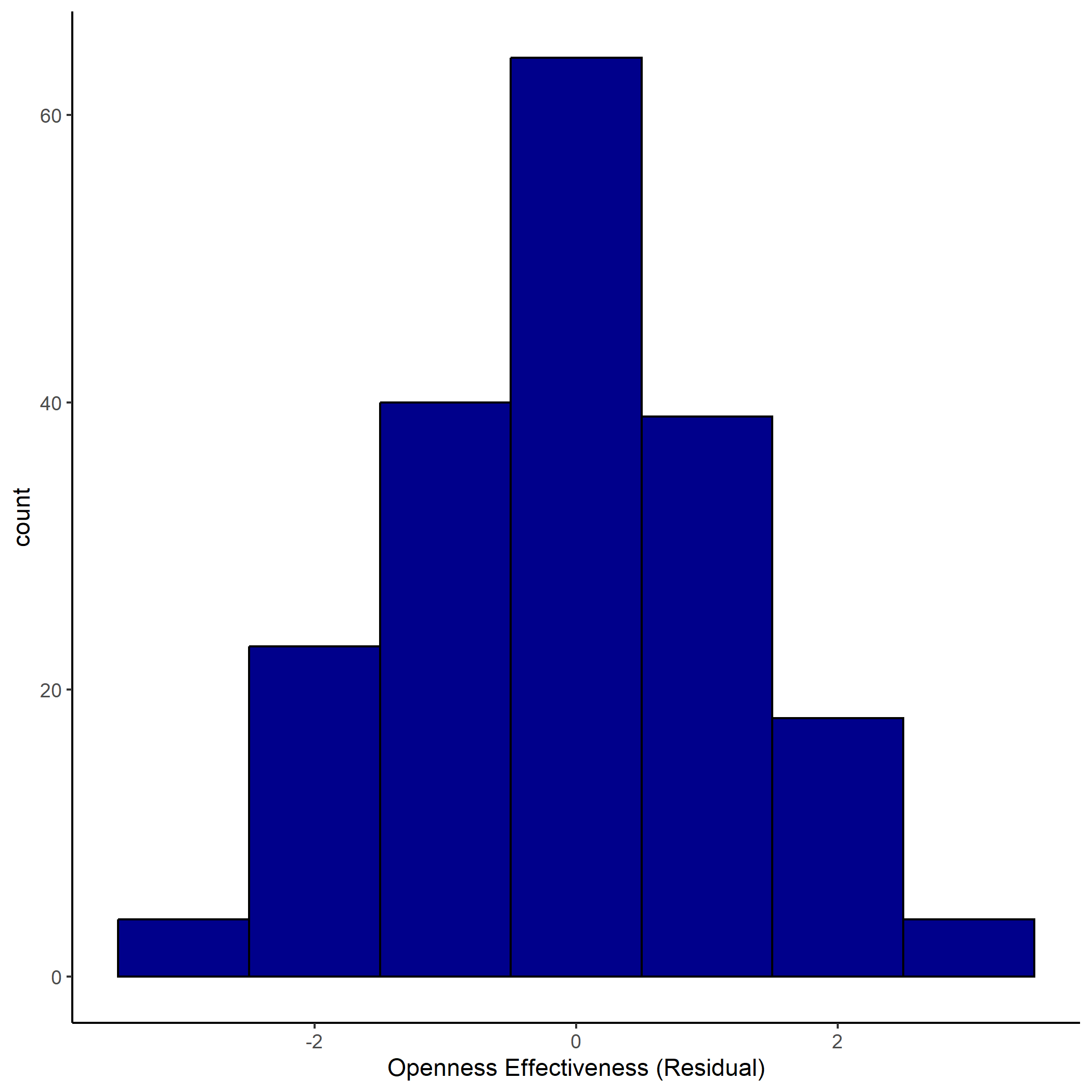 |
| --- | --- |
| Extraversion (Effectiveness)  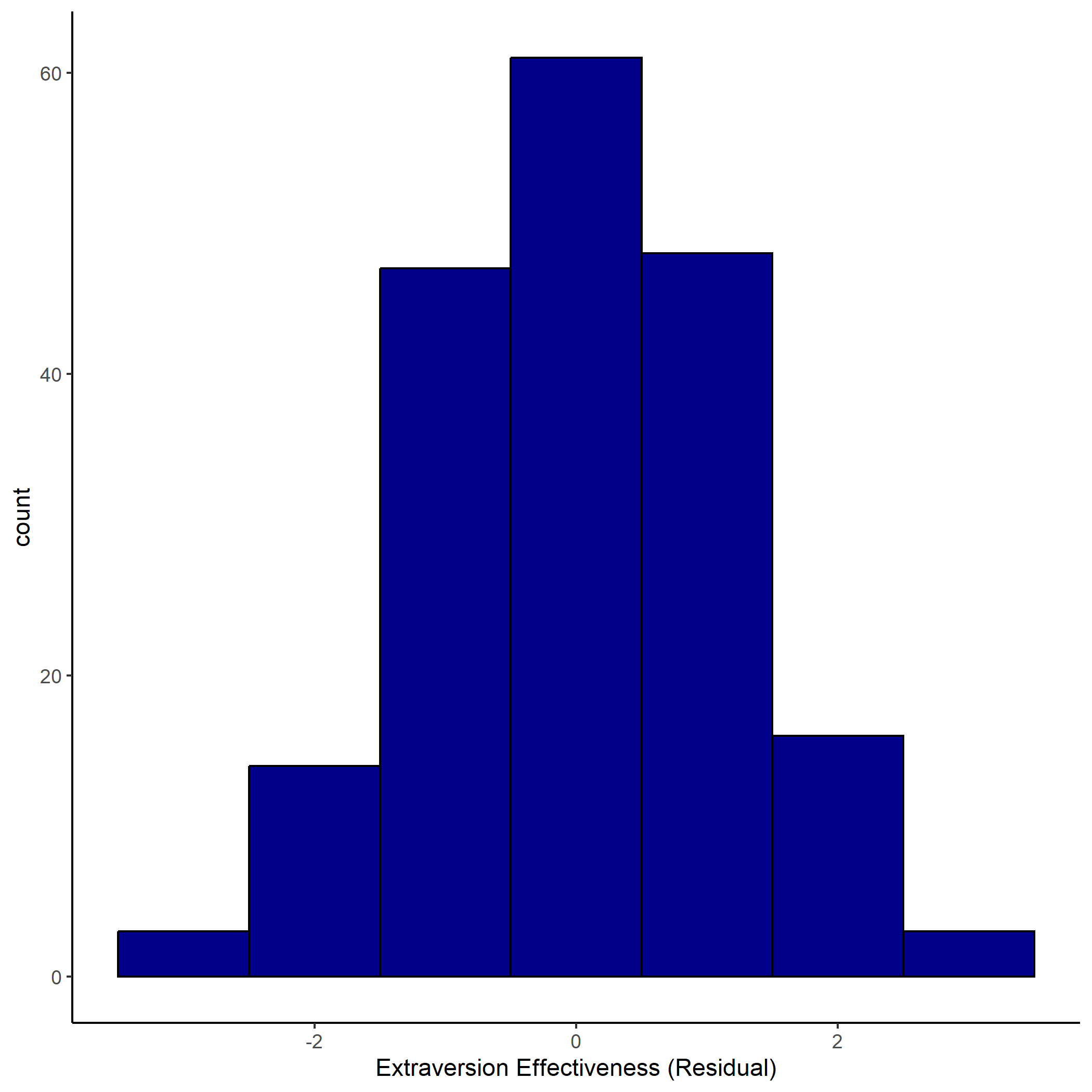 | Extraversion (WTP)  **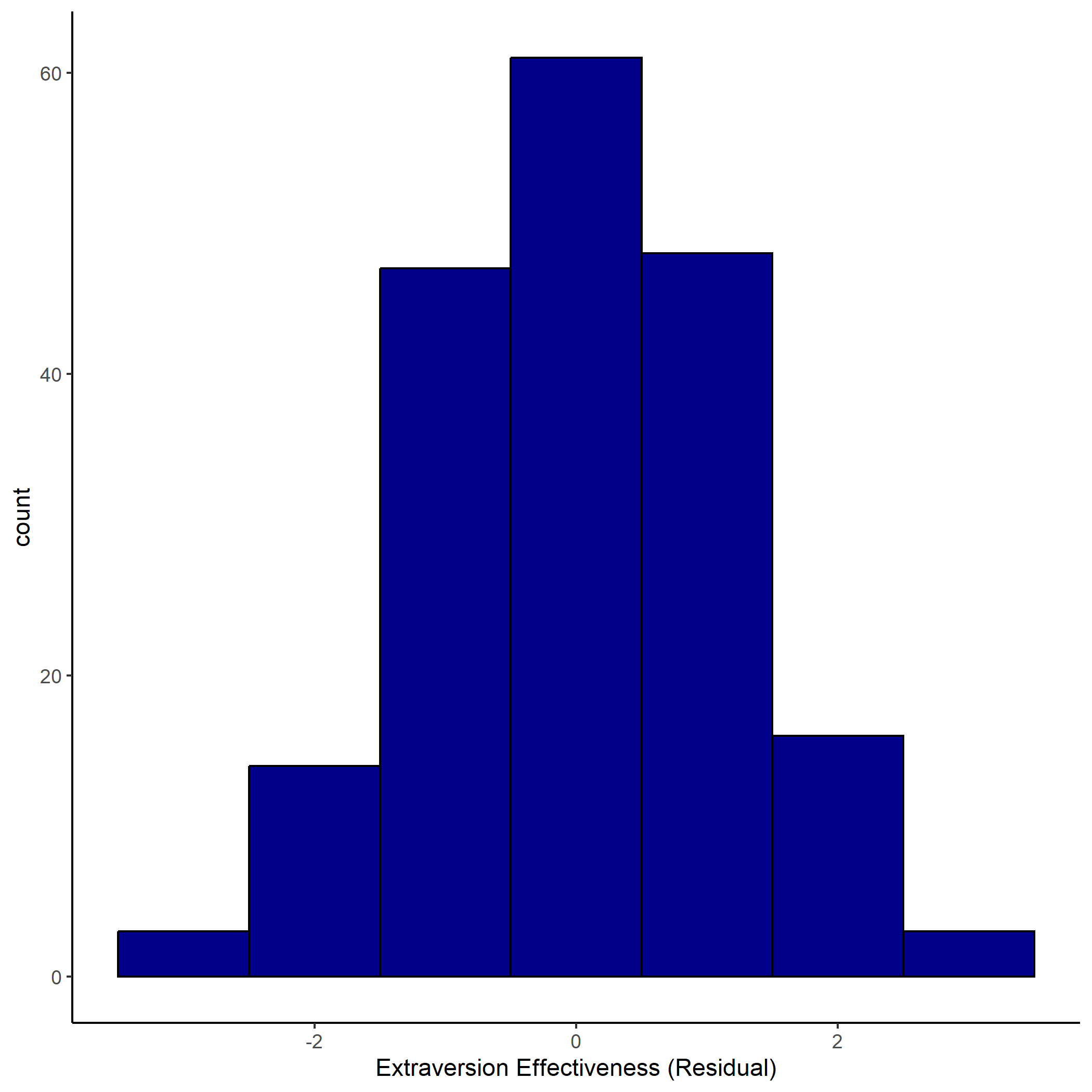** |

**Study 3b**

**Sample demographics**

| Age | 39.7 (14.59SD) |
| --- | --- |
| Gender | 48% Female |
| Ethnicity | 78% White  8% Black or African American  1% American Indian, Native American or Alaska Native  6% Asian  0% Hawaiian Native or Other Pacific Islander  4% Other  4% Multiracial |
| Education | 1% Some high school or less  16% High school diploma or GED  24% Some college but no degree  12% Associates or technical degree  34% Bachelor’s degree  13% Graduate or professional degree (MA, MS, MBA, PHD, JD, MD etc.) |
| Income | 15% Less than $10,000  6% $10,000 to $14,999  9% $15,000 to $24,999  15% $25,000 to $34,999  17% $35,000 to $49,999  18% $50,000 to $74,999  10% $75,000 to $99,999  9% $100,000 to $149,999  2% $150,000 to $199,999  0% $200,000 or more |
| Employment | 46% Employed (full-time)  12% Employed (part-time)  11% Self-employed  18% Unemployed  4% Student  7% Retired  4% Other |

**Table S26.** Means, standard deviations, and zero-order correlations with 95% CI of Moral Foundations in Study 3b

| Variable | *M* | *SD* | 1 | 2 | 3 | 4 |
| --- | --- | --- | --- | --- | --- | --- |
|  |  |  |  |  |  |  |
| 1. Liberal – Conservative (E) | 0.96 | 2.09 |  |  |  |  |
|  |  |  |  |  |  |  |
| 2. Liberal – Conservative (WTP) | 1.07 | 8.11 | .43** |  |  |  |
|  |  |  | [.31, .54] |  |  |  |
|  |  |  |  |  |  |  |
| 3. Political Ideology (P) | 4.93 | 1.75 | .14* | .25** |  |  |
|  |  |  | [.00, .28] | [.11, .38] |  |  |
|  |  |  |  |  |  |  |
| 4. Age | 39.73 | 14.59 | -.06 | .01 | -.17* |  |
|  |  |  | [-.20, .08] | [-.13, .15] | [-.30, -.03] |  |
|  |  |  |  |  |  |  |
| 5. Education | 4.01 | 1.35 | -.10 | -.08 | .14* | .14* |
|  |  |  | [-.24, .04] | [-.21, .07] | [.00, .28] | [.00, .28] |
|  |  |  |  |  |  |  |

*Note.* *M* and *SD* are used to represent mean and standard deviation, respectively. Values in square brackets indicate the 95% confidence interval for each correlation. (E) = effectiveness ratings, (WTP) = willingsness-to-pay and (P) = participants’ political ideology. * indicates *p* < .05. ** indicates *p* < .01.

**Table S27.** Full model output of linear regression analyses for Effectiveness and WTP outcome in Study 3b

|  | **Effectiveness** | | | **WTP** | | |
| --- | --- | --- | --- | --- | --- | --- |
| *Predictors* | *β* | *CI_95_* | *p* | *β* | *CI_95_* | *p* |
| (Intercept) | -0.09 | -0.35 – 0.17 | 0.944 | -0.40 | -0.66 – -0.15 | 0.290 |
| Political Ideology | 0.18 | 0.03 – 0.33 | **0.019** | 0.24 | 0.10 – 0.39 | **0.001** |
| Age | -0.02 | -0.20 – 0.15 | 0.794 | 0.08 | -0.09 – 0.25 | 0.332 |
| Female | -0.13 | -0.44 – 0.17 | 0.387 | 0.19 | -0.10 – 0.48 | 0.194 |
| Gender (Other) | 1.19 | -0.02 – 2.41 | 0.055 | 0.25 | -0.91 – 1.42 | 0.668 |
| Education | -0.06 | -0.22 – 0.10 | 0.445 | -0.04 | -0.19 – 0.11 | 0.580 |
| Black | 0.04 | -0.48 – 0.56 | 0.882 | 0.58 | 0.08 – 1.08 | **0.024** |
| Native American | -0.97 | -2.96 – 1.02 | 0.336 | -0.46 | -2.37 – 1.44 | 0.632 |
| Asian | 0.17 | -0.46 – 0.81 | 0.591 | 0.32 | -0.30 – 0.93 | 0.309 |
| Pacific Islander | 0.24 | -0.48 – 0.97 | 0.508 | 0.59 | -0.10 – 1.28 | 0.095 |
| Multi-racial | 0.50 | -0.31 – 1.31 | 0.228 | 0.51 | -0.26 – 1.29 | 0.194 |
| Part-time | 0.10 | -0.39 – 0.59 | 0.681 | 0.24 | -0.23 – 0.71 | 0.312 |
| Self-employed | -0.06 | -0.56 – 0.43 | 0.806 | 0.33 | -0.15 – 0.80 | 0.177 |
| Unemployed | 0.48 | 0.05 – 0.90 | **0.028** | 0.65 | 0.25 – 1.06 | **0.002** |
| Retired | 0.01 | -0.81 – 0.83 | 0.981 | 0.12 | -0.67 – 0.91 | 0.773 |
| Student | 0.45 | -0.21 – 1.11 | 0.180 | 0.25 | -0.39 – 0.88 | 0.443 |
| Employ. (Other) | -0.51 | -1.35 – 0.33 | 0.230 | 0.04 | -0.76 – 0.84 | 0.925 |
| Order Purity ad | -0.04 | -0.22 – 0.14 | 0.682 | -0.02 | -0.19 – 0.15 | 0.812 |
| Order Care ad | -0.01 | -0.19 – 0.18 | 0.935 | 0.07 | -0.11 – 0.25 | 0.462 |
| Order Loyalty ad | 0.01 | -0.17 – 0.20 | 0.883 | -0.15 | -0.33 – 0.03 | 0.100 |
| Order Fairness ad | -0.01 | -0.19 – 0.17 | 0.876 | -0.03 | -0.21 – 0.14 | 0.700 |
| Average effectiveness/WTP | 0.19 | 0.04 – 0.33 | **0.011** | 0.04 | -0.09 – 0.18 | 0.525 |
| Observations | 196 | | | 196 | | |
| R^2^ / R^2^ adjusted | 0.141 / 0.038 | | | 0.210 / 0.114 | | |

**Table S28.** Full model output of linear regression analyses for Effectiveness separately for each Moral Foundation in Study 3b

|  | **Authority** | | | **Care** | | | **Fairness** | | | **Loyalty** | | | **Purity** | | |
| --- | --- | --- | --- | --- | --- | --- | --- | --- | --- | --- | --- | --- | --- | --- | --- |
| *Predictors* | *β* | *CI_95_* | *p* | *β* | *CI_95_* | *p* | *β* | *CI_95_* | *p* | *β* | *CI_95_* | *p* | *β* | *CI_95_* | *p* |
| (Intercept) | 0.15 | -0.12 – 0.41 | 0.893 | 0.06 | -0.18 – 0.30 | 0.211 | -0.09 | -0.35 – 0.16 | **0.007** | -0.15 | -0.41 – 0.11 | 0.175 | 0.08 | -0.18 – 0.34 | **0.032** |
| Political Ideology | -0.08 | -0.24 – 0.08 | 0.325 | -0.11 | -0.25 – 0.03 | 0.124 | 0.34 | 0.19 – 0.49 | **<0.001** | -0.16 | -0.32 – -0.01 | **0.037** | 0.04 | -0.12 – 0.19 | 0.645 |
| Age | 0.06 | -0.12 – 0.24 | 0.522 | 0.00 | -0.16 – 0.16 | 0.997 | -0.02 | -0.19 – 0.15 | 0.793 | -0.04 | -0.21 – 0.13 | 0.668 | 0.00 | -0.17 – 0.18 | 0.969 |
| Female | -0.14 | -0.44 – 0.17 | 0.377 | -0.24 | -0.51 – 0.04 | 0.088 | -0.04 | -0.33 – 0.25 | 0.784 | 0.40 | 0.10 – 0.69 | **0.008** | -0.08 | -0.39 – 0.22 | 0.582 |
| Gender (Other) | -0.71 | -1.94 – 0.52 | 0.257 | 0.80 | -0.31 – 1.91 | 0.158 | 0.01 | -1.17 – 1.18 | 0.992 | -0.00 | -1.20 – 1.19 | 0.994 | -0.28 | -1.50 – 0.94 | 0.652 |
| Education | -0.03 | -0.19 – 0.12 | 0.690 | -0.11 | -0.26 – 0.03 | 0.112 | 0.03 | -0.12 – 0.18 | 0.665 | -0.10 | -0.25 – 0.05 | 0.207 | 0.21 | 0.05 – 0.37 | **0.008** |
| Black | 0.12 | -0.40 – 0.65 | 0.644 | -0.09 | -0.57 – 0.39 | 0.710 | 0.27 | -0.24 – 0.77 | 0.301 | -0.41 | -0.92 – 0.11 | 0.119 | 0.15 | -0.37 – 0.67 | 0.574 |
| Native American | 0.36 | -1.65 – 2.37 | 0.724 | -0.97 | -2.79 – 0.84 | 0.291 | -0.37 | -2.29 – 1.56 | 0.709 | 0.07 | -1.87 – 2.01 | 0.943 | 0.74 | -1.25 – 2.73 | 0.463 |
| Asian | -0.06 | -0.70 – 0.59 | 0.860 | 0.17 | -0.41 – 0.75 | 0.575 | 0.14 | -0.48 – 0.75 | 0.661 | -0.30 | -0.93 – 0.32 | 0.338 | 0.06 | -0.58 – 0.70 | 0.847 |
| Pacific Islander | -0.01 | -0.74 – 0.72 | 0.974 | 0.16 | -0.50 – 0.82 | 0.637 | 0.29 | -0.41 – 0.98 | 0.417 | -0.10 | -0.80 – 0.60 | 0.775 | -0.18 | -0.90 – 0.54 | 0.623 |
| Multi-racial | -0.38 | -1.19 – 0.44 | 0.361 | 0.19 | -0.55 – 0.93 | 0.610 | 0.62 | -0.16 – 1.41 | 0.118 | -0.31 | -1.10 – 0.49 | 0.448 | -0.03 | -0.84 – 0.78 | 0.942 |
| Part-time | -0.42 | -0.91 – 0.07 | 0.092 | -0.06 | -0.50 – 0.38 | 0.785 | 0.20 | -0.27 – 0.66 | 0.411 | 0.14 | -0.33 – 0.62 | 0.550 | -0.00 | -0.48 – 0.48 | 0.999 |
| Self-employed | -0.26 | -0.76 – 0.24 | 0.307 | -0.04 | -0.49 – 0.40 | 0.854 | 0.08 | -0.40 – 0.55 | 0.754 | 0.39 | -0.09 – 0.87 | 0.112 | -0.25 | -0.74 – 0.24 | 0.321 |
| Unemployed | -0.03 | -0.47 – 0.40 | 0.880 | 0.17 | -0.22 – 0.56 | 0.392 | -0.08 | -0.50 – 0.33 | 0.694 | -0.14 | -0.56 – 0.28 | 0.506 | 0.12 | -0.31 – 0.55 | 0.582 |
| Retired | -0.17 | -1.01 – 0.66 | 0.686 | -0.31 | -1.06 – 0.44 | 0.419 | 0.20 | -0.60 – 0.99 | 0.630 | -0.23 | -1.04 – 0.58 | 0.573 | 0.57 | -0.26 – 1.40 | 0.175 |
| Student | 0.16 | -0.50 – 0.83 | 0.629 | 0.46 | -0.14 – 1.06 | 0.131 | 0.29 | -0.34 – 0.93 | 0.365 | -0.10 | -0.75 – 0.54 | 0.752 | -0.60 | -1.25 – 0.06 | 0.073 |
| Employ. (Other) | 0.36 | -0.48 – 1.21 | 0.398 | -0.10 | -0.87 – 0.66 | 0.787 | 0.29 | -0.53 – 1.10 | 0.487 | 0.13 | -0.69 – 0.95 | 0.755 | -0.59 | -1.43 – 0.25 | 0.171 |
| Average effectiveness | 0.18 | 0.03 – 0.33 | **0.022** | 0.43 | 0.30 – 0.57 | **<0.001** | 0.06 | -0.09 – 0.20 | 0.430 | 0.17 | 0.02 – 0.32 | **0.023** | 0.08 | -0.07 – 0.23 | 0.310 |
| Order Authority | -0.06 | -0.21 – 0.09 | 0.422 |  |  |  |  |  |  |  |  |  |  |  |  |
| Order Care |  |  |  | 0.01 | -0.12 – 0.15 | 0.853 |  |  |  |  |  |  |  |  |  |
| Order Fairness |  |  |  |  |  |  | -0.02 | -0.16 – 0.12 | 0.819 |  |  |  |  |  |  |
| Order Loyalty |  |  |  |  |  |  |  |  |  | -0.18 | -0.32 – -0.04 | **0.012** |  |  |  |
| Order Purity |  |  |  |  |  |  |  |  |  |  |  |  | 0.06 | -0.09 – 0.20 | 0.449 |
| Observations | 196 | | | 196 | | | 196 | | | 196 | | | 196 | | |
| R^2^ / R^2^ adjusted | 0.096 / 0.004 | | | 0.266 / 0.191 | | | 0.172 / 0.088 | | | 0.151 / 0.064 | | | 0.114 / 0.024 | | |

**Table S29.** Full model output of linear regression analyses for WTP separately for each Moral Foundation in Study 3b

|  | **Authority** | | | **Care** | | | **Fairness** | | | **Loyalty** | | | **Purity** | | |
| --- | --- | --- | --- | --- | --- | --- | --- | --- | --- | --- | --- | --- | --- | --- | --- |
| *Predictors* | *β* | *CI_95_* | *p* | *β* | *CI_95_* | *p* | *β* | *CI_95_* | *p* | *β* | *CI_95_* | *p* | *β* | *CI_95_* | *p* |
| (Intercept) | 0.22 | -0.05 – 0.49 | 0.469 | -0.22 | -0.48 – 0.04 | 0.553 | -0.24 | -0.50 – 0.02 | **<0.001** | 0.01 | -0.25 – 0.27 | **0.034** | 0.19 | -0.07 – 0.46 | 0.514 |
| Political Ideology | -0.04 | -0.20 – 0.11 | 0.584 | 0.06 | -0.09 – 0.20 | 0.469 | 0.25 | 0.10 – 0.40 | **0.001** | -0.15 | -0.30 – 0.00 | 0.052 | -0.07 | -0.23 – 0.08 | 0.342 |
| Age | -0.05 | -0.23 – 0.13 | 0.601 | 0.02 | -0.15 – 0.20 | 0.813 | 0.05 | -0.12 – 0.22 | 0.582 | -0.08 | -0.25 – 0.10 | 0.394 | 0.04 | -0.14 – 0.22 | 0.683 |
| Female | -0.35 | -0.66 – -0.04 | **0.027** | 0.01 | -0.29 – 0.31 | 0.936 | 0.09 | -0.20 – 0.39 | 0.536 | 0.24 | -0.06 – 0.54 | 0.115 | -0.04 | -0.34 – 0.27 | 0.806 |
| Gender (Other) | 0.11 | -1.13 – 1.36 | 0.856 | 0.48 | -0.72 – 1.69 | 0.431 | -0.02 | -1.21 – 1.17 | 0.974 | -0.21 | -1.43 – 1.00 | 0.731 | -0.28 | -1.51 – 0.96 | 0.658 |
| Education | 0.03 | -0.13 – 0.19 | 0.673 | -0.14 | -0.30 – 0.01 | 0.066 | 0.06 | -0.09 – 0.21 | 0.432 | -0.10 | -0.26 – 0.06 | 0.207 | 0.12 | -0.04 – 0.28 | 0.134 |
| Black | 0.18 | -0.36 – 0.71 | 0.513 | 0.56 | 0.04 – 1.07 | **0.035** | 0.36 | -0.15 – 0.88 | 0.163 | -0.51 | -1.03 – 0.01 | 0.053 | -0.44 | -0.96 – 0.09 | 0.103 |
| Native American | 0.27 | -1.76 – 2.30 | 0.795 | -0.45 | -2.43 – 1.52 | 0.649 | 0.15 | -1.80 – 2.10 | 0.881 | -0.05 | -2.03 – 1.93 | 0.964 | 0.06 | -1.95 – 2.06 | 0.957 |
| Asian | -0.01 | -0.66 – 0.64 | 0.981 | 0.03 | -0.60 – 0.66 | 0.919 | 0.30 | -0.32 – 0.93 | 0.336 | -0.50 | -1.13 – 0.13 | 0.121 | 0.14 | -0.51 – 0.78 | 0.679 |
| Pacific Islander | 0.07 | -0.67 – 0.81 | 0.848 | 0.61 | -0.10 – 1.33 | 0.093 | 0.24 | -0.46 – 0.94 | 0.496 | -0.18 | -0.90 – 0.53 | 0.614 | -0.46 | -1.19 – 0.26 | 0.207 |
| Multi-racial | 0.27 | -0.56 – 1.09 | 0.524 | -0.03 | -0.84 – 0.77 | 0.935 | 0.82 | 0.03 – 1.62 | **0.043** | -0.74 | -1.55 – 0.06 | 0.071 | -0.15 | -0.96 – 0.67 | 0.723 |
| Part-time | -0.10 | -0.60 – 0.39 | 0.681 | 0.20 | -0.29 – 0.68 | 0.425 | 0.08 | -0.39 – 0.56 | 0.729 | -0.02 | -0.51 – 0.46 | 0.926 | -0.12 | -0.61 – 0.37 | 0.628 |
| Self-employed | -0.26 | -0.76 – 0.25 | 0.312 | 0.02 | -0.47 – 0.50 | 0.950 | 0.31 | -0.17 – 0.79 | 0.205 | 0.08 | -0.41 – 0.57 | 0.745 | -0.15 | -0.64 – 0.35 | 0.560 |
| Unemployed | -0.27 | -0.70 – 0.16 | 0.218 | 0.55 | 0.13 – 0.96 | **0.011** | 0.19 | -0.22 – 0.60 | 0.357 | -0.12 | -0.54 – 0.30 | 0.579 | -0.30 | -0.72 – 0.13 | 0.166 |
| Retired | 0.17 | -0.67 – 1.02 | 0.688 | -0.19 | -1.01 – 0.62 | 0.642 | 0.17 | -0.64 – 0.98 | 0.676 | -0.56 | -1.38 – 0.27 | 0.185 | 0.37 | -0.47 – 1.20 | 0.386 |
| Student | 0.21 | -0.47 – 0.88 | 0.547 | 0.25 | -0.40 – 0.90 | 0.447 | 0.27 | -0.38 – 0.91 | 0.411 | -0.08 | -0.74 – 0.58 | 0.807 | -0.44 | -1.10 – 0.23 | 0.194 |
| Employ. (Other) | -0.23 | -1.08 – 0.62 | 0.599 | 0.09 | -0.74 – 0.91 | 0.839 | 0.18 | -0.64 – 1.00 | 0.666 | 0.61 | -0.22 – 1.45 | 0.147 | -0.51 | -1.35 – 0.34 | 0.237 |
| Average WTP | 0.15 | 0.00 – 0.30 | **0.047** | 0.12 | -0.03 – 0.26 | 0.111 | 0.15 | 0.01 – 0.29 | **0.033** | 0.08 | -0.07 – 0.22 | 0.292 | 0.09 | -0.05 – 0.24 | 0.200 |
| Order Authority | -0.03 | -0.18 – 0.12 | 0.656 |  |  |  |  |  |  |  |  |  |  |  |  |
| Order Care |  |  |  | 0.09 | -0.06 – 0.23 | 0.252 |  |  |  |  |  |  |  |  |  |
| Order Fairness |  |  |  |  |  |  | 0.08 | -0.06 – 0.22 | 0.250 |  |  |  |  |  |  |
| Order Loyalty |  |  |  |  |  |  |  |  |  | -0.03 | -0.17 – 0.11 | 0.688 |  |  |  |
| Order Purity |  |  |  |  |  |  |  |  |  |  |  |  | 0.07 | -0.07 – 0.22 | 0.329 |
| Observations | 196 | | | 196 | | | 196 | | | 196 | | | 196 | | |
| R^2^ / R^2^ adjusted | 0.076 / -0.018 | | | 0.135 / 0.047 | | | 0.155 / 0.069 | | | 0.120 / 0.030 | | | 0.098 / 0.006 | | |

**Figure S8**. Distributions of effectiveness and WTP outcome in Study 3b

| Liberal - Conservative (Effectiveness)  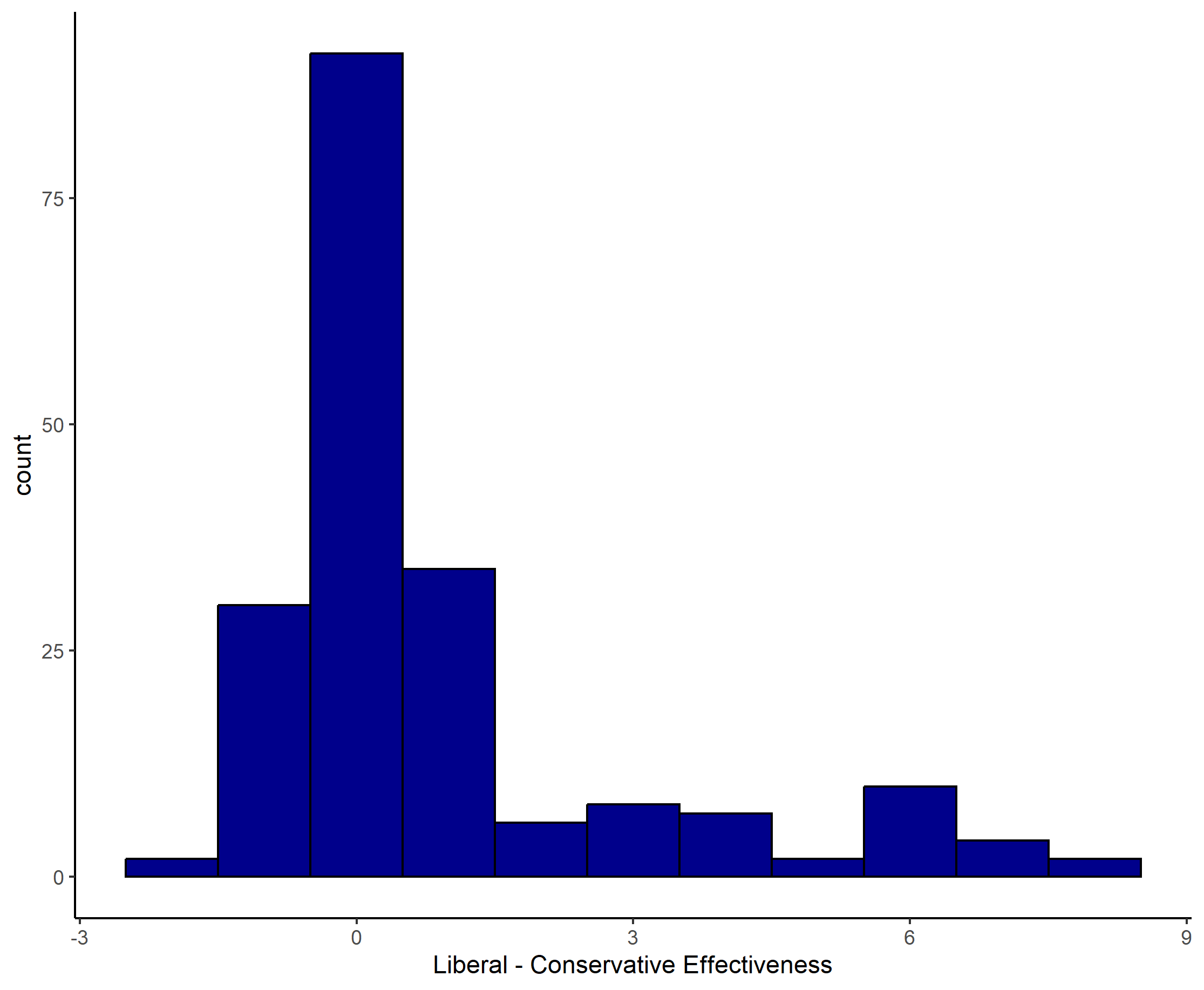 | Liberal - Conservative (WTP)  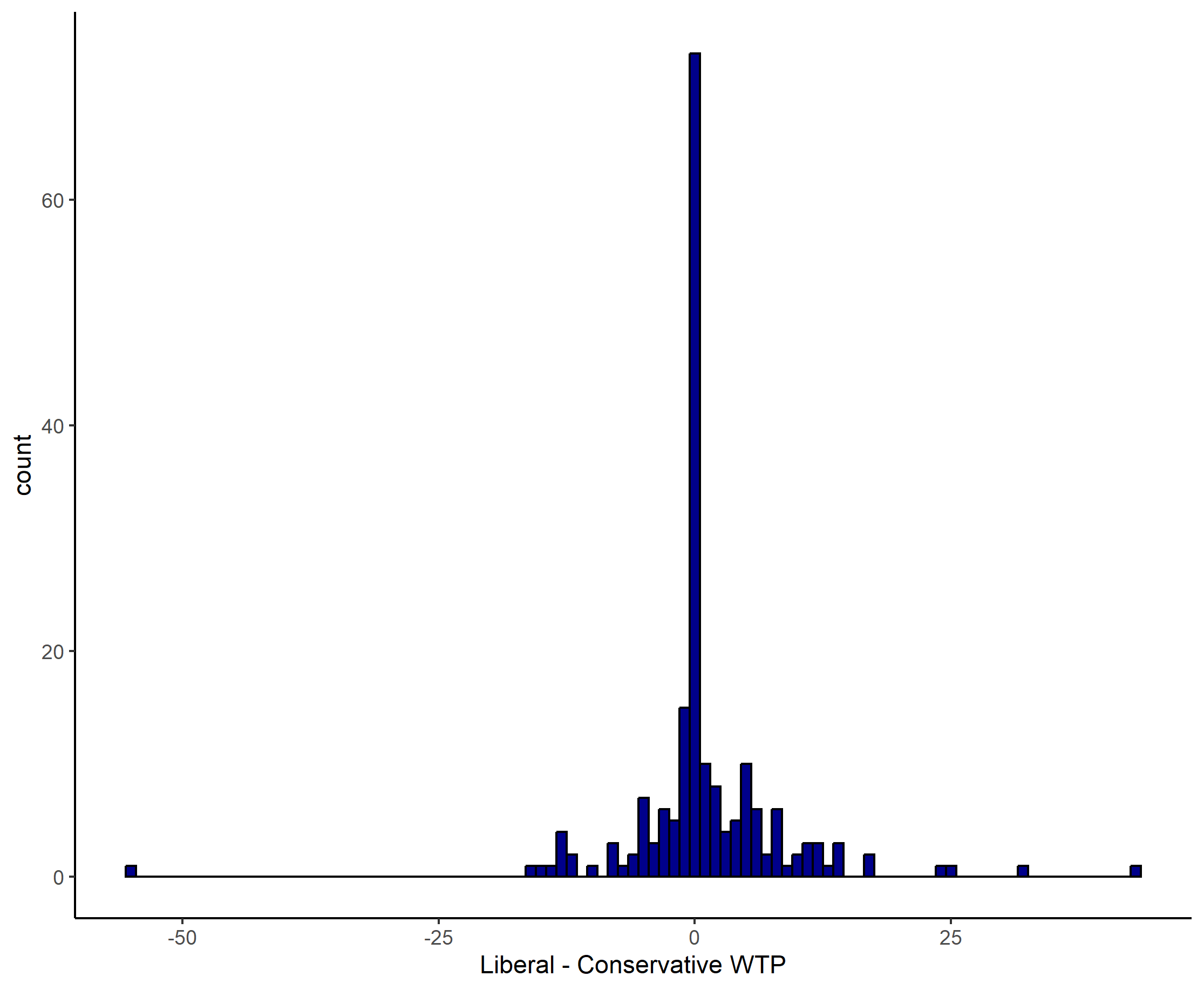 |
| --- | --- |

**Figure S9.** Marginal effects of political ideology on effectiveness and WTP ratings

**
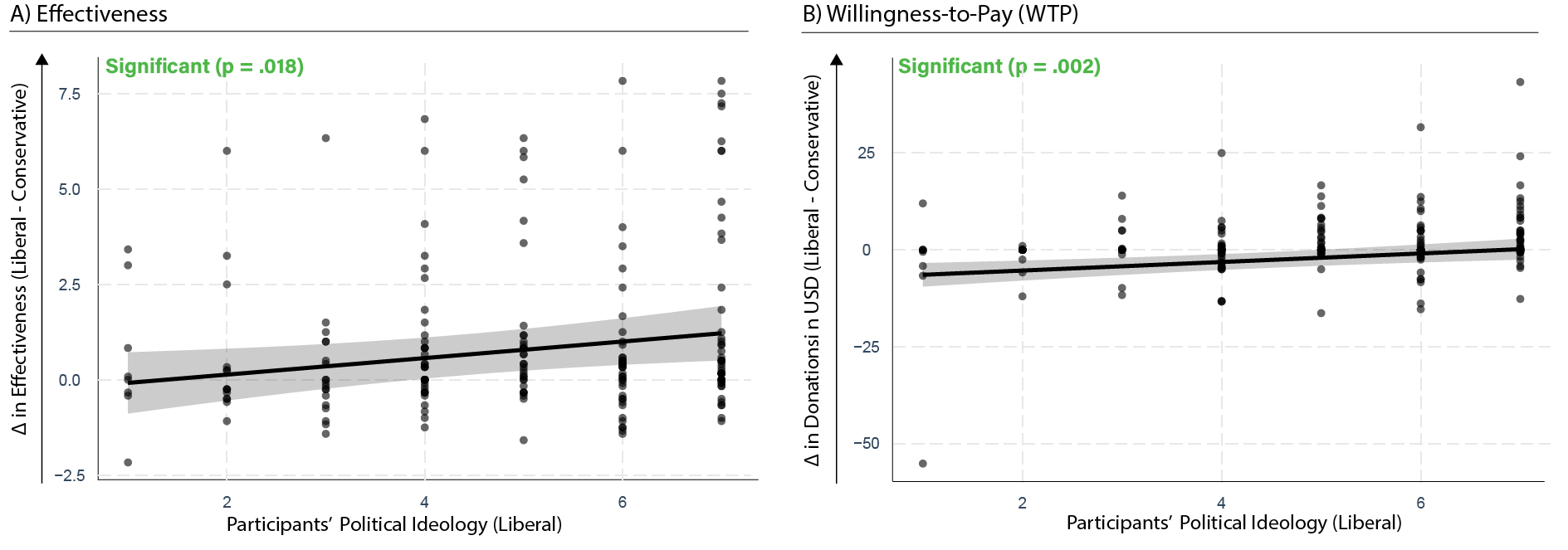
**

**Study 3c**

**Sample demographics**

| Age | 36.1 (12.3SD) |
| --- | --- |
| Gender | 52% Female |
| Ethnicity | 79% White  6% Black or African American  1% American Indian, Native American or Alaska Native  6% Asian  0% Hawaiian Native or Other Pacific Islander  3% Other  6% Multiracial |
| Education | 1% Some high school or less  12% High school diploma or GED  18% Some college but no degree  18% Associates or technical degree  42% Bachelor’s degree  20% Graduate or professional degree (MA, MS, MBA, PHD, JD, MD etc.) |
| Income | 14% Less than $10,000  5% $10,000 to $14,999  9% $15,000 to $24,999  13% $25,000 to $34,999  13% $35,000 to $49,999  19% $50,000 to $74,999  10% $75,000 to $99,999  10% $100,000 to $149,999  5% $150,000 to $199,999  2% $200,000 or more |
| Employment | 54% Employed (full-time)  10% Employed (part-time)  9% Self-employed  12% Unemployed  8% Student  5% Retired  3% Other |

**Table S30.** *Means, standard deviations, and zero-order correlations with 95% CI in Study 3c*

| Variable | *M* | *SD* | 1 | 2 | 3 | 4 | 5 | 6 | 7 | 8 | 9 | 10 | 11 | 12 |
| --- | --- | --- | --- | --- | --- | --- | --- | --- | --- | --- | --- | --- | --- | --- |
|  |  |  |  |  |  |  |  |  |  |  |  |  |  |  |
| 1. Effect. (iPhone) | 3.03 | 1.50 |  |  |  |  |  |  |  |  |  |  |  |  |
|  |  |  |  |  |  |  |  |  |  |  |  |  |  |  |
| 2. WTP (iPhone) | 448.59 | 258.79 | .36** |  |  |  |  |  |  |  |  |  |  |  |
|  |  |  | [.26, .45] |  |  |  |  |  |  |  |  |  |  |  |
|  |  |  |  |  |  |  |  |  |  |  |  |  |  |  |
| 3. Effect. (Political) | 4.78 | 1.41 | .16** | .18** |  |  |  |  |  |  |  |  |  |  |
|  |  |  | [.05, .26] | [.07, .28] |  |  |  |  |  |  |  |  |  |  |
|  |  |  |  |  |  |  |  |  |  |  |  |  |  |  |
| 4. WTP (Political) | 14.95 | 19.22 | .14* | .16** | .46** |  |  |  |  |  |  |  |  |  |
|  |  |  | [.03, .24] | [.05, .26] | [.37, .54] |  |  |  |  |  |  |  |  |  |
|  |  |  |  |  |  |  |  |  |  |  |  |  |  |  |
| 5. EX | 3.73 | 1.20 | .08 | .13* | -.10 | .05 |  |  |  |  |  |  |  |  |
|  |  |  | [-.03, .19] | [.02, .24] | [-.21, .01] | [-.06, .16] |  |  |  |  |  |  |  |  |
|  |  |  |  |  |  |  |  |  |  |  |  |  |  |  |
| 6. Political I. | 4.94 | 1.72 | -.12* | -.01 | .46** | .20** | -.24** |  |  |  |  |  |  |  |
|  |  |  | [-.22, -.01] | [-.12, .10] | [.37, .54] | [.10, .31] | [-.34, -.14] |  |  |  |  |  |  |  |
|  |  |  |  |  |  |  |  |  |  |  |  |  |  |  |
| 7. Age | 36.12 | 13.29 | .06 | -.07 | -.24** | .05 | .16** | -.30** |  |  |  |  |  |  |
|  |  |  | [-.05, .17] | [-.18, .04] | [-.34, -.13] | [-.06, .16] | [.05, .27] | [-.40, -.20] |  |  |  |  |  |  |
|  |  |  |  |  |  |  |  |  |  |  |  |  |  |  |
| 8. OP | 5.18 | 1.04 | -.07 | -.05 | .03 | .05 | .26** | .15** | -.01 |  |  |  |  |  |
|  |  |  | [-.18, .04] | [-.16, .06] | [-.08, .14] | [-.06, .16] | [.15, .36] | [.04, .26] | [-.12, .10] |  |  |  |  |  |
|  |  |  |  |  |  |  |  |  |  |  |  |  |  |  |
| 9. CO | 4.80 | 1.21 | .14* | .16** | -.14* | .01 | .49** | -.28** | .25** | .08 |  |  |  |  |
|  |  |  | [.03, .25] | [.06, .27] | [-.24, -.03] | [-.10, .12] | [.40, .57] | [-.38, -.18] | [.15, .35] | [-.03, .18] |  |  |  |  |
|  |  |  |  |  |  |  |  |  |  |  |  |  |  |  |
| 10. AG | 5.02 | 0.95 | .14* | .13* | .11 | .15** | .27** | -.02 | .18** | .22** | .31** |  |  |  |
|  |  |  | [.03, .24] | [.02, .24] | [-.00, .21] | [.04, .25] | [.17, .37] | [-.13, .09] | [.08, .29] | [.11, .32] | [.21, .41] |  |  |  |
|  |  |  |  |  |  |  |  |  |  |  |  |  |  |  |
| 11. NE | 3.69 | 1.32 | -.01 | -.07 | .15** | .02 | -.46** | .22** | -.33** | -.04 | -.50** | -.28** |  |  |
|  |  |  | [-.12, .10] | [-.18, .04] | [.04, .25] | [-.09, .13] | [-.54, -.37] | [.11, .32] | [-.42, -.23] | [-.15, .07] | [-.58, -.41] | [-.38, -.17] |  |  |
|  |  |  |  |  |  |  |  |  |  |  |  |  |  |  |
| 12. Education | 4.40 | 1.32 | -.19** | .02 | -.08 | -.05 | .20** | -.04 | .10 | .02 | .12* | -.05 | -.12* |  |
|  |  |  | [-.29, -.08] | [-.09, .13] | [-.18, .03] | [-.16, .06] | [.09, .30] | [-.14, .07] | [-.01, .20] | [-.09, .13] | [.01, .22] | [-.16, .06] | [-.23, -.01] |  |
|  |  |  |  |  |  |  |  |  |  |  |  |  |  |  |
| 13. Income | 4.98 | 2.42 | -.07 | .10 | -.07 | .02 | .29** | -.14** | .17** | -.02 | .18** | -.03 | -.27** | .38** |
|  |  |  | [-.17, .04] | [-.01, .20] | [-.18, .04] | [-.09, .13] | [.19, .39] | [-.25, -.04] | [.06, .27] | [-.13, .09] | [.07, .28] | [-.14, .08] | [-.37, -.17] | [.28, .47] |
|  |  |  |  |  |  |  |  |  |  |  |  |  |  |  |

*Note.* *M* and *SD* are used to represent mean and standard deviation, respectively. Values in square brackets indicate the 95% confidence interval for each correlation. * indicates *p* < .05. ** indicates *p* < .01.

**Table S31.** Full model output of linear regression analyses for Effectiveness and WTP for the iPhone and climate action scenario in Study 3c

|  | ***Effectiveness (iPhone)*** | | | ***WTP (iPhone)*** | | | ***Effectiveness (Political)*** | | | ***WTP (Political)*** | | |
| --- | --- | --- | --- | --- | --- | --- | --- | --- | --- | --- | --- | --- |
| *Predictors* | *β* | *CI_95_* | *p* | *β* | *CI_95_* | *p* | *β* | *CI_95_* | *p* | *β* | *CI_95_* | *p* |
| (Intercept) | 0.11 | -0.13 – 0.34 | **0.001** | 0.08 | -0.17 – 0.33 | 0.093 | -0.07 | -0.28 – 0.14 | **<0.001** | -0.11 | -0.35 – 0.12 | 0.111 |
| Participant Extraversion | -0.03 | -0.20 – 0.14 | 0.747 | 0.06 | -0.12 – 0.24 | 0.505 | 0.05 | -0.08 – 0.18 | 0.472 | 0.09 | -0.05 – 0.23 | 0.218 |
| Message Condition (Extraversion) | -0.17 | -0.38 – 0.05 | **0.010** | -0.12 | -0.35 – 0.11 | 0.525 | - | - | - | - | - | - |
| Participant Extraversion x Message Condition | 0.25 | 0.03 – 0.47 | **0.026** | 0.04 | -0.19 – 0.27 | 0.743 | - | - | - | - | - | - |
| Political Ideology (Liberal) | - | - | - | - | - | - | -0.34 | 0.20 – 0.48 | **<0.001** | 0.21 | 0.05 – 0.36 | **0.009** |
| Message Condition (Fairness) | - | - | - | - | - | - | -0.04 | -0.24 – 0.16 | **0.028** | 0.19 | -0.03 – 0.41 | 0.910 |
| Political Ideology × Message Condition | - | - | - | - | - | - | 0.23 | 0.02 – 0.43 | **0.028** | 0.08 | -0.14 – 0.30 | 0.484 |
| Female | 0.07 | -0.17 – 0.31 | 0.586 | -0.04 | -0.29 – 0.21 | 0.771 | 0.16 | -0.06 – 0.38 | 0.155 | 0.13 | -0.12 – 0.37 | 0.303 |
| Gender (Other) | -0.05 | -0.88 – 0.78 | 0.908 | -0.13 | -1.00 – 0.74 | 0.765 | 0.30 | -0.47 – 1.07 | 0.440 | 0.82 | -0.03 – 1.67 | 0.059 |
| Age | 0.01 | -0.13 – 0.15 | 0.862 | -0.14 | -0.29 – 0.01 | 0.060 | -0.13 | -0.26 – 0.01 | 0.061 | 0.14 | -0.00 – 0.29 | 0.052 |
| Openness | -0.12 | -0.23 – -0.01 | **0.040** | -0.11 | -0.23 – 0.01 | 0.067 | -0.06 | -0.17 – 0.05 | 0.283 | -0.02 | -0.14 – 0.10 | 0.756 |
| Conscientiousness | 0.14 | 0.01 – 0.28 | **0.042** | 0.14 | -0.01 – 0.29 | 0.061 | -0.05 | -0.18 – 0.08 | 0.491 | 0.01 | -0.14 – 0.15 | 0.905 |
| Agreeableness | 0.10 | -0.02 – 0.22 | 0.113 | 0.13 | 0.01 – 0.26 | **0.039** | 0.14 | 0.03 – 0.25 | **0.013** | 0.11 | -0.01 – 0.23 | 0.079 |
| Neuroticism | 0.08 | -0.07 – 0.22 | 0.306 | 0.04 | -0.12 – 0.19 | 0.649 | 0.04 | -0.09 – 0.17 | 0.527 | 0.08 | -0.06 – 0.23 | 0.273 |
| Education | -0.19 | -0.31 – -0.07 | **0.003** | -0.02 | -0.15 – 0.10 | 0.728 | -0.06 | -0.17 – 0.05 | 0.307 | -0.11 | -0.24 – 0.01 | 0.070 |
| Black | 0.16 | -0.30 – 0.62 | 0.497 | -0.14 | -0.62 – 0.34 | 0.575 | 0.39 | -0.04 – 0.81 | 0.073 | 0.17 | -0.30 – 0.64 | 0.483 |
| Native American | 1.34 | -0.64 – 3.31 | 0.185 | -0.46 | -2.53 – 1.61 | 0.662 | 1.28 | -0.54 – 3.10 | 0.168 | -0.01 | -2.02 – 2.01 | 0.995 |
| Asian | -0.29 | -0.75 – 0.17 | 0.211 | 0.14 | -0.33 – 0.62 | 0.558 | -0.02 | -0.44 – 0.39 | 0.910 | 0.15 | -0.31 – 0.62 | 0.521 |
| Pacific Islander | -0.20 | -0.83 – 0.43 | 0.532 | 0.01 | -0.64 – 0.67 | 0.972 | 0.24 | -0.34 – 0.82 | 0.416 | -0.01 | -0.65 – 0.63 | 0.974 |
| Multi-racial | 0.03 | -0.44 – 0.51 | 0.886 | 0.21 | -0.28 – 0.70 | 0.405 | 0.08 | -0.36 – 0.51 | 0.732 | 0.13 | -0.36 – 0.61 | 0.610 |
| Part-time | 0.18 | -0.22 – 0.59 | 0.366 | 0.04 | -0.38 – 0.46 | 0.868 | -0.14 | -0.50 – 0.23 | 0.467 | -0.07 | -0.48 – 0.34 | 0.741 |
| Self-employed | -0.60 | -1.01 – -0.19 | **0.004** | 0.06 | -0.37 – 0.49 | 0.784 | -0.03 | -0.41 – 0.35 | 0.884 | -0.40 | -0.82 – 0.02 | 0.062 |
| Unemployed | -0.11 | -0.52 – 0.29 | 0.575 | -0.20 | -0.62 – 0.22 | 0.346 | -0.13 | -0.50 – 0.24 | 0.489 | -0.02 | -0.43 – 0.39 | 0.935 |
| Retired | -0.06 | -0.50 – 0.39 | 0.805 | 0.06 | -0.40 – 0.53 | 0.787 | 0.05 | -0.36 – 0.46 | 0.804 | -0.16 | -0.62 – 0.29 | 0.483 |
| Student | 0.07 | -0.56 – 0.69 | 0.832 | -0.04 | -0.69 – 0.62 | 0.913 | 0.05 | -0.52 – 0.62 | 0.856 | -0.13 | -0.77 – 0.50 | 0.681 |
| Employ. (Other) | 0.07 | -0.61 – 0.74 | 0.849 | 0.08 | -0.63 – 0.78 | 0.828 | -0.55 | -1.18 – 0.07 | 0.082 | -0.55 | -1.24 – 0.14 | 0.119 |
| Income | -0.05 | -0.18 – 0.09 | 0.510 | 0.07 | -0.07 – 0.22 | 0.328 | 0.03 | -0.10 – 0.16 | 0.649 | 0.05 | -0.09 – 0.19 | 0.490 |
| Observations | 320 | | | 320 | | | 320 | | | 320 | | |
| R^2^ / R^2^ adjusted | 0.161 / 0.095 | | | 0.083 / 0.012 | | | 0.293 / 0.235 | | | 0.131 / 0.061 | | |

**Figure S10**. Distributions of Effectiveness and WTP outcome variables in Study 3c

| Extraversion (Effectiveness)  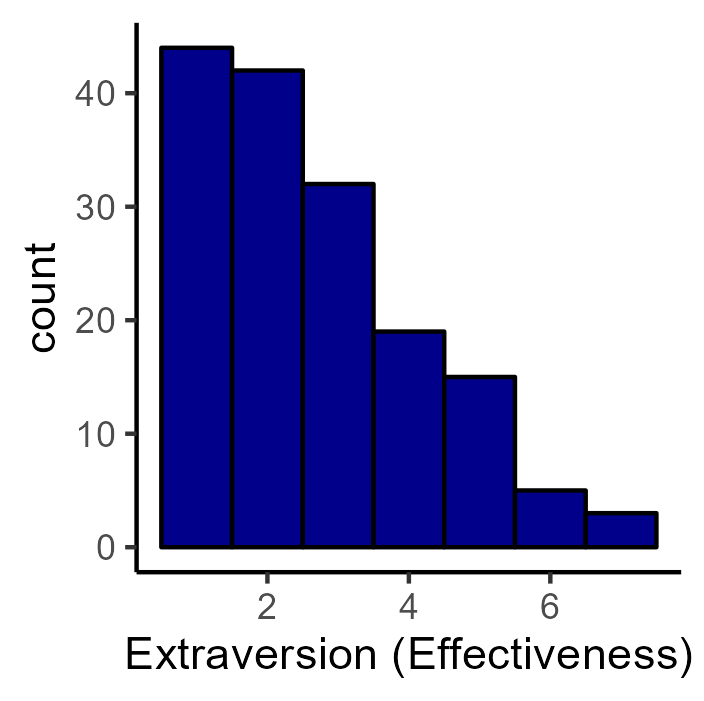 | Extraversion (WTP)  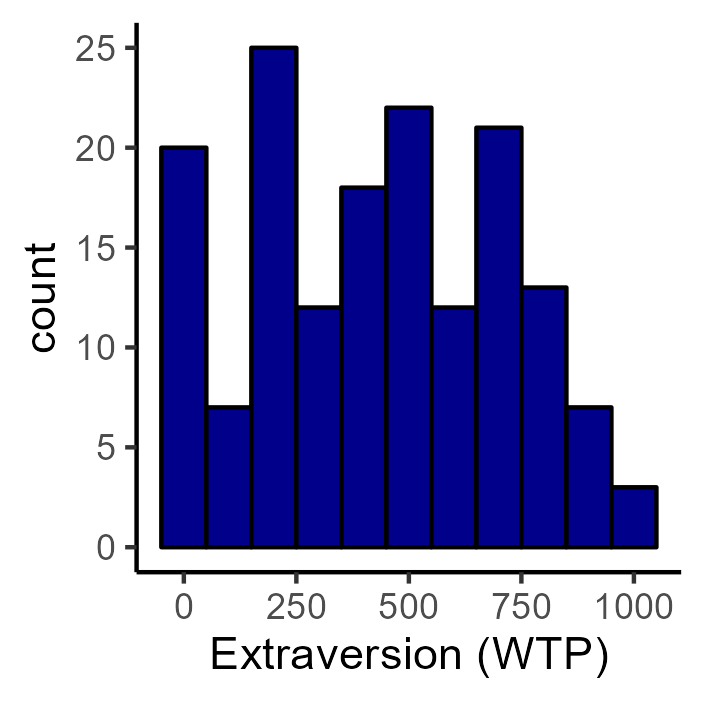 |
| --- | --- |
| Introversion (Effectiveness)  **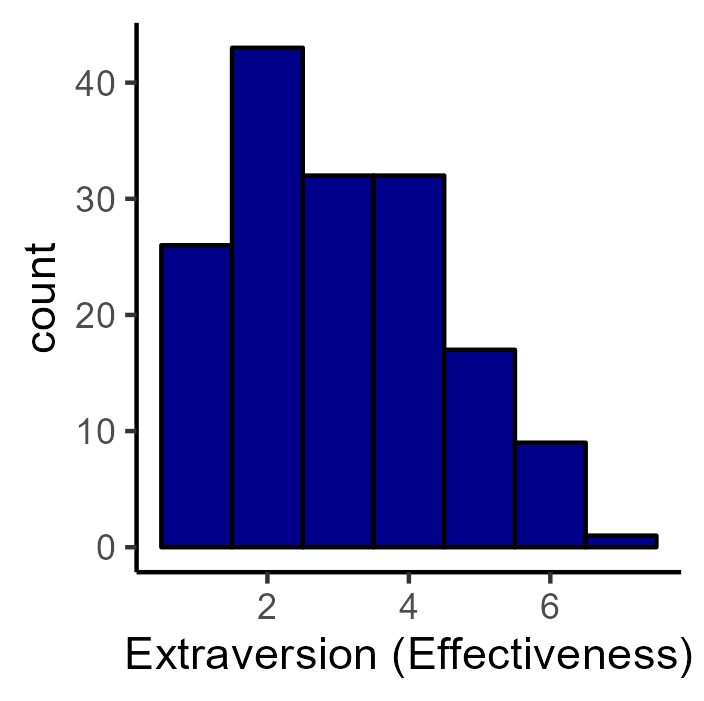** | Introversion (WTP)  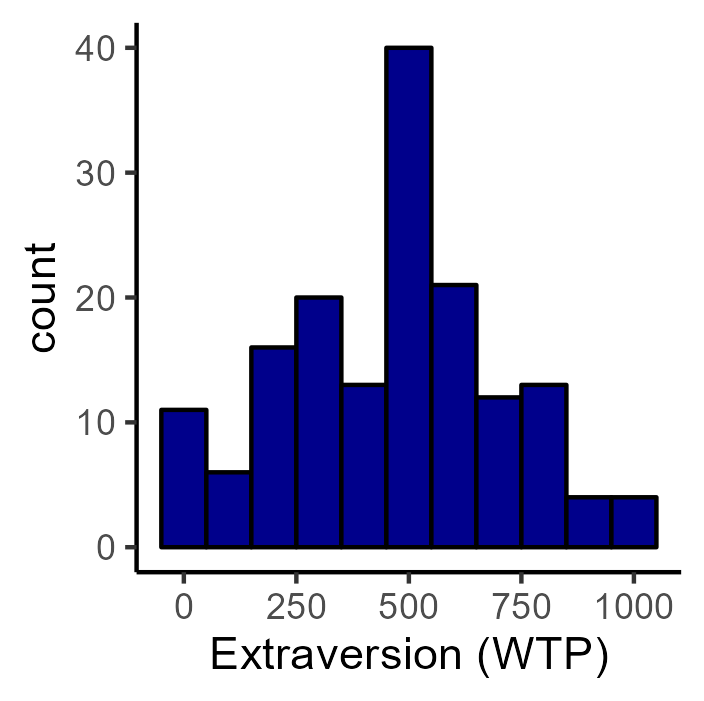 |

| Fairness (Effectiveness)  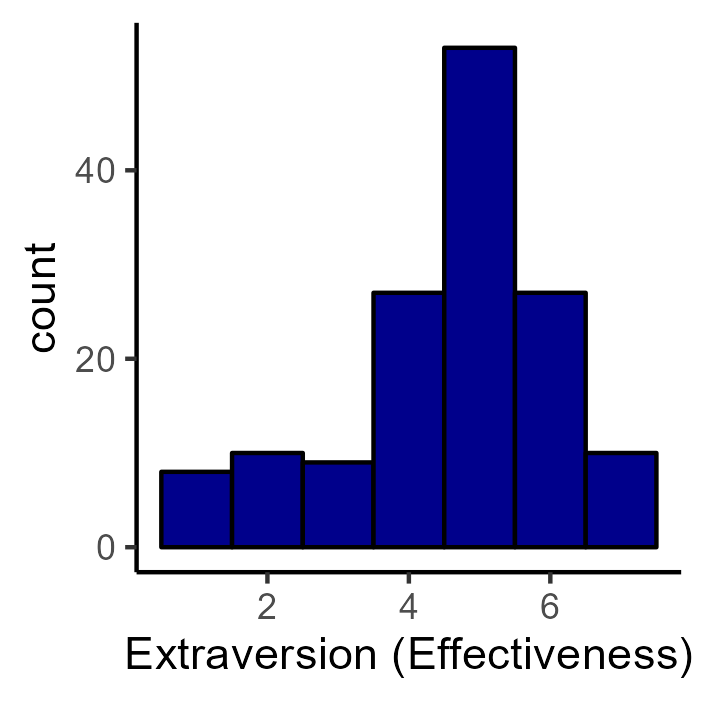 | Fairness (WTP)  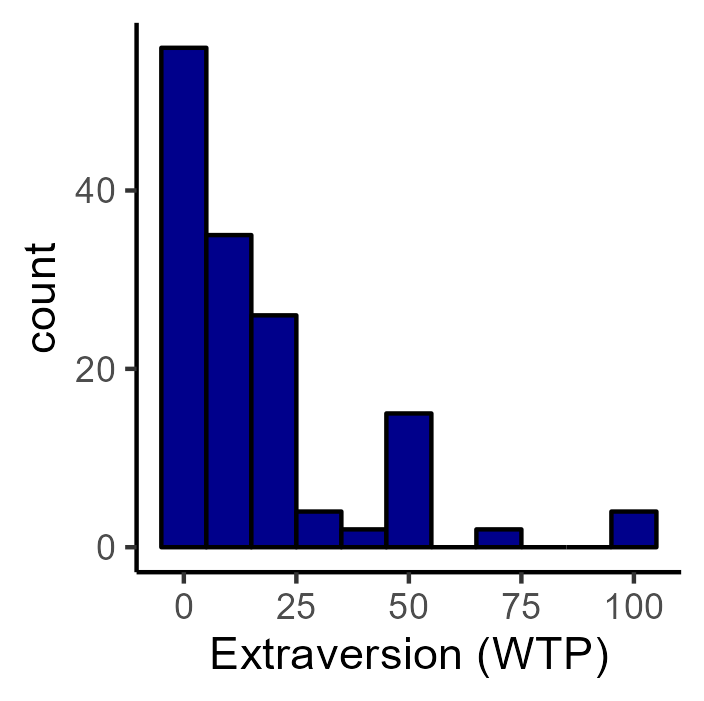 |
| --- | --- |
| Loyalty (Effectiveness)  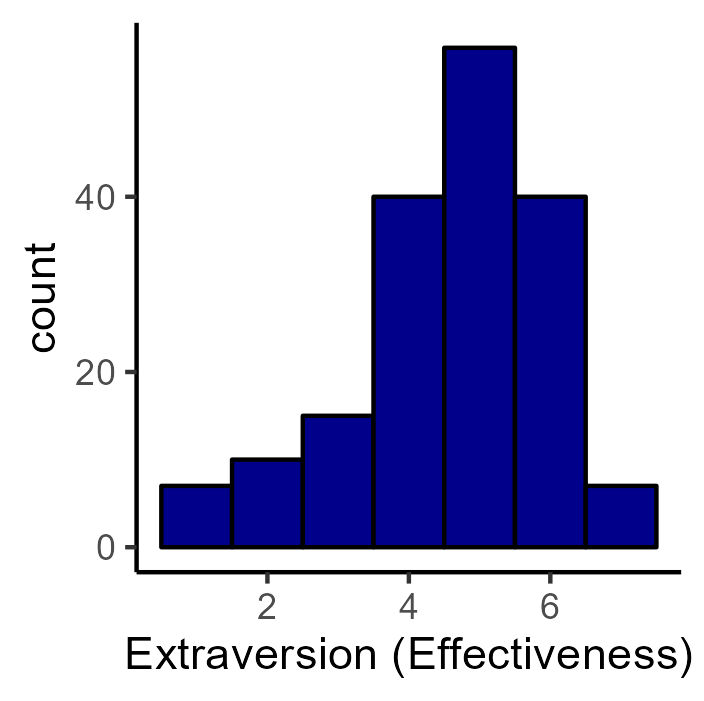 | Loyalty (WTP)  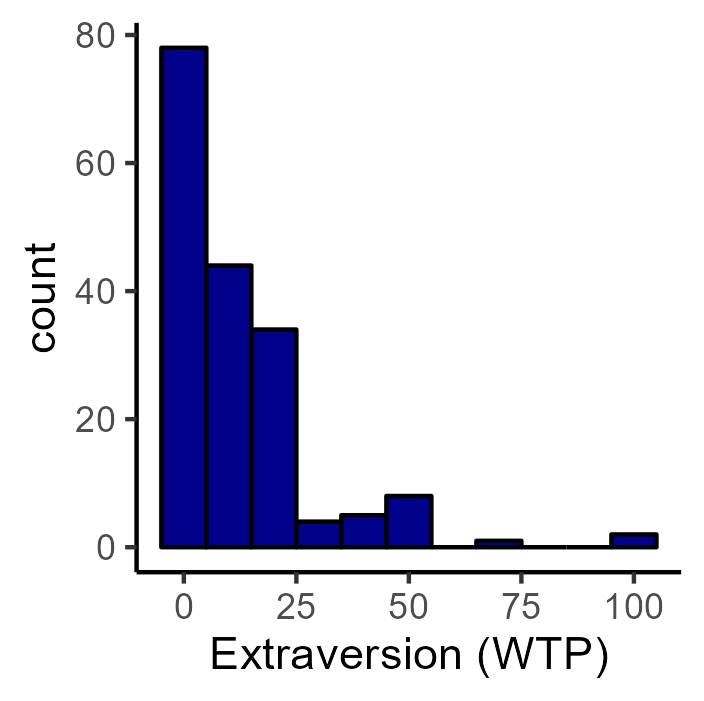 |

**Study 4**

**Sample demographics**

| Age | 43.4 (14.1SD) |
| --- | --- |
| Gender | 48% Female |
| Ethnicity | 82% White  8% Black or African American  0% American Indian, Native American or Alaska Native  5% Asian  0% Hawaiian Native or Other Pacific Islander  1% Other  4% Multiracial |
| Education | 1% Some high school or less  16% High school diploma or GED  21% Some college but no degree  10% Associates or technical degree  37% Bachelor’s degree  15% Graduate or professional degree (MA, MS, MBA, PHD, JD, MD etc.) |
| Income | 17% Less than $10,000  7% $10,000 to $14,999  9% $15,000 to $24,999  12% $25,000 to $34,999  12% $35,000 to $49,999  18% $50,000 to $74,999  13% $75,000 to $99,999  8% $100,000 to $149,999  2% $150,000 to $199,999  2% $200,000 or more |
| Employment | 42% Employed (full-time)  14% Employed (part-time)  11% Self-employed  16% Unemployed  4% Student  9% Retired  4% Other |

**Table S32.** Absolute (and relative) frequencies of personality traits appearing as the most salient trait in Study 4.

|  | Absolute (relative) frequency | |
| --- | --- | --- |
| Trait | Low | High |
| Openness | 46 (15%) | 27 (12%) |
| Conscientiousness | 38 (13%) | 52 (18%) |
| Extraversion | 40 (13%) | 72 (24%) |

**Table S33.** Means, standard deviations, and zero-order correlations with 95% CI of variables in Study 4

| Variable | *M* | *SD* | 1 | 2 | 3 | 4 | 5 | 6 | 7 | 8 | 9 | 10 | 11 | 12 |
| --- | --- | --- | --- | --- | --- | --- | --- | --- | --- | --- | --- | --- | --- | --- |
|  |  |  |  |  |  |  |  |  |  |  |  |  |  |  |
| 1. Effect. (W, Pers) | 4.98 | 1.40 |  |  |  |  |  |  |  |  |  |  |  |  |
|  |  |  |  |  |  |  |  |  |  |  |  |  |  |  |
| 2. Effect. (S, Pers | 4.32 | 1.48 | .48** |  |  |  |  |  |  |  |  |  |  |  |
|  |  |  | [.39, .56] |  |  |  |  |  |  |  |  |  |  |  |
|  |  |  |  |  |  |  |  |  |  |  |  |  |  |  |
| 3. Effect. (W, Generic) | 4.74 | 1.42 | .41** | .46** |  |  |  |  |  |  |  |  |  |  |
|  |  |  | [.31, .50] | [.36, .54] |  |  |  |  |  |  |  |  |  |  |
|  |  |  |  |  |  |  |  |  |  |  |  |  |  |  |
| 4. Effect. (S, Generic) | 4.21 | 1.50 | .49** | .61** | .49** |  |  |  |  |  |  |  |  |  |
|  |  |  | [.40, .57] | [.53, .67] | [.40, .57] |  |  |  |  |  |  |  |  |  |
|  |  |  |  |  |  |  |  |  |  |  |  |  |  |  |
| 5. WTP (W, Pers) | 916.47 | 507.42 | .50** | .30** | .26** | .27** |  |  |  |  |  |  |  |  |
|  |  |  | [.40, .58] | [.19, .40] | [.15, .37] | [.16, .37] |  |  |  |  |  |  |  |  |
|  |  |  |  |  |  |  |  |  |  |  |  |  |  |  |
| 6. WTP (S, Pers) | 67.50 | 32.01 | .30** | .61** | .28** | .42** | .40** |  |  |  |  |  |  |  |
|  |  |  | [.19, .40] | [.53, .68] | [.17, .38] | [.32, .51] | [.30, .49] |  |  |  |  |  |  |  |
|  |  |  |  |  |  |  |  |  |  |  |  |  |  |  |
| 7. WTP (W, Generic) | 65.65 | 31.54 | .30** | .41** | .30** | .59** | .39** | .83** |  |  |  |  |  |  |
|  |  |  | [.19, .40] | [.31, .50] | [.19, .40] | [.51, .66] | [.29, .48] | [.79, .86] |  |  |  |  |  |  |
|  |  |  |  |  |  |  |  |  |  |  |  |  |  |  |
| 8. WTP (S, generic | 65.65 | 31.54 | .30** | .41** | .30** | .59** | .39** | .83** | 1.00** |  |  |  |  |  |
|  |  |  | [.19, .40] | [.31, .50] | [.19, .40] | [.51, .66] | [.29, .48] | [.79, .86] | [1.00, 1.00] |  |  |  |  |  |
|  |  |  |  |  |  |  |  |  |  |  |  |  |  |  |
| 9. OP | 5.22 | 1.09 | .03 | -.06 | .04 | .10 | .08 | .03 | .05 | .05 |  |  |  |  |
|  |  |  | [-.08, .15] | [-.17, .06] | [-.08, .15] | [-.01, .21] | [-.04, .19] | [-.08, .15] | [-.06, .17] | [-.06, .17] |  |  |  |  |
|  |  |  |  |  |  |  |  |  |  |  |  |  |  |  |
| 10. CO | 5.01 | 1.14 | .16** | .16** | .14* | .14* | .13* | .20** | .19** | .19** | .04 |  |  |  |
|  |  |  | [.05, .27] | [.05, .27] | [.03, .25] | [.03, .25] | [.02, .24] | [.08, .30] | [.08, .30] | [.08, .30] | [-.07, .15] |  |  |  |
|  |  |  |  |  |  |  |  |  |  |  |  |  |  |  |
| 11. EX | 3.86 | 1.24 | .15** | .18** | .25** | .19** | .17** | .30** | .26** | .26** | .24** | .40** |  |  |
|  |  |  | [.04, .26] | [.06, .28] | [.14, .35] | [.07, .29] | [.05, .27] | [.19, .40] | [.15, .36] | [.15, .36] | [.13, .34] | [.30, .49] |  |  |
|  |  |  |  |  |  |  |  |  |  |  |  |  |  |  |
| 12. AG | 5.08 | 1.00 | .13* | .21** | .19** | .17** | .13* | .12* | .09 | .09 | .27** | .37** | .25** |  |
|  |  |  | [.02, .25] | [.10, .32] | [.07, .29] | [.06, .28] | [.01, .24] | [.01, .23] | [-.03, .20] | [-.03, .20] | [.16, .37] | [.27, .46] | [.14, .36] |  |
|  |  |  |  |  |  |  |  |  |  |  |  |  |  |  |
| 13. NE | 3.52 | 1.35 | -.01 | -.00 | .01 | .02 | .00 | -.09 | -.11* | -.11* | -.03 | -.49** | -.49** | -.33** |
|  |  |  | [-.12, .10] | [-.12, .11] | [-.10, .12] | [-.10, .13] | [-.11, .12] | [-.20, .03] | [-.22, -.00] | [-.22, -.00] | [-.14, .08] | [-.57, -.40] | [-.57, -.39] | [-.43, -.23] |
|  |  |  |  |  |  |  |  |  |  |  |  |  |  |  |
|  |  |  |  |  |  |  |  |  |  |  |  |  |  |  |

*Note.* *M* and *SD* are used to represent mean and standard deviation, respectively. Values in square brackets indicate the 95% confidence interval for each correlation. Effect. = effectiveness ratings, WTP = willingsness-to-pay, W = Weekend getaway scenario, S = Sneaker scenario, Pers = Personalized ad version, Generic = Generic ad version. OP = Openness, CO = Conscientiousness, EX = Extraversion, AG = Agreeableness, NE = Neuroticism. * indicates *p* < .05. ** indicates *p* < .01.

**Table S34.** Full model output of linear regression analyses for Effectiveness and WTP for the weekend getaway and sneaker scenario with controls in Study 4

|  | **Effectiveness (Weekend)** | | | **Effectiveness (Sneakers)** | | | **WTP (Weekend)** | | | **WTP (Sneakers** | | |
| --- | --- | --- | --- | --- | --- | --- | --- | --- | --- | --- | --- | --- |
| *Predictors* | *β* | *CI_95_* | *p* | *β* | *CI_95_* | *p* | *β* | *CI_95_* | *p* | *β* | *CI_95_* | *p* |
| (Intercept) | -0.13 | -0.34 – 0.08 | **<0.001** | -0.01 | -0.22 – 0.20 | **<0.001** | -0.04 | -0.26 – 0.18 | **<0.001** | -0.01 | -0.22 – 0.21 | **<0.001** |
| Condition [Personalized] | 0.34 | 0.12 – 0.57 | **0.003** | 0.12 | -0.10 – 0.35 | 0.284 | 0.24 | 0.01 – 0.46 | **0.044** | 0.18 | -0.05 – 0.41 | 0.131 |
| Age | 0.09 | -0.05 – 0.23 | 0.217 | 0.15 | 0.01 – 0.29 | **0.035** | 0.15 | 0.00 – 0.29 | **0.043** | 0.24 | 0.10 – 0.38 | **0.001** |
| Female | 0.17 | 0.06 – 0.29 | **0.003** | 0.26 | 0.15 – 0.37 | **<0.001** | 0.05 | -0.07 – 0.17 | 0.395 | 0.02 | -0.10 – 0.13 | 0.794 |
| Black | 0.24 | -0.18 – 0.66 | 0.268 | 0.19 | -0.23 – 0.61 | 0.382 | 0.19 | -0.24 – 0.62 | 0.393 | 0.14 | -0.29 – 0.57 | 0.526 |
| Asian | 0.29 | -0.24 – 0.81 | 0.281 | 0.53 | 0.01 – 1.05 | **0.045** | 0.15 | -0.38 – 0.68 | 0.581 | 0.57 | 0.04 – 1.10 | **0.035** |
| Pacific Islander | 0.68 | -0.29 – 1.66 | 0.169 | 0.80 | -0.18 – 1.77 | 0.108 | 0.01 | -0.99 – 1.01 | 0.985 | 0.44 | -0.55 – 1.43 | 0.384 |
| Multi-racial | 0.24 | -0.35 – 0.84 | 0.426 | 0.32 | -0.27 – 0.91 | 0.287 | 0.50 | -0.11 – 1.11 | 0.110 | 0.01 | -0.59 – 0.62 | 0.970 |
| Education | -0.10 | -0.22 – 0.01 | 0.078 | -0.13 | -0.24 – -0.01 | **0.035** | -0.04 | -0.16 – 0.08 | 0.525 | -0.02 | -0.14 – 0.09 | 0.692 |
| Part-time | -0.10 | -0.45 – 0.25 | 0.571 | -0.05 | -0.40 – 0.30 | 0.774 | -0.12 | -0.47 – 0.24 | 0.523 | 0.05 | -0.30 – 0.40 | 0.786 |
| Self-employed | 0.04 | -0.33 – 0.42 | 0.831 | -0.19 | -0.57 – 0.18 | 0.309 | -0.06 | -0.44 – 0.32 | 0.758 | -0.27 | -0.65 – 0.11 | 0.166 |
| Unemployed | -0.28 | -0.62 – 0.07 | 0.114 | -0.21 | -0.56 – 0.13 | 0.220 | -0.32 | -0.67 – 0.04 | 0.078 | -0.10 | -0.45 – 0.25 | 0.571 |
| Retired | -0.15 | -0.82 – 0.51 | 0.649 | 0.11 | -0.55 – 0.77 | 0.745 | -0.42 | -1.10 – 0.26 | 0.228 | 0.32 | -0.36 – 1.00 | 0.353 |
| Student | 0.03 | -0.43 – 0.50 | 0.889 | -0.27 | -0.73 – 0.20 | 0.256 | -0.17 | -0.64 – 0.31 | 0.497 | -0.77 | -1.24 – -0.29 | **0.002** |
| Employ. (Other) | -0.96 | -1.58 – -0.34 | **0.002** | -0.72 | -1.34 – -0.11 | **0.021** | -0.36 | -0.99 – 0.27 | 0.266 | -0.52 | -1.15 – 0.10 | 0.100 |
| Observations | 297 | | | 297 | | | 297 | | | 297 | | |
| R^2^ / R^2^ adjusted | 0.110 / 0.066 | | | 0.117 / 0.073 | | | 0.066 / 0.020 | | | 0.083 / 0.038 | | |

**Figure S11**. Distributions of Effectiveness and WTP outcome variables in Study 4

| Weekend Getaway Personalized (Effectiveness)  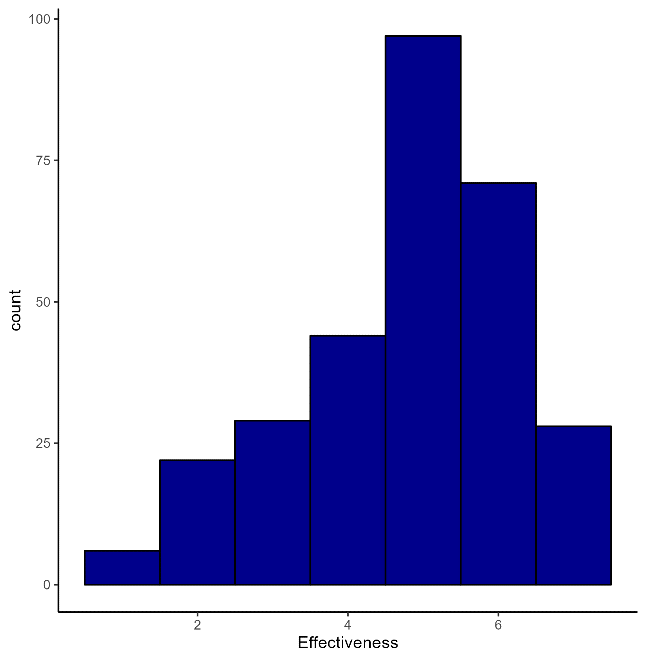 | Weekend Getaway Generic  (Effectiveness)  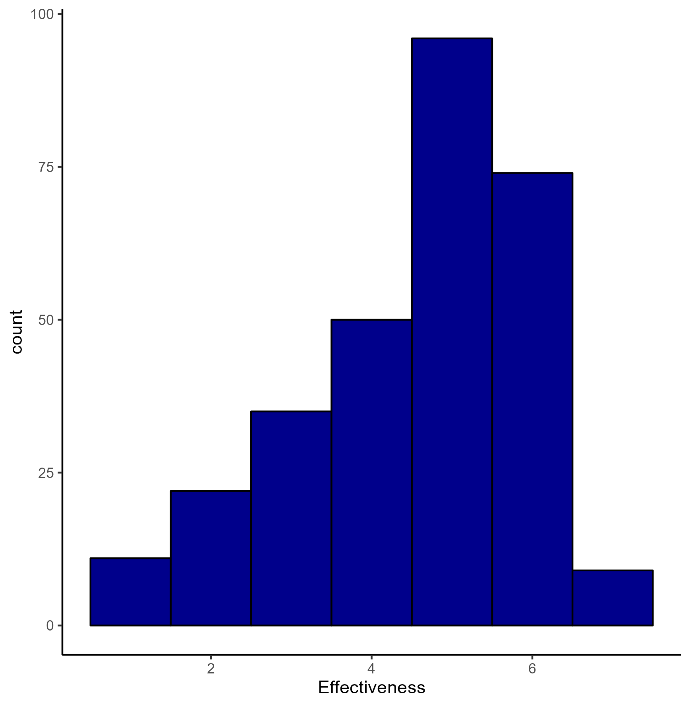 |
| --- | --- |
| Weekend Getaway Personalized (WTP)  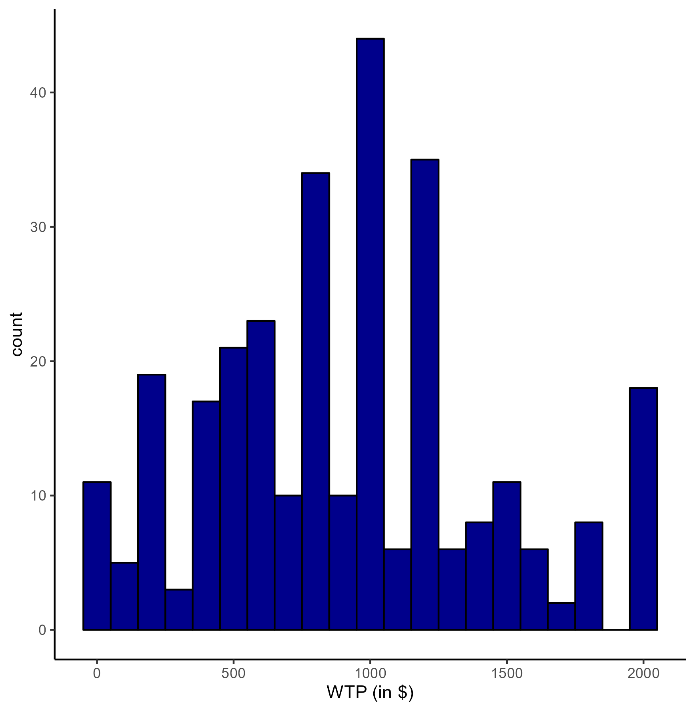 | Weekend Getaway Generic (WTP)  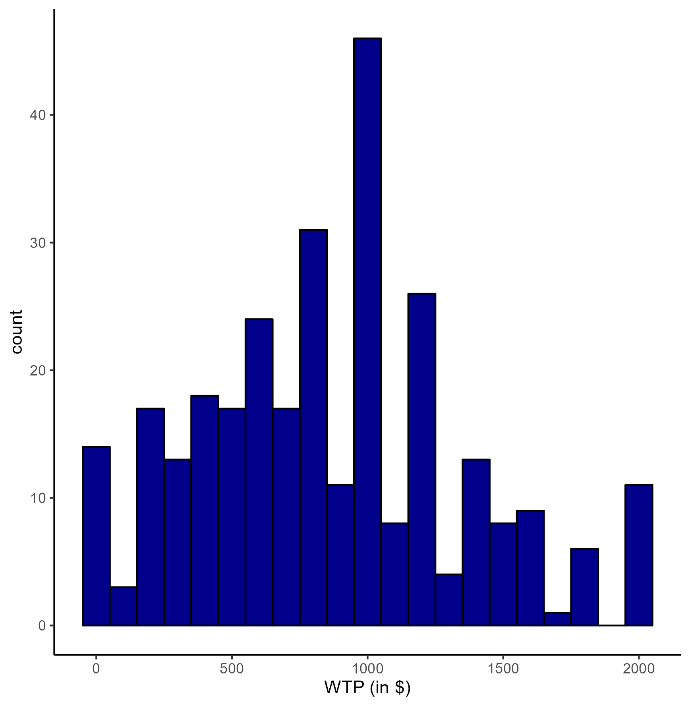 |
| Sneakers Personalized (Effectiveness)  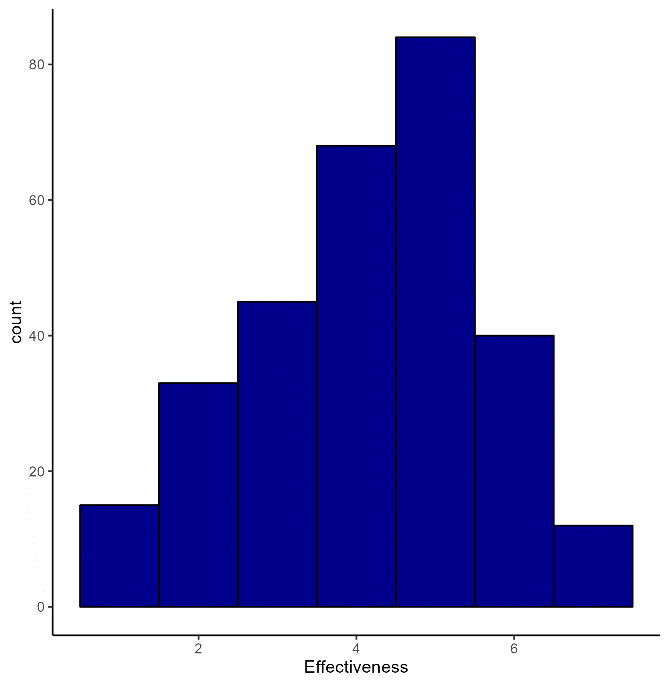 | Sneakers Generic (Effectiveness)  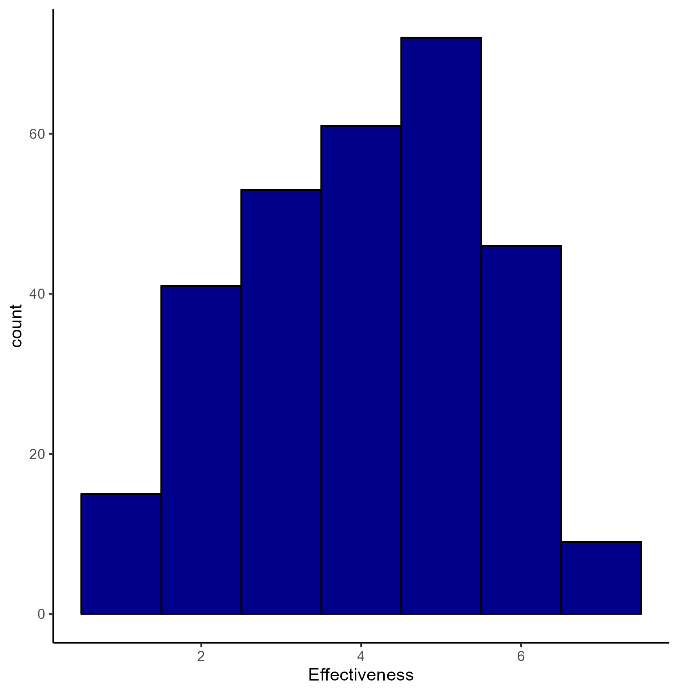 |
| Sneakers Personalized (WTP)  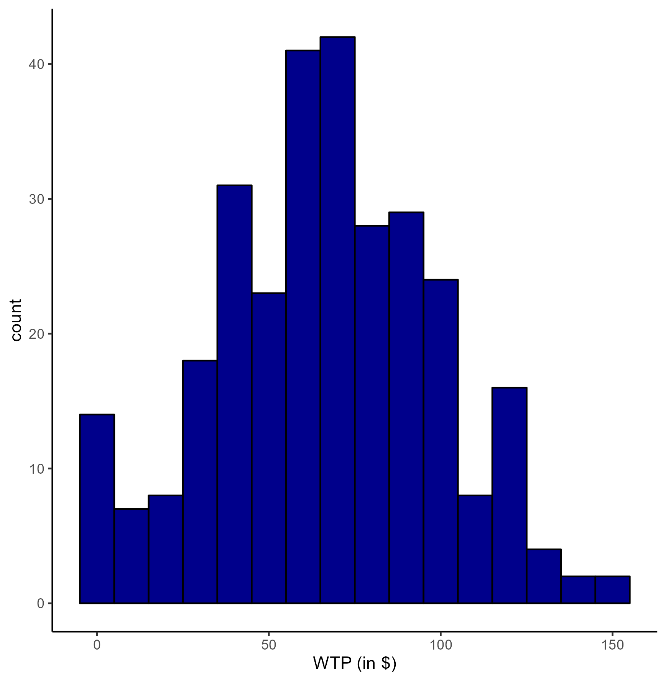 | Sneakers Generic (WTP)  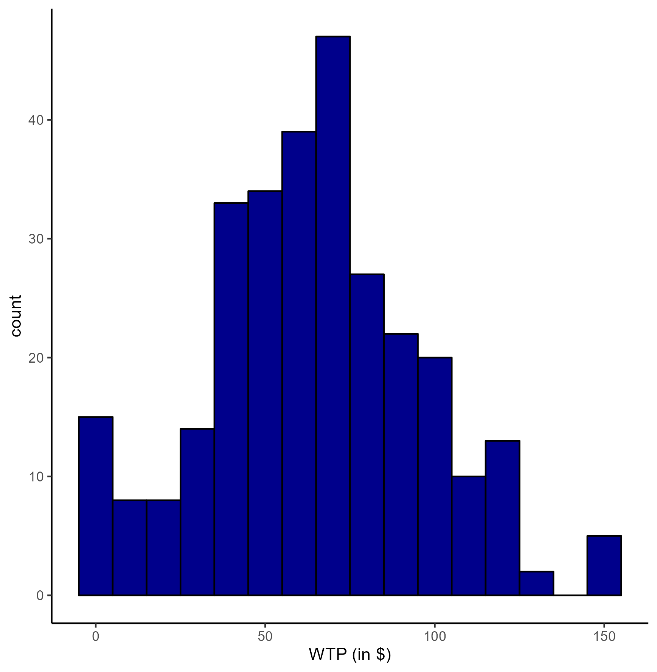 |
